# What is the volume, quality and characteristics of evidence relating to the effectiveness and cost‐effectiveness of multi‐disciplinary occupational health interventions aiming to improve work‐related outcomes for employed adults? An evidence and gap map of systematic reviews

**DOI:** 10.1002/cl2.1412

**Published:** 2024-05-14

**Authors:** Elizabeth Shaw, Michael Nunns, Stuart G. Spicer, Hassanat Lawal, Simon Briscoe, G. J. Melendez‐Torres, Ruth Garside, Kristin Liabo, Jo Thompson Coon

**Affiliations:** ^1^ Exeter Policy Research Programme Evidence Review Facility, Faculty of Health and Life Sciences University of Exeter Exeter UK; ^2^ NIHR Applied Research Collaboration University of Plymouth Plymouth UK

## Abstract

**Background:**

In the UK, tens of millions of working days are lost due to work‐related ill health every year, costing billions of pounds. The role of Occupational Health (OH) services is vital in helping workers to maintain employment when they encounter injury or illness. OH providers traditionally rely on a clinical workforce to deliver these services, particularly doctors and nurses with OH qualifications. However, the increasing demand for OH services is unlikely to be met in the future using this traditional model, due to the declining number of OH‐trained doctors and nurses in the UK. Multi‐disciplinary models of OH delivery, including a more varied range of healthcare and non‐healthcare professionals, could provide a way to meet this new demand for OH services. There is a need to identify collaborative models of OH service delivery and review their effectiveness on return‐to work outcomes. There is an existing pool of systematic review evidence evaluating workplace based, multi‐disciplinary OH interventions, but it is difficult to identify which aspects of the content and/or delivery of these interventions may be associated with improved work‐related outcomes.

**Objectives:**

The aim of this evidence and gap map (EGM) was to provide an overview of the systematic review evidence that evaluates the effectiveness and cost‐effectiveness of multi‐disciplinary OH interventions intending to improve work‐related outcomes.

**Search Methods:**

In June 2021 we searched a selection of bibliographic databases and other academic literature resources covering a range of relevant disciplines, including health care and business studies, to identify systematic review evidence from a variety of sectors of employment. We also searched Google Search and a selection of topically relevant websites and consulted with stakeholders to identify reports already known to them. Searches were updated in February 2023.

**Selection Criteria:**

Systematic reviews needed to be about adults (16 years or over) in employment, who have had absence from work for any medical reason. Interventions needed to be multi‐disciplinary (including professionals from different backgrounds in clinical and non‐clinical professions) and designed to support employees and employers to manage health conditions in the workplace and/or to help employees with health conditions retain and/or return to work following medical absence. Effectiveness needed to be measured in terms of return to work, work retention or measures of absence, or economic evaluation outcomes. These criteria were applied to the title and abstract and full text of each systematic review independently by two reviewers, with disagreements resolved through discussion. We awarded each systematic review a rating of ‘High’, ‘Medium’ or ‘Low’ relevance to indicate the extent to which the populations, interventions and their contexts synthesised within the review were consistent with our research question. We also recorded the number of primary studies included within each of the ‘High’ and ‘Medium’ reviews that were relevant to research question using the same screening process applied at review level.

**Data Collection and Analysis:**

Summary data for each eligible review was extracted. The quality of the systematic reviews, rated as ‘High’ or ‘Medium’ relevance following full text screening, was appraised using the AMSTAR‐2 quality appraisal tool. All data were extracted by one reviewer and checked by a second, with disagreements being settled through discussion. Summary data for all eligible systematic reviews were tabulated and described narratively. The data extracted from reviews of ‘High’ and ‘Medium’ relevance was imported into EPPI‐Mapper software to create an EGM.

**Stakeholder Involvement:**

We worked alongside commissioners and policy makers from the Department of Health and Social Care (DHSC) and Department of Work and Pensions (DWP), OH personnel, and people with lived experience of accessing OH services themselves and/or supporting employees to access OH services. Individuals contributed to decision making at all stages of the project. This ensured our EGM reflects the needs of individuals who will use it.

**Main Results:**

We identified 98 systematic reviews that contained relevant interventions, which involved a variety of professionals and workplaces, and which measured effectiveness in terms of return to work (RTW). Of these, we focused on the 30 reviews where the population and intervention characteristics within the systematic reviews were considered to be of high or medium relevance to our research questions. The 30 reviews were of varying quality, split evenly between High/Moderate quality and Low/Critically‐Low quality ratings. We did not identify any relevant systematic review evidence on any other work‐related outcome of interest. Interventions were heterogenous, both within and across included systematic reviews. The EGM is structured according to the health condition experienced by participants, and the effectiveness of the interventions being evaluated, as reported within the included systematic reviews. It is possible to view (i) the quality and quantity of systematic review evidence for a given health condition, (ii) how review authors assessed the effectiveness or cost‐effectiveness of the interventions evaluated. The EGM also details the primary studies relevant to our research aim included within each review.

**Authors’ Conclusions:**

This EGM map highlights the array of systematic review evidence that exists in relation to the effectiveness or cost‐effectiveness of multi‐disciplinary, workplace‐based OH interventions in supporting RTW. This evidence will allow policy makers and commissioners of services to determine which OH interventions may be most useful for supporting different population groups in different contexts. OH professionals may find the content of the EGM useful in identifying systematic review evidence to support their practice. The EGM also identifies where systematic review evidence in this area is lacking, or where existing evidence is of poor quality. These may represent areas where it may be particularly useful to conduct further systematic reviews.

## 1 PLAIN LANGUAGE SUMMARY

### 1.1 Evidence and gap map of occupational health services to support return to work (for adults) following sickness identifies implications for practice and priorities for research

#### 1.1.1 Background

Occupational Health services play an important role in helping employed people who are unwell or living with a disability to stay in work. These services are currently mainly run by doctors and nurses with Occupational Health qualifications. The number of people with these qualifications is going down, whilst demand for their services is increasing.

There is already research on how well Occupational Health teams work together, to support employed adults to return to work, and if they provide value for money. However, this research doesn't tell us which combination of professionals working together, or which activities they do, results in people returning to work more quickly.

#### 1.1.2 What we want to know?

We are interested in the quantity and quality of existing research which looks at how well Occupational Health activities support employed adults to return to work following a period of sick leave. We are also interested in research which looked at whether these activities provide value for money to the people delivering them.

#### 1.1.3 What is an evidence and gap map?

Our evidence and gap map (EGM) gives a visual summary of existing research on how well different models of Occupational Health services work, to help people with different health conditions return to work. The EGM summarises the amount and quality of research on this topic for different health conditions.

#### 1.1.4 What studies are included?

Our evidence and gap map includes systematic reviews. Systematic reviews bring together research which has already been published on a topic relating to a specific research question. We sought systematic reviews which focused on research looking at how well strategies carried out by teams of different professionals support people to return to work, and if these are worth the money they cost.

We included research that was published in English and focused on working adults (aged 16 and over). To be included, the strategies to support people to return to work needed to be delivered by more than one professional and have some link to the workplace.

#### 1.1.5 What are the main findings of this evidence and gap map?

We included 98 systematic reviews. Thirty of these were judged to be of most relevance to our research question and are presented in the evidence and gap map.

The map shows the amount and quality of systematic review evidence for each health condition and how well the strategies evaluated within them were reported to work. The types of strategies included in the systematic reviews varied widely, as did the quality of the reviews themselves. The most common health conditions represented in the evidence and gap map were musculoskeletal problems such as back pain.

#### 1.1.6 What do the findings of the map mean?

This evidence and gap map provides information for policy makers and health professionals commissioning or delivering occupational health interventions. It also indicates a need for more research relating to people living with cardiac conditions, cancer, stroke and skin problems.

#### 1.1.7 How up to date is this evidence and gap map?

The authors searched for systematic reviews published from 2001 to 21^st^ February 2023.

## 2 BACKGROUND

### 2.1 Introduction

In the UK, around 19.5% of working age adults have a disability (Office for National Statistics, 2021) and approximately 42% of the 50–64 year olds within the UK live with a chronic condition (The Council for Work and Health & Syngentis, 2014). Two‐thirds of long‐term sickness absence has been attributed to common health problems such as musculoskeletal, mental health and cardio‐respiratory conditions (Waddell et al., 2008) Overall in the UK during 2017/2018, over 38 million working days were lost due to work‐related ill health, with nearly £10 billion annual costs attributable to new cases in 2019/2020 (Health and Safety Executive, n.d.). Approximately eight million working age people were registered disabled before the COVID‐19 pandemic. Of these around 50% were in employment, compared to over 80% of non‐disabled people (Office for National Statistics, 2021).

The aging UK population (NHS Confederation, 2017), accompanied by the removal of default retirement age (Department for Business, 2011), increased prevalence of chronic conditions and comorbidities (The Council for Work and Health, 2016) and concerns regarding the impact of the COVID‐19 pandemic, (Burdorf et al., 2020; Giorgi et al., 2020; Godeau et al., 2021; Sinclair et al., 2020) means there is an increased demand for workforce‐based support to enable individuals to continue their productive working lives for as long as they choose. Workplace‐led interventions can also help ensure the next generation of workers are healthier, thus remaining fit for work, by reducing the occurrence of work‐based harms and the impact of lifestyle challenges such as smoking and obesity (The Council for Work and Health, 2016; The Council for Work and Health & Syngentis, 2014). In addition to economic benefits, increased time in employment has been associated with improved mental and physical health, participation in work and social activities and reduced use of healthcare services. A recent population‐based study showed that employment status had a larger moderating effect on personal wellbeing than factors such as age, gender, ethnicity and education (Emerson et al., 2020). The recent COVID‐19 pandemic is also likely to have implications for the workforce, both in terms of increased prevalence of mental ill‐health (Vindegaard & Benros, 2020), and ‘long‐Covid’ symptoms (Mandal et al., 2020), and changes to working patterns, which may affect the support requirements of employees (Kniffin et al., 2021).

#### 2.1.1 Role of occupational health (OH) services

OH services ensure that workplaces meet the physical and mental health needs of their employees (Yogarajah, 2019). Whilst there is no internationally agreed definition of ‘OH services’ (Hassard et al., 2021), their role can include advising employers on preventing work‐related illness, fitness to work and reasonable work‐adjustments. These services are traditionally mostly delivered by clinical staff, particularly OH‐trained doctors and nurses (Tindle et al., 2020), but can involve multi‐disciplinary teams (MDTs). These can consist of a combination of both healthcare and non‐healthcare professionals including, but not limited to, doctors, nurses Occupational Therapists, physiotherapists and OH technicians (The Council for Work and Health, 2016). However, the number of clinical OH specialists available is insufficient to meet current demand for services (The Council for Work and Health, 2016), and could be a barrier to measures aiming to expand access to OH amongst the working population.

To ensure that OH services meet the changing needs of the future workforce, commissioners of OH services will require continued support and guidance from OH leads to inform their decisions (The Council for Work and Health, 2016), with additional support being devoted to help employers not currently commissioning OH services to understand the benefits of OH and what multidisciplinary OH teams can provide. Whilst much healthcare is provided by the NHS, many OH services are not, with OH service provision needing to span work and healthcare settings (The Council for Work and Health & Syngentis, 2014) and take into consideration the decline in the number of OH doctors and nurses. Reviewing existing evidence regarding the effectiveness of multi‐disciplinary OH interventions on return‐to‐work outcomes, including delivery mechanisms, will help inform the needs of those commissioning future OH services and be used by OH providers to expand OH market capacity.

### 2.2 Existing evidence: systematic reviews and grey literature

There is an abundance of systematic review evidence evaluating single and multi‐component OH interventions which aim to improve work and health‐based outcomes, although it is difficult to identify which aspects of the content and/or delivery of these interventions may be associated with success (Gensby et al., 2014).

### 2.3 Why it is important to develop the evidence and gap map (EGM)?

An EGM provides an overview of the quantity, quality and nature of systematic review evidence which already exists in this area (White et al., 2020). The map will allow us to summarise key dimensions of the evidence base. The interactive features of the EGM will enable users to identify and access the evidence most relevant to their requirements. Using the large quantity of existing systematic review evidence relating to OH services to answer our research aims and objectives reduces research waste.

## 3 OBJECTIVES

To provide an overview of the systematic review evidence that has assessed the effectiveness and cost‐effectiveness of multi‐disciplinary OH interventions aiming to improve work‐related outcomes, including return to work (RTW) and reduced sickness absence.

Our research question is as follows:

What is the volume, quality and characteristics of evidence relating to the effectiveness and cost‐effectiveness of multi‐disciplinary OH interventions aiming to improve work‐related outcomes for employed adults?

## 4 METHODS

The methods used to produce this EGM were incorporated into the protocol for a wider umbrella review (Shaw et al., 2022), which was finalised with stakeholders before commencement of this piece of work and registered on the Open Science Framework (Shaw et al., 2022).

### 4.1 Stakeholder engagement

We worked alongside a variety of stakeholders and advisors to ensure our EGM reflects the needs of individuals who will use it. Stakeholders included commissioners and policy makers from the Department of Health and Social Care (DHSC) and the Department of Work and Pensions (DWP), OH personnel (including nurses and occupational physicians) and people with lived experience of accessing OH services themselves and/or supporting employees to access OH services. We met with each group of stakeholders separately to ensure they felt comfortable talking about issues relevant to them. Each stakeholder group was reassured that the specific details regarding what was discussed would remain confidential and we requested that they only provide information they felt comfortable sharing.

Meetings with individuals with lived experience of accessing, and/or supporting others to access OH services were arranged by a co‐ordinator for the Exeter PenARC Patient Engagement Group (PenPEG), who provided existing members of PenPEG with summary details and requested people to contact her if they were interested in taking part in two PPI sessions. They then set‐up and facilitated the first meeting between four individuals from PenPEG and the lead author of this review (LS). During the first online meeting, the co‐ordinator supported members of the public to share their experiences of accessing OH services and facilitated discussion around key topics to inform review progress which had been identified by LS to before the meeting. Due to prior working relationships on this project and others, the second meeting between the lead author of this review and PenPEG members was unfacilitated. In the second online meeting, the reviewer shared the EGM and asked for feedback on what they liked and what was unclear. The impact these discussions had on the review is highlighted in Table 1.

**Table 1:** Impact of stakeholder involvement on review.

Stage of review	Stakeholder [mode of contact, no. people present]	Influence on review process	Specific impact on systematic review
Protocol development	DHSC and DWP [Group meetings/email, >4] Project co‐applicant with lived experience of accessing OH services, both as an employee and as a manager [email]	Stakeholders informed the development of the protocol, including: – Clarifying the aims/objectives; – Identifying key inclusion criteria; – Identifying key outcomes of interest; – Outlining desired impact of review; – Outlining plan for further stakeholder and PPI engagement.	Collaborative development of review protocol which was agreed before commencement of the review
Screening	DHSC and DWP [Group meetings/email, >4] Occupational health personnel [Group meeting, 3]	Stakeholders supported the application of review inclusion criteria to systematic reviews where eligibility for inclusion was uncertain. Provided with opportunity to comment on relevance ratings for systematic reviews	
Data extraction	DHSC and DWP [Group meetings, >4] Occupational health personnel [Group meeting, 3] People with lived experience of accessing OH services as an employee and/or manager [Group meeting, 4 people]	Supported the identification of key data to be extracted from High/Medium relevance systematic reviews	Identification of data regarding intervention characteristics and context of delivery to be extracted Identified additional outcome data to be collected, particularly wellbeing outcomes
Synthesis/Presentation of findings	DHSC and DWP [Draft report, email, face to face meeting, 1] Occupational health personnel [Individual meeting, 1] People with lived experience of accessing OH services as an employee and/or manager [Group meeting, 4]	Commented on accessibility and usefulness of evidence and gap map Highlighted importance of contextual information (i.e., service setting, staffing, employee needs) for understanding the impact, content and delivery of intervention	Priorities of review commissioners informed how the evidence and gap map was structured and the provision of links to the relevant primary studies included within systematic reviews displayed in the evidence and gap map Relabelling of axis in evidence and gap map
Dissemination	People with lived experience of accessing OH services as an employee and/or manager [Group meeting, 4]	Discussed how format of report could be adapted to share with audiences who would be interested in our findings	Supported the identification of relevant audiences with whom we could share our findings

Abbreviations: DHSC, Department of Health and Social Care; DWP, Department of Work and Pensions; No, Number; OH, occupational health; PPI, patient and public involvement.

### 4.2 Dimensions

#### 4.2.1 Types of study design

This EGM includes:

– Systematic reviews of effectiveness studies, whether randomised, non‐randomised or observational;
– Mixed methods systematic reviews;
– Systematic reviews of reviews;
– Rapid reviews which include a synthesis of effectiveness;
– Cost effectiveness reviews.

We included systematic reviews focused on quantitative evidence because they summarise evidence on the effectiveness and cost‐effectiveness of workplace‐based, multi‐disciplinary OH interventions. To be eligible for inclusion systematic reviews needed to meet the minimum quality criteria for the Database of Abstracts of Reviews of Effects (DARE, 1995), that is, they needed to satisfy all of the following:

– Report adequate inclusion/exclusion criteria;
– Report an adequate search strategy;
– Perform synthesis of the included studies;
– Assess the quality of the included studies;
– Provide sufficient details about the individual included studies.

We excluded the following study designs:

– Reviews which were not undertaken systematically;
– Narrative summaries of literature base;
– Primary studies;
– Qualitative evidence syntheses;
– Scoping and mapping reviews.

#### 4.2.2 Types of intervention/problem

We included OH interventions which met the following criteria:

– Multi‐disciplinary services designed to support employees and employers to manage health conditions in the workplace, to help employees with health conditions retain work and/or return to work following medical absence;
– Such interventions may be called OH, Vocational Rehabilitation (VR), Return to Work planning, as well as other labels (see Supporting Information Appendix 1 for complete list of terms used);
– By multi‐disciplinary, we mean that interventions must be delivered by more than one individual from different disciplines across both clinical and non‐clinical backgrounds. Acceptable combinations include:
  ∘ Clinical and non‐clinical professionals (e.g., psychiatrist and case‐manager);
  ∘ A mix of clinical professionals (e.g., psychiatrist and oncologist);
  ∘ A mix of non‐clinical professionals (e.g., social worker and case manager).
– Interventions delivered by public or private companies.

*We excluded the following types of intervention*:

– Services or interventions delivered by just one type of profession, whether clinical or non‐clinical;
– Services or interventions not delivered by or in association with the workplace;
– Interventions aiming to support unemployed people to get into work;
– Single component interventions that only involve the provision of equipment or environmental modifications;
– Interventions aiming to prevent poor health/promote good health.

Interventions compared with any comparator were included.

#### 4.2.3 Types of population

The focus of this EGM was on people aged 16 or above, who:

– Were in employment,
– Had an absence from work for any medical reason;
– Were in direct receipt of interventions for their own health;
– Were in direct receipt of workplace or job role interventions to enhance their return to work.

We excluded populations that included:

– Children aged below 16;
– Those who were unemployed;
– Parents/carers of people with relevant health conditions, but who themselves were not receiving an intervention for their health condition.

#### 4.2.4 Types of outcome measures

Systematic reviews were eligible for inclusion if work‐related outcomes were measured. These encompassed direct measures of RTW, work retention, measures of absence and any economic evaluation outcomes.

#### 4.2.5 Other eligibility criteria

##### Types of location

Systematic reviews could include studies from any country.

##### Types of settings

Interventions could be delivered within the workplace setting, or in other settings such as the community, primary or secondary care as long as there was some element of the intervention linked to the workplace.

### 4.3 Search methods and sources

Studies were identified according to the process described in our protocol (Shaw et al., 2022).

The search for relevant systematic reviews combined searches of bibliographic databases with searches of web‐based search engines and topically relevant websites. We also checked the reference lists of systematic reviews where the stated aim and characteristics of the population, interventions and outcomes measured, as stated within the review inclusion criteria, aligned with our review question.

The bibliographic database search strategies were developed using MEDLINE (via Ovid) by an information specialist (SB) in consultation with the review team and key stakeholders. The initial selection of search terms was derived from evidence on how to search for RTW studies (Gehanno et al., 2009), and the titles, abstracts and indexing terms of pre‐identified studies relevant to our research objectives. Search terms identified were therefore supplemented by an appropriate selection of synonyms and reviewed by stakeholders with expertise of returning to work following illness or parental leave. The experts included representatives from the DHSC and DWP and individuals with experience of accessing OH services, who were able to provide feedback on the appropriateness of the search terms and suggest additional terms for consideration.

The final search strategy included search terms that describe returning to work, such as ‘return to work’, ‘re‐entering work’ and ‘vocational rehabilitation’, and search terms which describe sickness absence (see Supporting Information Appendix 1), combined with a systematic review study type filter. We used controlled headings wherever they were available (e.g., MeSH in MEDLINE) alongside free‐text searching in the title and abstract fields of bibliographic records. Searches were conducted 28 June 2021 and updated on 21 February 2023. An historical date limit of 2001 was applied, following consultation with stakeholders, due to it offering the opportunity to capture evidence relevant to the current structure of OH services and the needs of the population they serve. The results were limited to English language studies. This was due to the large amount of literature available in this area identified during scoping, the broad scope of this review, policy timeline and limited resources available to support the translation of non‐English studies, which meant that inclusion of non‐English studies was not feasible for this review within the timeframe available.

We searched a selection of healthcare and non‐healthcare bibliographic databases to identify evidence from a variety of sectors of employment. The bibliographic databases are listed below, alphabetically ordered by provider:

– Campbell Collaboration (via https://www.campbellcollaboration.org/better-evidence)
– Cochrane Database of Systematic Reviews (via the Cochrane Library)
– Business Source Complete (via EBSCO)
– CINAHL (via EBSCO)
– EconLit (via EBSCO)
– Epistemonikos (via https://www.epistemonikos.org/en/)
– Health Management Information Consortium (HMIC) (via Ovid)
– MEDLINE ALL (via Ovid)
– Web of Science Core Collection (via Web of Science, Clarivate Analytics) including:
  ∘ Science Citation Index
  ∘ Social Science Citation Index
  ∘ Conference Proceedings—Science and Social Sciences

The Ovid MEDLINE search strategy is reproduced in Box 1. A full report of the bibliographic database search strategies is available in Supporting Information Appendix 1. The results of the bibliographic database searches were exported to Endnote X8 (Clarivate Analytics) and de‐duplicated using the automated de‐duplication feature and manual checking.

Box 1 **Ovid MEDLINE search strategy**

Issue: 1946 to June 25, 2021

Date Searched: 28/6/2021

Searcher: SB

Hits: 1125

Strategy:

1. (return* adj3 work*). tw.
2. ‘back to work’. tw.
3. (return* adj3 (occupation* or employ*)). tw.
4. Return to Work/
5. ((reentry or re entry or reenter* or ‘re enter*’) adj3 work*). tw.
6. ((reentry or re entry or reenter* or ‘re enter*’) adj3 (occupation* or employ*)). tw.
7. ((barrier* or facilitator*) adj2 (employ* or occupation* or work*)). tw.
8. ‘vocational rehabilitation’. tw.
9. ‘work rehabilitation’. tw.
10. ‘occupational rehabilitation’. tw.
11. Rehabilitation, Vocational/
12. ‘disability management‘. tw
13. or/1‐12
14. (sick* adj2 (leave or absence)). tw.
15. ‘case management’. tw
16. Sick Leave/
17. or/14‐16
18. (occupational adj2 (health or medicine or therap*)). tw.
19. Occupational Health/
20. Occupational Medicine/
21. Occupational Therapy/
22. or/18‐21
23. 17 and 22
24. 13 or 23
25. ((cochrane or cost or effectiveness or implementation or rapid or systematic or ‘state of the art’ or umbrella) adj2 (overview* or review* or synthes*)). tw.
26. (‘meta analy*’ or metaanaly* or metasynthe* or ‘meta synthe*’).tw.
27. ‘review* of reviews’. tw.
28. systematic review. pt.
29. meta‐analysis. pt.
30. or/25‐29
31. 24 and 30

Notes: date limited 2001 to date of search

To identify grey literature and studies not accessible via bibliographic databases we also searched Google Search (www.google.co.uk), Google Scholar (https://scholar.google.co.uk/) and a selection of topically relevant websites including:

– Health and Safety Executive (HSE) https://www.hse.gov.uk/
– HSE Solutions https://www.hsl.gov.uk/
– NHS Health at Work Network https://www.nhshealthatwork.co.uk/
– Society of Occupational Medicine https://www.som.org.uk/
– Faculty of Occupational Health Nursing https://www.fohn.org.uk/
– Council for Work and Health https://www.councilforworkandhealth.org.uk/

The search strategies used for Google Search, Google Scholar and websites are available in Supporting Information Appendix 1. Finally, we also consulted with stakeholders to identify reports already known to them.

### 4.4 Data collection and analysis

#### 4.4.1 Screening and study selection

Records from the bibliographic database search results were imported into EndNote libraries for screening.

Four reviewers independently undertook an initial calibration exercise to check inclusion judgments and the clarity of our eligibility criteria (LS, HL, LS, SGS). In a deviation from our protocol, these reviewers worked in pairs, with each pair screening fifty titles and abstracts from the bibliographic database search results. Decisions were discussed within each reviewer pair to ensure consistent application of criteria. The inclusion criteria were then applied to the title and abstract of the remaining reviews independently by two reviewers (LS, HL, SGS), with disagreements resolved through discussion or referral to a third reviewer as required. The full text of each record was screened for inclusion in the same way.

**Figure 1:**
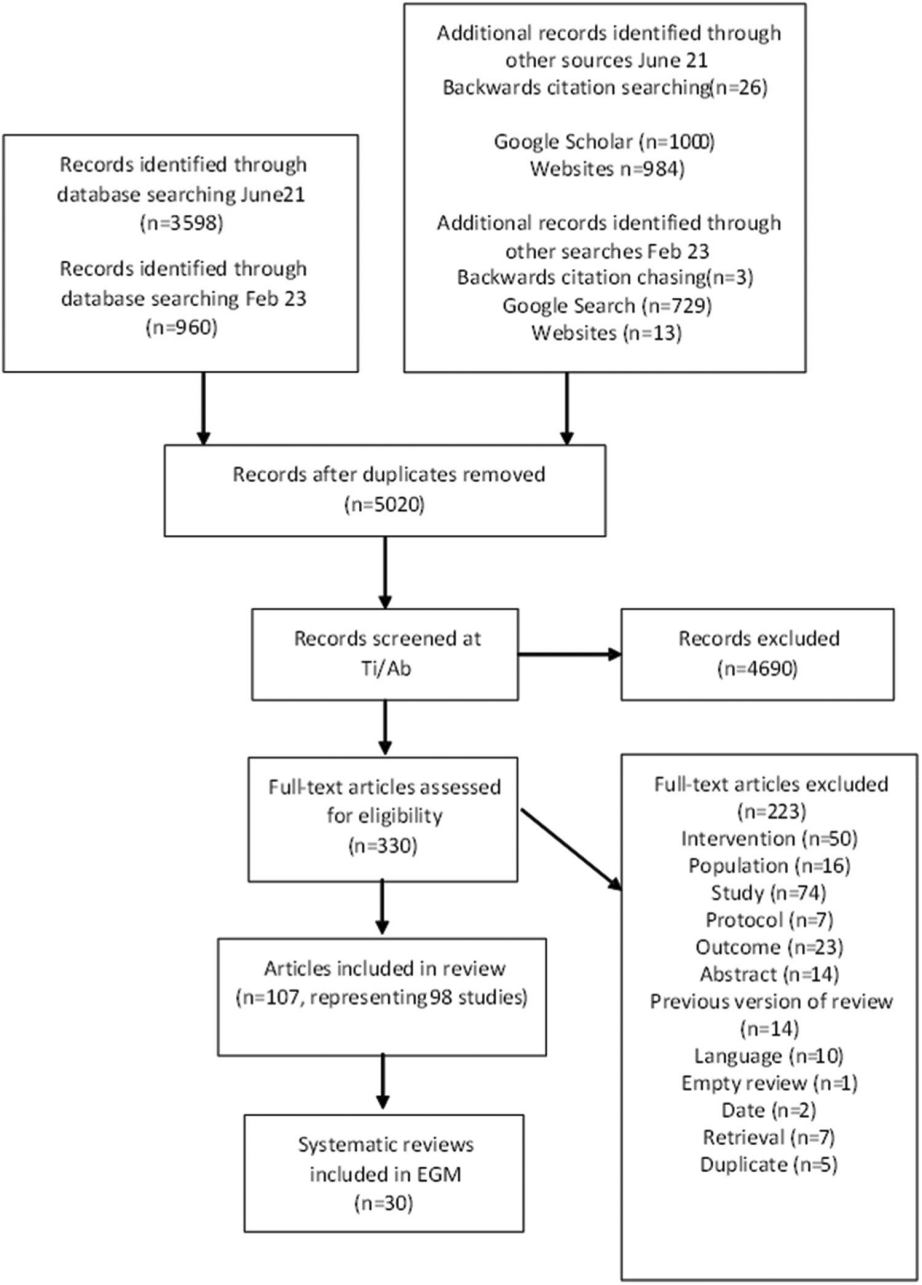
PRISMA diagram showing study selection process.

Endnote X8 software was used to support study selection and a PRISMA‐style flowchart (Figure 1: PRISMA diagram showing study selection process for systematic reviews with a return to work outcome) detailing the study selection process and reason for exclusion of each record retrieved at full text is reported below (Liberati et al., 2009).

#### 4.4.2 Data extraction and management

Due to the high number of systematic reviews meeting our inclusion criteria, data extraction was conducted in two stages. First, summary data for each eligible review were extracted by one reviewer and checked by a second using Microsoft Excel (LS, SGS, HL, MN). The summary data extracted from each included review are detailed in Supporting Information Appendix 2: Summary data extracted from all eligible reviews.

We used the summary information to categorise our included systematic reviews as being of high medium or low relevance to the research questions posed based on the following criteria:

- **High**: Aim of systematic review directly relevant to our research question, with potentially just one uncertainty around population (i.e., were they employed) or intervention (i.e., was it delivered by a MDT and in conjunction with the workplace?);
- **Medium**: Two uncertainties and/or aim of study not completely compatible with the aims of our review;
- **Low**: Two to three uncertainties regarding review inclusion criteria and limited quantity of relevant included primary studies, or limited quantity number of included primary studies relevant to aim of review alone.

Judgements on relevance were made independently by two reviewers; any disagreements discussed with the wider team to achieve consensus. In the second stage of data extraction, we developed a standardised data extraction form which was piloted by two reviewers (LS, MN) on a selection (*n* = 5) of included studies. The data extraction form was amended following this, to account for revised Quality Appraisal criteria (as described below) and add further detail regarding the country the review was conducted in versus the countries eligible studies were conducted in as specified by the review inclusion criteria. This revised data extraction form was used to support the data extraction of the remaining high/medium relevance systematic reviews. The following information was extracted from each systematic review:

– Age of sample as cited in inclusion criteria;
– Country review conducted in;
– Country included primary studies conducted in (as reported in inclusion criteria);
– Health conditions of sample as cited in inclusion criteria;
– Intervention of interest;
– Area of work/sector/employer;
– Whether review inclusion criteria and/or synthesis strategy considered any of the PROGRESS criteria (place of residence, race/ethnicity/culture/language, gender/sex, religion, education, socio‐economic status, social capital) (Welch et al., 2019);
– Number of primary studies with findings relevant to focus of this EGM and summary of their findings with respect to RTW outcomes;
– RTW outcome main findings.

Data extraction was performed by one reviewer (MN, JTC) and checked by a second (LS), with disagreements being settled through discussion. EPPI‐Reviewer software was used to support data extraction (Thomas et al., 2010).

#### 4.4.3 Assessing the Methodological Quality of Systematic Reviews (AMSTAR 2)

The quality of the systematic reviews rated as ‘High’ or ‘Medium’ relevance following full text screening was appraised using the AMSTAR‐2 quality appraisal tool for systematic reviews of primary studies of randomised and non‐randomised study designs within EPPI‐Reviewer (Supporting Information Appendix 3). (Shea et al., 2017; Thomas et al., 2010) Quality appraisal was undertaken by one reviewer (MN, JTC) and checked by a second (LS), with disagreements being resolved through discussion.

Reviews were rated as High, Moderate, Low and Critically‐Low quality, with ratings determined by the following system:

- High: No or one non‐critical weakness: the systematic review provides an accurate and comprehensive summary of the results of the available studies that address the question of interest;
- Moderate: More than one non‐critical weakness. The systematic review has more than one weakness but no critical flaws;
- Low: One critical flaw with or without non‐critical weaknesses: the review has a critical flaw and may not provide an accurate and comprehensive summary of the available studies that address the question of interest;
- Critically‐Low: More than one critical flaw with or without non‐critical weaknesses: the review has more than one critical flaw and should not be relied on to provide an accurate and comprehensive summary of the available studies.

We considered items 2, 4, 9, 11 and 13 of the AMSTAR‐2 tool as ‘critical domains’ in judging review quality.

#### 4.4.4 Methods for mapping

The data extracted from systematic reviews of ‘High’ and ‘Medium’ relevance using EPPI‐Reviewer 4 was then imported into EPPI‐Mapper software to create an EGM (Thomas et al., 2010).

### 4.5 Framework development and scope

The scope of this EGM was to capture systematic review evidence on the effectiveness and cost‐effectiveness of workplace‐based, multi‐disciplinary OH interventions. We sought evidence published from 2001 onwards to identify evidence most relevant to our stakeholders.

The main axis of the EGM is structured according to the health condition that led to sick leave, and the main findings relating to the work‐related outcome(s) reported at review level. A list of common health conditions to include in the map was generated by reviewers, drawing upon our knowledge of the characteristics of studies found through our scoping process and title and abstract screening and discussion with stakeholders. We consulted with our stakeholders to ensure the structure, content and description of the map were accessible and met their requirements.

The segmenting filter for the map was the overall quality rating awarded to each review, based on the studies’ overall score on the AMSTAR‐2 tool. Each review was given a ‘High’, ‘Moderate’, ‘Low’ or ‘Critically low’ based on the number of methodological weaknesses within several critical domains (as described within ‘Tools for assessing risk of bias’ section below) and represented by different colour circles indicating both the quantity and quality of the evidence within each cell.

### 4.6 Description of health condition

Within the EGM, columns are separated according to the different types of health conditions that resulted in study participants taking sick leave as specified in the inclusion criteria of reviews prioritised for inclusion in the map. Categories of health conditions were as follows: anxiety, arthritis, cancer, chronic pain, depression, dermatological issues, multiple sclerosis, stress/burnout, musculoskeletal, stroke, traumatic brain injury, traumatic physical injury and ‘other’. If a review includes workers with different health conditions, it appears in multiple cells within the map.

### 4.7 Description of outcomes

Work‐related outcomes (defined within the ‘Types of Outcomes measured’ above) as reported within the systematic reviews, were included in the map; we grouped these into four categories: Reviews reporting (a) Positive findings: OH interventions being evaluated were found to have a significantly beneficial effect on work‐related outcomes, (b) Mixed‐findings: OH interventions being evaluated had a mixed‐effect on work‐related outcomes, (c) Inconclusive/weak evidence: interventions being evaluated were reported as having an unclear impact on work‐related outcomes or analysis were methodologically weak (i.e., the number or quality of trials included in the analysis was insufficient and thus reduced confidence in reported outcome), (d) No effect: OH intervention was reported to have no significant effect on work‐related outcomes.

The comments section of the abstract for each review also provides links to the included primary studies relevant to the overall aims of our EGM, grouped according to the direction of work‐related outcome results.

#### 4.7.1 Filters for presentation

The content of the map can be changed using the ‘Filters’ option at the top right‐hand side of the map, according to different features of the systematic reviews. Different filter options were as follows:

1. **Population age**: The minimum age of participants specified within the inclusion criteria of each included systematic review. Categories included people aged 16 and above, 17 and above, 18 and above, 50 and above, 65 and above, older adults unspecified, adults unspecified and ‘other’;
2. **If review criteria considered any of the following PROGRESS criteria**: Place of residence, race/ethnicity/culture/language, gender/sex, religion, education, socio‐economic status, social capital;
3. **If review synthesis strategy considered any of the PROGRESS criteria**: As listed above;
4. **Intervention category**: We categorised systematic reviews by the type of interventions they sought and evaluated, using the following four categories:
  a) Broad (review sought a variety of interventions based on changes at staff, programme and/or workplace level);
  b) Specific—Staff (review sought interventions where staff was the focus, regardless of the package being delivered or the setting, e.g., the introduction of RTW coordinators or interventions involving psychiatrists only);
  c) Specific—programme (review focused on certain types of intervention, regardless of staff involved or the setting e.g. rehabilitation programmes);
  d) Specific—setting (review was interested in interventions delivered in a certain setting, regardless of the staff involved or the type of intervention, e.g., onsite RTW programmes).

### 4.8 Analysis and presentation

Each segment of the EGM can be clicked upon to view the abstracts of the systematic reviews included in that segment, containing details of the background, methods, results, main findings of the systematic review and links to the systematic review full text. The abstract for each review provides a link to each primary study included within it which is relevant to our research question, alongside a summary of its main findings with respect to RTW outcomes.

The ‘About’ section at the top of the map describes the context and aim of the EGM and provides an explanation to help users navigate the map. Each segment of the map indicates the number of reviews relevant to these intersecting categories, grouped according to the quality of the review (Green: High quality, Yellow = Moderate quality, Orange = Low quality, Red = Critically Low quality). Thus, the size and colour of the circles within each segment represent the number and quality of reviews reporting RTW outcomes for interventions conducted with particular health conditions.

## 5 RESULTS

### 5.1 Description of studies

#### 5.1.1 Results of the search

Figure 1 provides an overview of the search and screening process for this EGM. The bibliographic database searches identified 4558 records. A further 2755 records were identified via alternative search methods, including backwards citation chasing (*n* = 29), website searches (*n* = 997), Google Scholar (*n* = 1000) and Google (*n* = 729). Following de‐duplication, there were 5020 unique records. At title and abstract screening, 4690 records were excluded leaving 330 studies to screen at full text. Of these 223 were excluded for the reasons listed in Figure 1. Ninety‐eight systematic reviews (107 articles) met our eligibility criteria for inclusion in this review. Summary data for all eligible systematic reviews can be found in the Supporting Information.

#### 5.1.2 Excluded studies

Studies excluded after screening at full text, along with reasons for exclusion, can be found in the reference section at the end of this report: List of ‘Excluded studies’. The most common reasons for exclusion were study design or type of intervention.

### 5.2 Synthesis of included studies

Twenty‐six of the 98 systematic reviews included in this EGM review were rated as being of ‘High’ relevance, 8 as ‘Medium’ relevance and 64 as ‘Low’ relevance based upon the extent to which the aims/inclusion criteria of these reviews were consistent with the aims and objectives of our EGM. Two of the systematic reviews rated as being of ‘High’ relevance and two rated as being of ‘Medium’ relevance were systematic reviews of reviews. Three of these included systematic reviews which duplicated the systematic reviews identified through other methods (Snodgrass, 2011; Vooijs et al., 2015; White et al., 2016), and one (Vandenbroeck et al., 2016) contained data where it was difficult to determine the relevance. The 30 systematic reviews rated as ‘High’ and ‘Medium’ relevance were prioritised for full data extraction and inclusion in the EGM.

**Figure 2:**
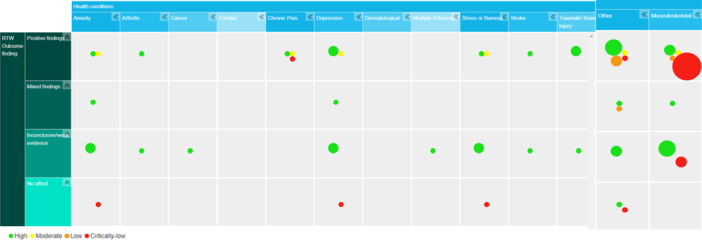
Evidence and gap map of included studies, showing intervention and outcome categories (sub‐categories can be accessed in the interactive map) and study quality/risk of bias (blue indicates higher quality and orange lower quality).

The studies included in the online EGM can be viewed in Supporting Information Appendix 4 and a link to the EGM is provided in Supporting Information Appendix 5. Figure 2 illustrates the main features of the map, with the intervention outcome and condition categories displayed at the sides of the map, with circle size and colour representing the quantity and quality of evidence within each cell. The main characteristics of the reviews included in the EGM are described below.

### 5.3 Publication date and distribution by location

Table 2 contains details of the 30 included systematic reviews of primary studies rated as being of ‘High’ or ‘Medium’ relevance to our aims and objectives. The earliest of the reviews was published in 2005 (Franche et al., 2005), with 17 published since 2016 (Axen et al., 2020; Bernaers et al., 2022; Cochrane et al., 2017; Cullen et al., 2018; Gaillard et al., 2020; Heathcote et al., 2019; Ishimaru et al., 2021; Kojimahara et al., 2020; Lefever et al., 2018; Mikkelsen & Rosholm, 2018; NICE, 2019; Nieuwenhuijsen et al., 2020; Oakman et al., 2016; Tingulstad et al., 2022; Venning et al., 2021; Verhoef et al., 2020; Vogel et al., 2017). Reviews were conducted by teams from 10 different countries, with six publications coming from The Netherlands (Hoefsmit et al., 2012; Nieuwenhuijsen et al., 2020; Schaafsma et al., 2013; van Geen et al., 2007; van Vilsteren et al., 2015; Verhoef et al., 2020), five from Canada (Brewer et al., 2007; Cullen et al., 2018; Franche et al., 2005; Gaillard et al., 2020; Tompa et al., 2008), three from the UK (Carroll et al., 2010; NICE, 2019; Palmer et al., 2012), Australia (Heathcote et al., 2019; Oakman et al., 2016; Venning et al., 2021) and Norway (Neverdal, 2015; Odeen et al., 2013; Tingulstad et al., 2022), two from Denmark (Gensby et al., 2014; Mikkelsen & Rosholm, 2018), Japan,(Ishimaru et al., 2021; Kojimahara et al., 2020) and Belgium (Bernaers et al., 2022; Lefever et al., 2018), and one each from Sweden (Axén et al., 2020), Ireland (Cochrane et al., 2017), Switzerland (Verhoef et al., 2020) and between Canada and Switzerland (Schandelmaier et al., 2012). Regarding geographical restrictions imposed as part of the inclusion criteria in included reviews, only two studies enforced any (Oakman et al., 2016; Venning et al., 2021). Oakman et al. (2016) required studies to be conducted in countries with disability support schemes that provide support for individuals regardless of cause, or, for countries with cause‐based systems, where the primary reason for work absence was considered a workplace injury or illness, and participants were receiving support through a cause‐based workers’ compensation system. The study conducted by Venning et al. (2021) required grey literature studies to be authored by an Australian RTW organisation (Venning et al., 2021).

**Table 2:** Characteristics of 30 systematic reviews prioritised for inclusion in EGM.

First author (year) [country where conducted]	Age	Health conditions	Intervention category	Area of work/sector/employer	Quality Rating	Number of relevant includes (articles/studies)	RTW outcome finding
Gaillard (2020) (Gaillard et al., 2020) [Canada]	Other (working age adults)	Anxiety, depression, common mental disorders	Specific—programme: work related factors	NR	High	5/5	Positive effect
Gensby (2012) (Gensby et al., 2014) [Denmark]	Adults unspecified	Anxiety, arthritis, cancer, depression, multiple sclerosis, stress or burnout, stroke, traumatic brain injury, traumatic physical injury, musculoskeletal, other (neurological illness, fatigue, somatic illness, eye strain)	Specific—setting	NR	High	6/4	Inconclusive/weak evidence
Heathcote (2019) (Heathcote et al., 2019) [Australia]	18‐70	Traumatic brain injury, traumatic physical injury, musculoskeletal	Specific—programme: worker resilience	NR	High	4/4	Positive effect
NICE (2019) (NICE, 2019) [UK]	16+	Anxiety, depression, stress or burnout, musculoskeletal, anything causing long term sickness absence	Broad	NR	High	20/20	Inconclusive/weak evidence
Nieuwenhuijsen (2020) (Nieuwenhuijsen et al., 2020) [Netherlands]	17+	Depression	Broad	Any	High	6/6	Positive effect
Schaafsma (2013) (Schaafsma et al., 2013) [Netherlands]	16+	Musculoskeletal	Specific—programme: physical conditioning	NR	High	12/10	Inconclusive/weak evidence
Schandelmaier (2012) (Schandelmaier et al., 2012) [Switzerland, Canada]	16‐65	Other (any recorded disability status)	Specific—staff: involve RTW co‐ordinator	NR	High	3/3	Positive effect
van Vilsteren (2015) (van Vilsteren et al., 2015) [Netherlands]	18‐65	Anxiety, depression, musculoskeletal, other (mental health problems, other health conditions)	Specific—setting	NR	High	12/12	Mixed effect
Verhoef (2020) [Netherlands]	18‐65	Arthritis, chronic pain, stress or burnout, stroke, musculoskeletal, traumatic brain injury, other (chronic physical or somatic diseases, HIV/AIDS, spinal cord injury)	Broad	NR	High	6/6	Positive effect
Vogel (2017) (Vogel et al., 2017) [Switzerland]	16‐65	Other (not stated)	Specific—programme: RTW co‐ordination	NR	High	7/7	No effect
Bernaers (2022) (Bernaers et al., 2022) [Belgium]	18+	Musculoskeletal	Broad	NR	Moderate	7/12	Mixed effect
Cochrane (2017) (Cochrane et al., 2017) [Ireland]	18+	Chronic pain, musculoskeletal. Excluded inflammatory conditions	Specific—programme: biopsychosocial	NR	Moderate	9/9	Positive effect
Mikkelsen (2018) (Mikkelsen & Rosholm, 2018) [Denmark]	Adults unspecified	Anxiety, depression, stress or burnout, other (adjustment disorders, personality disorders, somatoform disorders)	Broad	NR	Moderate	12/12	Positive effect
Tingulstad (2022) (Tingulstad et al., 2022) [Norway]	Adults unspecified	Depression, musculoskeletal, stress/burnout, other (CMD, adjustment and somatic disorders)	Specific—setting	NR	Moderate	18/20	Inconclusive/weak evidence
Tompa (2007/2008) (Tompa et al., 2008) [Canada]	Adults unspecified	Other (not stated)	Broad	NR	Moderate	11/8	Positive effect
Brewer (2007) (Brewer et al., 2007) [Canada]	18+	Musculoskeletal, work‐related injuries and illnesses	Specific—programme: injury prevention/loss control	Multiple, except agricultural workers, migrant workers, tele‐workers, home offices/workers, military installations, commercial fishing	Low	6/6	Positive effect
Cullen (2018) (Cullen et al., 2018) [Canada]	Adults unspecified	Depression, musculoskeletal	Specific—setting	Public administration (*n* = 2), professional, scientific or technical services (*n* = 3), mining (*n* = 1), construction (*n* = 2), agriculture (*n* = 2), manufacturing (*n* = 10), transportation (*n* = 3), health care and social assistance (*n* = 17), educational services (*n* = 3), hospitality and other services (*n* = 5), other (*n* = 5), unknown (*n* = 13). Some studies included populations from multiple sectors	Low	18/36	Mixed effect
Lefever (2018) (Lefever et al., 2018) [Belgium]	NR	Other (all disabilities)	Specific—programme: biopsychosocial DMP	NR	Low	4/4	Positive effect
Odeen (2013) (Odeen et al., 2013) [Norway]	18+	Other (not stated)	Broad	NR	Low	5/5	Mixed effect
Axen (2020) [Sweden]	Adults unspecified	Anxiety, depression, stress or burnout, other (common mental disorders, incorporating depression, anxiety, adjustment disorders, insomnia and stress‐related ill health)	Specific—staff: involve OH services	NR	Critically Low	9/7	No effect
Carroll et al. (2010) (Carroll et al., 2010) [UK]	Adults unspecified	Musculoskeletal	Specific—setting	NR	Critically Low	8/8	Positive effect
Franche (2005) (Franche et al., 2005) [Canada]	Adults unspecified	Chronic pain, musculoskeletal	Broad	NR	Critically Low	6/5	Positive effect
Hoefsmit (2012) (Hoefsmit et al., 2012) [Netherlands]	Adults unspecified	Anxiety, depression, stress/burnout, musculoskeletal, other (sickness absence, adjustment and somatoform disorders)	Broad	NR	Critically Low	7/23	Mixed effect
Ishimaru (2021) (Ishimaru et al., 2021) [Japan]	Adults unspecified	Musculoskeletal	Specific—programme	NR	Critically Low	7/9	Inconclusive/weak evidence
Kojimahara (2020) (Kojimahara et al., 2020) [Japan]	NR	Musculoskeletal, mental health disorders	Broad	NR	Critically Low	9/9	Positive effect
Neverdal (2015) (Neverdal, 2015) [Norway]	Adults unspecified	Musculoskeletal	Specific—setting	NR	Critically Low	7/7	Positive effect
Oakman (2016) (Oakman et al., 2016) [Australia]	Adults unspecified	Musculoskeletal	Broad	NR	Critically Low	7/6	Inconclusive/weak evidence
Palmer (2012) (Palmer et al., 2012) [UK]	Other (working age adults)	Musculoskeletal	Broad	NR	Critically Low	19/14	Inconclusive/weak evidence
van Geen (2007) (van Geen et al., 2007) [Netherlands]	18‐65	Musculoskeletal	Specific—programme: MDT back training	NR	Critically Low	1/1	Positive effect
Venning (2021) (Venning et al., 2021) [Australia]	18+	Cardiac, chronic pain, depression, musculoskeletal, stress/burnout, other (adjustment disorder, psychological problems, disc disease or spondylolisthesis receiving lumbar spinal fusion surgery, hysterectomy and/or laparoscopic adnexal surgery)	Specific—programme	NR	Critically Low	7/36	No effect

Abbreviations: MDT, multi‐disciplinary Team; NR, not reported; OH, occupational health; RTW, return to work.

### 5.4 Study populations

All 30 reviews were concerned with adults of working age, with this stipulated to be from as young as 16 years old (NICE, 2019; Schaafsma et al., 2013; Schandelmaier et al., 2012) up to 70 years old (Heathcote et al., 2019). Of the health conditions studied, 12 cast a wide net, seeking studies of participants with a wide range of conditions (Gensby et al., 2014; Hoefsmit et al., 2012; Lefever et al., 2018; NICE, 2019; Odeen et al., 2013; Schandelmaier et al., 2012; Tingulstad et al., 2022; Tompa et al., 2008; van Vilsteren et al., 2015; Venning et al., 2021; Vogel et al., 2017). Of those that were more focused, there were 11 reviews with a focus on workers with musculoskeletal conditions and/or chronic pain (Bernaers et al., 2022; Brewer et al., 2007; Carroll et al., 2010; Cochrane et al., 2017; Franche et al., 2005; Ishimaru et al., 2021; Neverdal, 2015; Oakman et al., 2016; Palmer et al., 2012; Schaafsma et al., 2013; van Geen et al., 2007), three that looked exclusively at mental health conditions (Gaillard et al., 2020; Mikkelsen & Rosholm, 2018; Nieuwenhuijsen et al., 2020), and one which included participants with musculoskeletal and/or mental health conditions (Cullen et al., 2018). There was almost no information provided about the industry or work sector in which the primary studies had been conducted, with only Brewer and colleagues mentioning some exclusions (Brewer et al., 2007). It was assumed that any industry or workplace would be of interest in the remaining reviews.

The systematic review conducted by NICE (NICE, 2019) considered race/ethnicity/culture/language, gender/sex, and socio‐economic status in their synthesis; Nieuwenhuijsen and colleagues (Nieuwenhuijsen et al., 2020) considered the influence of gender/sex in their synthesis; Schaafsma and colleagues had inclusion criteria relating to gender/sex (Schaafsma et al., 2013), and Venning et al for place of residence (Venning et al., 2021). Aside from these four reviews, the PROGRESS criteria did not appear in the inclusion criteria or synthesis strategy for any review (Welch et al., 2019).

### 5.5 Description of interventions

Interventions were categorised as staff‐specific in two reviews (Axén et al., 2020; Schandelmaier et al., 2012). In the paper by Axén and colleagues (2020) (Axén et al., 2020), there was a specific requirement for interventions to involve OH services staff, while Schandelmaier et al. (2012) required interventions to primarily involve a return‐to‐work coordinator (Schandelmaier et al., 2012).

Eight reviews sought specific types of intervention (Brewer et al., 2007; Cochrane et al., 2017; Gaillard et al., 2020; Heathcote et al., 2019; Lefever et al., 2018; Schaafsma et al., 2013; van Geen et al., 2007; Vogel et al., 2017). Brewer and colleagues sought injury prevention and loss control programmes (policies, procedures and practices to protect workers, meet regulatory requirements, reduce adverse consequences of worker injuries, and manage costs) (Brewer et al., 2007); Cochrane and colleagues were interested in any biopsychosocial interventions (Cochrane et al., 2017); Gaillard et al. sought interventions aiming to change work‐related factors (Gaillard et al., 2020); Heathcote and colleagues looked for any intervention targeting worker resilience (Heathcote et al., 2019); Lefever and colleagues sought biopsychosocial disability management programmes (Lefever et al., 2018); Schaafsma et al included physical conditioning programmes (Schaafsma et al., 2013); van Geen et al were interested in multidisciplinary back training programmes (based on bio‐psycho‐social principles to support patients manage their lower back pain) (van Geen et al., 2007); and Vogel and colleagues included any return‐to‐work coordination programmes (Vogel et al., 2017).

### 5.6 Risk of bias in included reviews

Table 3 provides a breakdown of AMSTAR‐2 ratings for each of the 30 reviews included in the EGM. Scores are provided for each item on the AMSTAR‐2 checklist, alongside an overall rating. Of the 30 systematic reviews, 10 were allocated a rating of ‘High’ quality (Gaillard et al., 2020; Gensby et al., 2014; Heathcote et al., 2019; NICE, 2019; Nieuwenhuijsen et al., 2020; Schaafsma et al., 2013; Schandelmaier et al., 2012; van Vilsteren et al., 2015; Verhoef et al., 2020; Vogel et al., 2017), four of ‘Moderate’ quality (Bernaers et al., 2022; Cochrane et al., 2017; Mikkelsen & Rosholm, 2018; Tingulstad et al., 2022), four of ‘Low’ quality (Cullen et al., 2018; Lefever et al., 2018; Odeen et al., 2013; Tompa et al., 2008) and 12 of ‘Critically Low’ quality (Axén et al., 2020; Brewer et al., 2007; Carroll et al., 2010; Franche et al., 2005; Hoefsmit et al., 2012; Ishimaru et al., 2021; Kojimahara et al., 2020; Neverdal, 2015; Oakman et al., 2016; Palmer et al., 2012; van Geen et al., 2007; Venning et al., 2021).

**Table 3:** Risk of bias of studies included within evidence and gap map.

Study	1. PICO components	2. Protocol	3. Study design explanation	4. Comprehensive search strategy	5. Duplicate study selection	6. Duplicate data extraction	7. Details of excluded studies	8. Description of included studies	9a. Risk of Bias (RoB) assessment (RCTs)
Axen (2020); Axén et al. 2020)	Yes	No	No	Yes	Yes	No	No	Yes	No
Bernaers (2022); Bernaers et al. (2022)	Yes	Yes	No	PY	No	No	Yes	PY	Yes
Brewer (2007); Brewer et al. (2007)	Yes	No	No	Yes	No	Yes	No	Yes	No
Carroll et al. (2010); Carroll et al. (2010)	Yes	No	No	Yes	No	Yes	Yes	Yes	Yes
Cochrane (2017); Cochrane et al. (2017)	Yes	Yes	No	Yes	Yes	Yes	Yes	Yes	Yes
Cullen (2018); Cullen et al. (2018)	Yes	No	No	PY	No	Yes	No	PY	Yes
NICE (2019); NICE (2019)	Yes	Yes	No	Yes	Yes	Yes	Yes	Yes	Yes
Franche (2005); Franche et al. (2005)	Yes	No	No	No	Yes	Yes	No	Yes	No
Gaillard (2020); Gaillard et al. (2020)	No	Yes	Yes	Yes	Yes	Yes	Yes	Yes	NA
Gensby (2012); Gensby et al. (2014)	Yes	Yes	Yes	Yes	Yes	Yes	Yes	Yes	Yes
Heathcote (2019); Heathcote et al. (2019)	Yes	Yes	No	Yes	Yes	Yes	No	Yes	Yes
Hoefsmit (2012); Hoefsmit et al. (2012)	No	No	Yes	No	No	No	No	No	Yes
Ishimaru (2021); Ishimaru et al. (2021)	No	No	No	No	Yes	No	No	Yes	Yes
Kojimahara (2020); Kojimahara et al. (2020)	Yes	Yes	No	Yes	No	No	No	Yes	Yes
Lefever (2018); Lefever et al. (2018)	Yes	Yes	No	Yes	Yes	Yes	No	Yes	Yes
Mikkelsen (2018); Mikkelsen & Rosholm, (2018)	Yes	Yes	No	Yes	Yes	Yes	No	Yes	Yes
Neverdal (2015); Neverdal (2015)	Yes	No	No	No	No	No	No	Yes	Yes
Nieuwenhuijsen (2020); Nieuwenhuijsen et al. (2020)	Yes	Yes	No	Yes	Yes	Yes	Yes	Yes	Yes
Oakman (2016); Oakman et al. (2016)	Yes	No	No	No	Yes	No	No	No	Yes
Odeen (2013); Odeen et al. (2013)	Yes	Yes	No	Yes	Yes	Yes	Yes	Yes	Yes
Palmer (2012); Palmer et al. (2012)	Yes	No	No	Yes	No	Yes	No	Yes	Yes
Schaafsma (2013); Schaafsma et al. (2013)	Yes	Yes	No	Yes	Yes	Yes	Yes	Yes	Yes
Schandelmaier (2012); Schandelmaier et al. (2012)	Yes	Yes	No	Yes	Yes	Yes	No	Yes	Yes
Tingulstad (2022); Tingulstad et al. (2022)	Yes	Yes	No	Yes	Yes	Yes	Yes	PY	Yes
Tompa (2008); Tompa et al. (2008)	Yes	Yes	No	No	No	Yes	No	No	Yes
van Geen (2007); van Geen et al. (2007)	Yes	No	No	No	No	No	No	Yes	Yes
van Vilsteren (2015); van Vilsteren et al. (2015)	Yes	Yes	No	Yes	Yes	Yes	Yes	Yes	Yes
Venning (2021); Venning et al. (2021)	Yes	No	No	Yes	Yes	Yes	No	Yes	Yes
Verhoef (2020); Verhoef et al. (2020)	Yes	Yes	No	Yes	Yes	Yes	No	Yes	Yes
Vogel (2017); Vogel et al. (2017)	Yes	Yes	No	Yes	Yes	Yes	Yes	Yes	Yes

Study	9b. RoB assessment (NRSIs)	10. Funding sources	11a. RCTs Meta‐analysis	11b. NRSIs Meta‐analysis (MA)	12. MA: RoB in individual studies	13. RoB: discussion of results	14. Heterogeneity	15. Publication bias	16. Reports conflicts of interest	Overall rating
Axen (2020); Axén et al. (2020)	No	No	NA	NA	NA	No	No	NA	Yes	Critically low
Bernaers (2022); Bernaers et al. (2022)	Yes	No	NA	NA	NA	Yes	Yes	NA	Yes	Moderate
Brewer (2007); Brewer et al. (2007)	Yes	No	NA	NA	NA	Yes	Yes	NA	No	Critically low
Carroll et al. (2010); Carroll et al. (2010)	Yes	No	NA	NA	NA	Yes	Yes	NA	No	Critically low
Cochrane (2017); Cochrane et al. (2017)	NA	No	Yes	NA	No	Yes	Yes	No	Yes	Moderate
Cullen (2018); Cullen et al. (2018)	Yes	No	NA	NA	NA	Yes	No	NA	Yes	Critically low
NICE (2019); NICE (2019)	Yes	Yes	Yes	NA	Yes	Yes	Yes	No	Yes	High
Franche (2005); Franche et al. (2005)	No	No	NA	NA	NA	Yes	Yes	NA	No	Critically low
Gaillard (2020); Gaillard et al. (2020)	Yes	Yes	NA	NA	NA	Yes	Yes	NA	Yes	High
Gensby (2012); Gensby et al. (2014)	Yes	No	NA	NA	NA	Yes	Yes	NA	Yes	High
Heathcote (2019); Heathcote et al. (2019)	NA	No	Yes	NA	Yes	Yes	Yes	Yes	Yes	High
Hoefsmit (2012); Hoefsmit et al. (2012)	Yes	No	NA	NA	NA	Yes	Yes	NA	No	Critically low
Ishimaru (2021); Ishimaru et al. (2021)	NA	No	NA	NA	NA	No	No	NA	Yes	Critically low
Kojimahara (2020); Kojimahara et al. (2020)	Yes	No	Yes	NA	No	Yes	No	Yes	Yes	Critically low
Lefever (2018); Lefever et al. (2018)	Yes	No	NA	NA	NA	No	Yes	NA	Yes	Low
Mikkelsen (2018); Mikkelsen & Rosholm, (2018)	Yes	No	Yes	Yes	Yes	Yes	Yes	Yes	Yes	Moderate
Neverdal (2015); Neverdal (2015)	NA	No	NA	NA	NA	Yes	Yes	NA	No	Critically low
Nieuwenhuijsen (2020); Nieuwenhuijsen et al. (2020)	NA	Yes	Yes	NA	Yes	Yes	Yes	Yes	Yes	High
Oakman (2016); Oakman et al. (2016)	Yes	No	NA	NA	NA	Yes	Yes	NA	Yes	Critically low
Odeen (2013); Odeen et al. (2013)	NA	No	NA	NA	NA	Yes	Yes	NA	Yes	Low
Palmer (2012); Palmer et al. (2012)	Yes	No	NA	NA	NA	Yes	Yes	Yes	Yes	Critically low
Schaafsma (2013); Schaafsma et al. (2013)	NA	No	Yes	NA	Yes	Yes	Yes	Yes	Yes	High
Schandelmaier (2012); Schandelmaier et al. (2012)	NA	No	Yes	NA	Yes	Yes	Yes	No	Yes	High
Tingulstad (2022); Tingulstad et al. (2022)	NA	No	Yes	NA	Yes	Yes	Yes	No	Yes	Moderate
Tompa (2008); Tompa et al. (2008)	Yes	No	NA	NA	NA	Yes	Yes	NA	No	Low
van Geen (2007); van Geen et al. (2007)	NA	No	NA	NA	NA	Yes	Yes	NA	Yes	Critically low
van Vilsteren (2015); van Vilsteren et al. (2015)	NA	No	Yes	NA	Yes	Yes	Yes	Yes	Yes	High
Venning (2021); Venning et al. (2021)	NA	No	NA	NA	NA	No	Yes	NA	Yes	Critically low
Verhoef (2020); Verhoef et al. (2020)	NA	No	Yes	NA	Yes	Yes	Yes	Yes	Yes	High
Vogel (2017); Vogel et al.al. (2017)	NA	Yes	Yes	NA	Yes	Yes	Yes	Yes	Yes	High

Abbreviations: NRSI, non‐randomised studies of interventions; NA, not applicable; RCT, randomised controlled trial; y, yes=partial yes;

To be rated as ‘Critically Low’ quality, more than one critical flaw must be observed. Critical items were numbers 2, 4, 9, 11 and 13. By far the most commonly failed item was item 2, with 11 of the 12 ‘Critically Low’ rated reviews not having a protocol (Axén et al., 2020; Brewer et al., 2007; Carroll et al., 2010; Franche et al., 2005; Neverdal, 2015; Oakman et al., 2016; Palmer et al., 2012; van Geen et al., 2007) (Hoefsmit et al., 2012; Ishimaru et al., 2021; Venning et al., 2021).

Across the 30 reviews, only three provided a justification for the study designs they chose to include (Gaillard et al., 2020; Gensby et al., 2014; Hoefsmit et al., 2012), only four reported funding sources in their included studies (Gaillard et al., 2020; NICE, 2019; Nieuwenhuijsen et al., 2020; Vogel et al., 2017). It is also notable that there was no evidence of duplicate study selection being performed in ten studies (Bernaers et al., 2022; Brewer et al., 2007; Carroll et al., 2010; Cullen et al., 2018; Hoefsmit et al., 2012; Kojimahara et al., 2020; Neverdal, 2015; Palmer et al., 2012; Tompa et al., 2008; van Geen et al., 2007), or data extraction (*n* = 8 studies) (Axén et al., 2020; Bernaers et al., 2022; Hoefsmit et al., 2012; Ishimaru et al., 2021; Kojimahara et al., 2020; Neverdal, 2015; Oakman et al., 2016; van Geen et al., 2007).

### 5.7 Summary of main findings

In addition to possessing a variety of quality ratings and sizes, the reviews featured an array of health conditions and intervention types, and thus represent a highly heterogeneous body of evidence. This heterogeneity meant it was not possible to structure the map according to condition and types of intervention being evaluated. Instead, the map is structured by the reason for sick leave and reported impact on RTW outcomes as reported at the level of the review, with links to the primary studies which contain descriptions of individual interventions provided within each segment.

Figure 2 indicates that the highest quantity of systematic review evidence was for interventions targeting employees with musculoskeletal conditions. For interventions with individuals with musculoskeletal disorders, nine reviews reported a significant beneficial effect of the intervention. However, only two of these reviews were of ‘High’ quality (Heathcote et al., 2019; Verhoef et al., 2020), with one appraised as ‘Moderate’ quality (Cochrane et al., 2017), one as ‘Low’ quality (Brewer et al., 2007) and five as ‘Critically Low’ quality (Carroll et al., 2010; Franche et al., 2005; Kojimahara et al., 2020; Neverdal, 2015; van Geen et al., 2007). The next largest group of evidence was for reviews reporting inconclusive or weak evidence with respect to intervention effectiveness (*n* = 7), three were of ‘High’ quality (Gensby et al., 2014; NICE, 2019; Schaafsma et al., 2013), one of ‘Moderate’ quality, (Tingulstad et al., 2022) and three were of ‘Critically Low’ quality (Ishimaru et al., 2021; Oakman et al., 2016; Palmer et al., 2012).

The quantity of systematic review evidence across the other 13 conditions were as follows: Other (*n* = 16) (Axén et al., 2020; Gaillard et al., 2020; Gensby et al., 2014; Hoefsmit et al., 2012; Kojimahara et al., 2020; Lefever et al., 2018; Mikkelsen & Rosholm, 2018; NICE, 2019; Odeen et al., 2013; Schandelmaier et al., 2012; Tingulstad et al., 2022; Tompa et al., 2008; van Vilsteren et al., 2015; Venning et al., 2021; Verhoef et al., 2020; Vogel et al., 2017), Depression (*n* = 11) (Axén et al., 2020; Cullen et al., 2018; Gaillard et al., 2020; Gensby et al., 2014; Hoefsmit et al., 2012; Mikkelsen & Rosholm, 2018; NICE, 2019; Nieuwenhuijsen et al., 2020; Tingulstad et al., 2022; van Vilsteren et al., 2015; Venning et al., 2021), Anxiety (*n* = 7) (Axén et al., 2020; Gaillard et al., 2020; Gensby et al., 2014; Hoefsmit et al., 2012; Mikkelsen & Rosholm, 2018; NICE, 2019; van Vilsteren et al., 2015), Stress/burnout (*n* = 8) (Axén et al., 2020; Gensby et al., 2014; Hoefsmit et al., 2012; Mikkelsen & Rosholm, 2018; NICE, 2019; Tingulstad et al., 2022; Venning et al., 2021; Verhoef et al., 2020), Chronic pain (*n* = 4) (Cochrane et al., 2017; Franche et al., 2005; Venning et al., 2021; Verhoef et al., 2020), TBI (*n* = 3) (Gensby et al., 2014; Heathcote et al., 2019; Verhoef et al., 2020), Traumatic physical injury (*n* = 2) (Gensby et al., 2014; Heathcote et al., 2019), Stroke (*n* = 2) (Gensby et al., 2014; Verhoef et al., 2020), Arthritis (*n* = 2 (Gensby et al., 2014; Verhoef et al., 2020), Cancer (*n* = 1) (Gensby et al., 2014), Multiple sclerosis (*n* = 1) (Gensby et al., 2014) and Cardiac (*n* = 1) (Venning et al., 2021). No systematic review evidence met our inclusion criteria for people with dermatological conditions.

In general, systematic review evidence was predominantly split between those reporting a beneficial effect of the interventions being evaluated on RTW outcomes and those reporting inconclusive/weak evidence. Of the 15 reviews to report a positive effect of interventions on RTW outcomes or cost‐effectiveness (Brewer et al., 2007; Carroll et al., 2010; Cochrane et al., 2017; Franche et al., 2005; Gaillard et al., 2020; Heathcote et al., 2019; Kojimahara et al., 2020; Lefever et al., 2018; Mikkelsen & Rosholm, 2018; Neverdal, 2015; Nieuwenhuijsen et al., 2020; Schandelmaier et al., 2012; Tompa et al., 2008; van Geen et al., 2007; Verhoef et al., 2020), five were of ‘High’ quality (Gaillard et al., 2020; Heathcote et al., 2019; Nieuwenhuijsen et al., 2020; Schandelmaier et al., 2012; Verhoef et al., 2020), and two were of ‘Moderate’ quality (Cochrane et al., 2017; Mikkelsen & Rosholm, 2018).

Ten included cost‐effectiveness outcomes (see Tables 4 and 5) (Carroll et al., 2010; Cochrane et al., 2017; Franche et al., 2005; Gaillard et al., 2020; Lefever et al., 2018; NICE, 2019; Oakman et al., 2016; Palmer et al., 2012; Tingulstad et al., 2022; Tompa et al., 2008). Four of these reviews indicated that the interventions provided value for money (Carroll et al., 2010; Franche et al., 2005; Gaillard et al., 2020; Tompa et al., 2008), although the comparison of interest within one review was workplace‐based interventions versus non‐workplace based, so the findings are not relevant to our research question (Carroll et al., 2010). With the exception of one, (NICE, 2019) synthesis methods were usually descriptive or narrative in nature as the heterogeneity of the included reviews precluded statistical methods of analysis.

**Table 4:** Characteristics of reviews evaluating cost‐effectiveness.

Study	Interventions evaluated [Condition]	Synthesis methods^a^	Summary statement on cost‐effectiveness
Carroll 2010 (Carroll et al., 2010)	Interventions involving workplace [BP]	Narrative	***Evidence of positive effect***: Economic evaluations indicated that interventions with a workplace component are likely to be more cost effective than those without
Cochrane 2017 (Cochrane et al., 2017)	Interventions containing two or more elements of biopsychosocial model delivered as co‐ordinated programme [MSK]	Descriptive	***Mixed evidence***: Methodological differences in terms of the interventions, health systems and the types of economic analyses make it difficult to make direct comparisons across the trials. Three trials reported cost savings in health service costs and limiting productivity losses and also by reducing the number of patients transitioning to long‐term disability…Five trials reported no overall benefits in terms of cost savings
Franche 2005 (Franche et al., 2005)	Workplace based return‐to‐work interventions [MSK/Other pain]	Best‐evidence synthesis	***Evidence of positive effect***: strong evidence that work disability duration is significantly reduced by work accommodation offers and contact between healthcare provider and workplace; and moderate evidence that it is reduced by interventions which include early contact with worker by workplace, ergonomic work site visits, and presence of a RTW coordinator. For these five intervention components, there was moderate evidence that they reduce costs associated with work disability duration
Gaillard 2020 (Gaillard et al., 2020)	Mental health interventions with work‐focused components [MH]	Best‐evidence synthesis	***Evidence of positive effect***: Strong evidence of positive economic results for RTW interventions from employer and societal perspective. Interventions could take different forms: structured guidance with individualised support to implement problem‐solving treatment/elaborate an action plan, which could be accompanied by CBT; training for managers to enhance RTW communication with employees and internet‐based module with occupational physicians guidance. Not enough studies in the other categories combining the type of prevention (primary, secondary or tertiary) with the economic perspective (employers’, societal, employees’, healthcare system's) to produce evidence concerning the economic balance of interventions
Lefever 2018 (Lefever et al., 2018)	Disability Management [Disability]	Descriptive, Narrative	***No supporting evidence***: Not much evidence that Disability Management is cost‐effective
NICE, 2019 (NICE, 2019)	Interventions, programmes, policies or strategies that aim to increase RTW [MH, MSK, Other]	MA, narrative	***Evidence of mixed‐effect***: The committee noted the lack of health economic literature directly applicable to the UK. And even though it was mixed, they were mindful that overall it suggested interventions for people on sick leave due to musculoskeletal disorders including back pain or common mental health conditions to support them to return to work could be cost effective
Oakman 2016 (Oakman et al., 2016)	Workplace interventions (focused on individual or multi‐level) [MSK]	GRADE, narrative	***Evidence of mixed‐effect***: Individually focused interventions may make little or no difference to cost benefit. Multilevel focused interventions will probably increase cost benefit
Palmer 2012 (Palmer et al., 2012)	Interventions in community/workplace settings to reduce sickness absence/job loss [MSK]	Descriptive, narrative	***Inconclusive/weak evidence***: No study clearly proved or disproved a positive return on investment. No cost‐benefit analyses established statistically significant net economic benefits
Tingulstad (2022)(Tingulstad et al., 2022)	Work‐related interventions [Depression, Musculoskeletal, Stress/burnout, Other (CMD, adjustment and somatic disorders)]	MA, narrative	***No supporting evidence***: All secondary outcomes (including cost‐effectiveness) had very low certainty, mostly due to imprecision and risk of bias. Limited cost‐effectiveness data.
Tompa 2008 (Tompa et al., 2008)	Disability Management Interventions [Mixed]	Best‐evidence synthesis	***Evidence of positive effect***: Credible evidence supporting the financial benefits of disability management interventions for one industry cluster and several intervention components and features

Abbreviations: BP, back pain, CBT, cognitive‐behavioural therapy; MA, meta‐analysis; MSK, musculoskeletal difficulties; RTW, return to work.

a Pertaining to synthesis of cost‐outcomes.

**Table 5:** Cost‐effectiveness outcomes in prioritised systematic reviews.

Study	Interventions evaluated [Condition]	Synthesis methods^a^	Summary statement on cost‐effectiveness
Carroll 2010 (Carroll et al., 2010)	Interventions involving workplace [BP]	Narrative	***Evidence of positive effect***: Economic evaluations indicated that interventions with a workplace component are likely to be more cost effective than those without
Cochrane 2017 (Cochrane et al., 2017)	Interventions containing two or more elements of biopsychosocial model delivered as co‐ordinated programme [MSK]	Descriptive	***Mixed evidence***: Methodological differences in terms of the interventions, health systems and the types of economic analyses make it difficult to make direct comparisons across the trials. Three trials reported cost savings in health service costs and limiting productivity losses and also by reducing the number of patients transitioning to long‐term disability…Five trials reported no overall benefits in terms of cost savings
Franche 2005 (Franche et al., 2005)	Workplace‐based return‐to‐work interventions [MSK/Other pain]	Best‐evidence synthesis	***Evidence of positive effect***: strong evidence that work disability duration is significantly reduced by work accommodation offers and contact between healthcare provider and workplace; and moderate evidence that it is reduced by interventions which include early contact with worker by workplace, ergonomic work site visits, and presence of a RTW coordinator. For these five intervention components, there was moderate evidence that they reduce costs associated with work disability duration
Gaillard 2020 (Gaillard et al., 2020)	Mental health interventions with work‐focused components [MH]	Best‐evidence synthesis	***Evidence of positive effect***: Strong evidence of positive economic results for RTW interventions from employer and societal perspective. Interventions could take different forms: structured guidance with individualised support to implement problem‐solving treatment/elaborate an action plan, which could be accompanied by CBT; training for managers to enhance RTW communication with employees and internet‐based module with occupational physicians guidance. Not enough studies in the other categories combining the type of prevention (primary, secondary or tertiary) with the economic perspective (employers’, societal, employees’, healthcare system's) to produce evidence concerning the economic balance of interventions
Lefever 2018 (Lefever et al., 2018)	Disability Management [Disability]	Descriptive/Narrative	***No supporting evidence***: Not much evidence that Disability Management is cost‐effective
NICE, 2019 (NICE, 2019)	Interventions, programmes, policies or strategies that aim to increase RTW [MH, MSK, Other]	MA/Narrative/	***Evidence of mixed‐effect***: The committee noted the lack of health economic literature directly applicable to the UK. And even though it was mixed, they were mindful that overall it suggested interventions for people on sick leave due to musculoskeletal disorders including back pain or common mental health conditions to support them to return to work could be cost effective
Oakman 2016 (Oakman et al., 2016)	Workplace interventions (focused on individual or multi‐level) [MSK]	GRADE, narrative	***Evidence of mixed‐effect***: Individually focused interventions may make little or no difference to cost benefit. Multilevel focused interventions will probably increase cost benefit
Palmer 2012 (Palmer et al., 2012)	Interventions in community/workplace settings to reduce sickness absence/job loss [MSK]	Descriptive, narrative	***Inconclusive/weak evidence***: No study clearly proved or disproved a positive return on investment. No cost‐benefit analyses established statistically significant net economic benefits
Tingulstad 2022 (Tingulstad et al., 2022)	Work‐related interventions for return to work in people on sick leave [MH, MSK, Other]	MA/Narrative	***Evidence of mixed‐effect***: Three RCT's (291 participants) compared multi‐disciplinary rehabilitation with usual care. Two studies reported a cost‐effective experimental intervention. One study had not a cost‐effective experimental intervention. GRADE quality of evidence: Very Low.
Tompa 2008 (Tompa et al., 2008)	Disability Management Interventions [Mixed]	Best‐evidence synthesis	***Evidence of positive effect***: Credible evidence supporting the financial benefits of disability management interventions for one industry cluster and several intervention components and features

Abbreviations: BP, back pain; CBT, cognitive‐behavioural therapy; MA, meta‐analysis; MH, mental health; MSK, musculoskeletal difficulties; RTW, return to work.

a Pertaining to synthesis of cost‐outcomes.

## 6 DISCUSSION

### 6.1 Summary of main results

(We aimed to establish the volume, quality and characteristics of evidence relating to the effectiveness and cost‐effectiveness of workplace based, multi‐disciplinary OH interventions aiming to improve work‐related outcomes for employed adults. We found a substantial body of systematic review evidence relating to the effectiveness of multi‐disciplinary OH interventions to promote RTW, with 30 (of 101) rated as relevant to our research question. We produced an EGM to graphically represent the quality, quantity and basic features of these 30 systematic reviews. The map also highlights the primary evidence within these systematic reviews which aligns with the inclusion criteria for the EGM, grouped according to the reported finding regarding RTW and cost outcomes. This allows the map user to ‘drill down’ from the systematic review level and access links to the primary studies particularly relevant to their requirements. As such, the map is intended as an interactive resource and we suggest that readers navigate the EGM, accessed here https://eppi.ioe.ac.uk/cms/Portals/35/Maps/MN_Exeter_Feb22.html), and browse publications of interest.

### 6.2 Overall completeness and applicability of evidence

A visual examination of this map reveals a cluster of evidence on the effectiveness of OH interventions to promote RTW for people with musculoskeletal issues but numerous health conditions for which there are no high‐quality systematic reviews. Nine of the systematic reviews evaluated cost‐effectiveness outcomes. Most reviews were driven by the aim of treating specific conditions, rather than evaluating specific interventions, which contributed to the heterogeneity of review findings.

Where details of interventions were sufficiently reported, the systematic reviews often included a range of interventions within one broad category and, as a result, the features of these interventions tended to differ greatly from one another. In addition, the aims of the systematic reviews which met our eligibility criteria did not always align directly with the aims of our EGM, reducing the quantity of available evidence that was relevant to our aims, although the prioritisation of systematic reviews for the EGM helped mitigate this.

### 6.3 Areas of major gaps in the evidence

The EGM identifies where systematic review evidence in this area is lacking, or where existing evidence is of poor quality. Little to no systematic review evidence which met our inclusion criteria was found for cancer, stroke and dermatological conditions. The small number of systematic reviews included in the map relating to cardiac conditions, chronic pain and stress/burn out generally of ‘Critically Low’ quality, indicating the potential benefit of conducting further systematic reviews utilising more robust methods. Finally, six of the nine reviews demonstrating a significant positive impact of OH interventions for people living with musculoskeletal conditions were of ‘Critically Low’ (*n* = 5) or ‘Low’ (*n* = 1) methodological quality, which limits the confidence which can be placed in these findings and may represent an area where it may be particularly useful to conduct further systematic reviews.

Table 3 highlights the variation in compliance with items in the AMSTAR checklist contributing to the categorisation across the included systematic reviews. The failure to use robust methods to synthesise evidence has been observed in many other fields (Abbott et al., 2022; Shaw et al., 2022). This EGM highlights some of the implications of methodological shortcuts on future decision making using systematic reviews that have been conducted using poor methods.

### 6.4 Limitations of the EGM

In summary of the limitations already acknowledged above, half of the reviews included in the EGM were of ‘Low’ or ‘Critically Low’ quality and encompassed a highly heterogeneous array of health conditions and interventions. To support users of the EGM to access the evidence suited to their needs, we added a link to the primary studies within the comment section of each record, alongside a summary of the main findings with respect to return‐to‐work outcomes for each study. Hence, map users can ‘drill down’ to find out more about the primary studies that are most relevant to their requirments and circumstances.

The heterogeneity of populations and interventions included in primary studies within individual reviews necessitated the use of broad categories to form the overall structure of the EGM. This resulted in some primary studies sitting in multiple segments across the map. This duplication means that the quantity of evidence appears larger in some areas of the map but may be negated by map‐users seeking evidence most applicable to them. Due to resource limitations, studies meeting our inclusion criteria but not published in English were not included in the EGM.

It was not feasible to map some of more granular factors that might impact on outcomes (such as sickness absence duration, timing of the intervention, and social security arrangements) at the level of each SR. Whilst such factors were beyond the scope of this review, they could make fruitful avenues for future research.

## 7 AUTHORS’ CONCLUSIONS

This EGM provides an overview of the systematic review evidence regarding the effectiveness and cost‐effectiveness of occupational health interventions to support employed adults to return to work. This evidence is presented in an interactive evidence‐and‐gap map to allow users to access and view the evidence most suited to their needs. The heterogeneity of the systematic review evidence, and primary studies contained within, prevented us from being able to create a taxonomy of effective intervention features or professional groups.

### 7.1 Implications for research

The evidence and gap map also identifies where systematic review evidence in this area is lacking, or where existing evidence is of poor quality. These represent areas where it may be particularly useful to search for primary studies to explore whether further systematic reviews in these areas could be usefully undertaken. For example, little to no systematic review evidence that met our inclusion criteria was found for cardiac conditions, cancer, stroke and dermatological conditions. This evidence and gap map also highlights the primary studies within these reviews which are specifically relevant to our research aims and objectives.

The commissioning of a systematic review to establish if there is any qualitative evidence which seeks to understand the experiences of employees and employers, regarding occupational health interventions provided within their workplace, may help identify intervention features of that are most valued and those which are perceived as unhelpful. This could potentially offer the opportunity to link data from reviews of quantitative and qualitative evidence, using a qualitative comparative analysis, to investigate if the intervention features, perceived as helpful by employees/employers in supporting return to work, are linked with the effectiveness of the intervention.

### 7.2 Implications for policy and practice

This evidence and gap map has highlighted the bodies of systematic review evidence which relate to the effectiveness and/or cost‐effectiveness of occupational health interventions in supporting return to work. This evidence may be useful for supporting policy makers and commissioners of services to determine which occupational health interventions are most useful for supporting different population groups in different contexts. Occupational health professionals may find the content of the evidence and gap map useful in identifying systematic review evidence to support their practice.

## CONTRIBUTIONS OF AUTHORS

LS led the development of the interactive EGM and strategic planning for drafting the report and refining map categories. MN led with formatting and generation of content for the EGM, with SB, GJM, RG and JTC contributing towards its development.

LS, MN, HL, JTC and SGS carried out screening, data extraction and quality appraisal. LS, MN and HL carried out citation chasing.

SB designed and ran the search strategies and managed the bibliographic libraries.

LS led on the drafting, assembly and formatting of the final report. MN, SB, SGS and HL drafted sections of the report and read, provided feedback on, edited and approved the final version of the report. JTC, GJMT and RG read, provided feedback on, edited and approved the final version of the report.

JTC provided overall project management. GJMT, RG, JTC, SB, MN and LS contributed to the scoping process, refining of research questions and development of the protocol in collaboration with the protocol authorship team. LS led on stakeholder engagement/PPI, with support from all authors.

- **Content**:
  All authors have experience of health service and social care research. Although no authors have direct experience in occupational health interventions, stakeholders with expertise were involved throughout the development of the EGM.
- **EGM methods**:
  JTC, LS, MN, SB and RG have previously worked on evidence gaps maps. All authors have prior experience in conducting all stages of a systematic review.
- **Information retrieval**:
  SB has training and extensive experience in designing and implementing search strategies.

## DECLARATIONS OF INTEREST

Jo Thompson Coon is a member of the NIHR Health Technology Assessment General Funding Committee. All other authors report no conflict of interests.

## PLANS FOR UPDATING THE EGM

There are no current plans to update this EGM. However, the authors will consider updating the EGM in the future if relevant funding is available.

## DIFFERENCES BETWEEN PROTOCOL AND REVIEW

### 1 Search strategy

Only the reference lists of systematic reviews that met our inclusion criteria and were judged by two independent reviewers to be highly relevant to the aims and objectives of our review were checked for additional systematic reviews. This was a pragmatic decision, informed by the high number of systematic reviews eligible for inclusion in this review. Whilst this means any relevant systematic reviews within the reference lists of studies rated as ‘Medium’ or ‘Low’ relevance will not have been identified, the impact of this will have been mitigated somewhat through our extensive search strategies, including grey literature sources. Two independent reviewers applied the criteria used to identify highly relevant reviews as described in the inclusion criteria section (LS, MN, HL, SGS).

### 2 Application of inclusion criteria

Determining whether a systematic review met our inclusion criteria was often not straightforward. The inclusion criteria for the reviews included in the EGM were often broader than the aims of EGM, which meant that some of the primary studies included within a single review could be relevant to the aims of our research, whilst others could not. In addition, the information required to determine if the review, and/or the primary studies it included, met the inclusion for our EGM was often not fully reported at the level of the review. Examples of the uncertainties we had regarding whether the review met our inclusion criteria are provided in Table 6 below.

**Table 6:** Queries regarding inclusion criteria of included reviews.

PICO criteria	Potential uncertainties
Population	Was the population employed before receiving occupational health support?
	Was the population aged 16 or above?
Intervention	Was the intervention delivered in conjunction with workplace?
	Was the intervention delivered by an MDT?
Comparator	N/A
Outcome	Was a RTW outcome measured
Other	Did the review conduct an adequate synthesis of primary studies?

Abbreviations: MDT, multi‐disciplinary team; N/A, not applicable; RTW, return to work.

During the study selection process, we were over‐inclusive, including all systematic reviews that appeared to meet the eligibility criteria but tagged each review with the uncertainties encountered in applying the criteria.

### 3 Data extraction

We conducted data extraction in three stages.

In the first stage, summary data for each eligible review was extracted by one reviewer and checked by a second using Microsoft Excel (LS, SGS, HL, MN).

In a deviation from our protocol, due to the diversity of the systematic reviews which met our inclusion criteria, some of which were not closely aligned with our aims and research questions, we then categorised reviews as being of ‘High’, ‘Medium’, or ‘Low’ relevance to the research questions using the following information:

– Aim of systematic review;
– Number of uncertainties tagged against the review;
– Proportion of primary studies within each review that met the inclusion criteria for our review.

And awarded a relevance rating to each systematic review, as outlined below:

- High: Aim of systematic review directly relevant to our research question, with up to one uncertainty against the inclusion criteria;
- Medium: Aim of systematic review not completely compatible with theour researc question, with two uncertainties against the inclusion criteria;
- Low: Aim of systematic review not completely compatible with our research question with two‐three uncertainties against the inclusion criteria and/or limited number of relevant included primary studies.

Further detail of this process is provided in Supporting Information 1.

In the second stage of data extraction, we focused on reviews with ‘High’ and ‘Medium’ relevance to populate the evidence and gap map. No further data was extracted from reviews judged to be of ‘Low’ relevance to our research questions and these reviews were excluded from the EGM.

We developed a standardised data extraction form which was piloted by two reviewers (LS, MN) on a selection (*n* = 5) of included reviews. The data extraction form was amended following this, to account for revised Quality Appraisal criteria (as described below) and to add further detail regarding the country the review was conducted in addition to the countries eligible studies were conducted in as specified by the review inclusion criteria. The following information was extracted from each systematic review:

– Age of sample as cited in inclusion criteria;
– Country review conducted in;
– Country included primary studies conducted in (as reported in inclusion criteria);
– Health conditions of sample as cited in inclusion criteria;
– Intervention of interest;
– Area of work/sector/employer;
– Whether review inclusion criteria and/or synthesis strategy considered any of the PROGRESS criteria (place of residence, race/ethnicity/culture/language, gender/sex, religion, education, socio‐economic status, social capital) (Welch et al., 2019).
– RTW outcome main findings.

Data extraction was performed by one reviewer (MN, JTC) and checked by a second (LS), with disagreements being settled through discussion. EPPI‐Reviewer software was used to support data extraction (Thomas et al., 2010). In the third and final stage of data extraction, due to the often poor reporting of the characteristics of the included studies within the systematic reviews, where necessary we sought additional methodological detail from the primary studies.

### 4 Quality appraisal

Our protocol states our intention to quality appraise all the systematic reviews eligible for inclusion. However, due to the high number of systematic reviews eligible for inclusion, we proceeded with full data extraction for only those reviews rated as ‘High’ or ‘Medium’ relevance (defined above). This only excluded low relevance reviews and is unlikely to have impacted on the findings.

To provide an indicator of the quality of low‐relevance reviews we selected four items from the Collaboration for Environmental Evidence Synthesis Appraisal Tool (CEESAT) (Evidence, 2018, 2020).

1. Is approach to searching clearly defined, systematic and transparent?
2. Is search comprehensive?
3. Does the review critically appraise each study?
4. During appraisal is an effort made to minimise subjectivity

The items selected represent key characteristics/critical domains of robust methods as identified by a range of quality appraisal tools, for example, the DARE criteria (Petticrew et al., 1999). We selected corresponding items from CEESAT as we were in search of tool to explore quality in review level that would let us explore the quality of included reviews, and thus prioritise for full quality appraisal, at greater depth without needing to conduct full‐quality appraisal on each included systematic review. The CEESAT is an eight‐item checklist which supports an appraisal of methods used within systematic reviews, how transparently these methods are reported and how any limitations in quantity and quality of primary data may influence the synthesis. Administering the whole checklist to each of our included studies was infeasible. Instead, we used the four items above to develop to generate an overall quality rating for each included systematic review (see Supporting Information 1 for proxy quality ratings). Full quality appraisal was undertaken for systematic reviews which were of ‘High’ or ‘Medium’ relevance to our research questions, the process of which is described within the methods section of the main report.

## SOURCES OF SUPPORT

**Internal sources**

- No sources of support provided

**External sources**

- NIHR Exeter PRP Evidence Review Facility (Award ID NIHR200695)

## Supporting information

Supporting information.

Supporting information.
