# Supplementary material for: What is the volume, quality and characteristics of evidence relating to the effectiveness and cost‐effectiveness of multi‐disciplinary occupational health interventions aiming to improve work‐related outcomes for employed adults? An evidence and gap map of systematic reviews
Source: Campbell Syst Rev. 2024 May 14;20(2):e1412. doi: 10.1002/cl2.1412 (PMC11094349; doi:10.1002/cl2.1412)
Supplement: Supplementary file 1 — Supporting information. [file CL2-20-e1412-s001.docx]

## Excluded studies

Excluded at full text: original searches

| **Paper** | **Reason** |
| --- | --- |
| Aanesen, F., Berg, R., Lochting, I., Tingulstad, A., Eik, H., Storheim, K., . . . Oiestad, B. E. (2021). Motivational Interviewing and Return to Work for People with Musculoskeletal Disorders: A Systematic Mapping Review. Journal of occupational rehabilitation, 31(1), 63-71. doi:https://dx.doi.org/10.1007/s10926-020-09892-0 | Int (WP) |
| Aas, R. W., Tuntland, H., Holte, K. A., Røe, C., Lund, T., Marklund, S., & Moller, A. (2011). Workplace interventions for neck pain in workers. Cochrane database of systematic reviews, (4) | Int (MD) |
| Abidin, M., Yunus, F. W., Rasdi, H. F. M., & Kadar, M. Employment programmes for schizophrenia and other severe mental illness in psychosocial rehabilitation: a systematic review. British Journal of Occupational Therapy. doi:10.1177/0308022620980683 | Pop |
| Ahola, K., Toppinen-Tanner, S., & Seppanen, J. (2017). Interventions to alleviate burnout symptoms and to support return to work among employees with burnout: Systematic review and meta-analysis. Burnout Research, 4, 1-11. doi:10.1016/j.burn.2017.02.001 | Study |
| Alexander, L., & Cooper, K. (2019). Vocational rehabilitation for emergency services personnel: a scoping review. JBI database of systematic reviews and implementation reports, 17(10), 1999-2019. doi:https://dx.doi.org/10.11124/JBISRIR-2017-003747 | Study |
| Alexander, L., Cooper, K., Mitchell, D., & MacLean, C. (2017). Effectiveness of vocational rehabilitation on work participation in adults with musculoskeletal disorders: an umbrella review protocol. JBI database of systematic reviews and implementation reports, 15(6), 1518-1521. doi:https://dx.doi.org/10.11124/JBISRIR-2016-003133 | Protocol |
| Algeo, N., Bennett, K., & Connolly, D. (2021). Rehabilitation interventions to support return to work for women with breast cancer: a systematic review and meta-analysis: researchsquare.com. | Int (WP) |
| Amatya, B., Khan, F., & Galea, M. (2019). Rehabilitation for people with multiple sclerosis: an overview of Cochrane Reviews. The Cochrane database of systematic reviews, 1, CD012732. doi:https://dx.doi.org/10.1002/14651858.CD012732.pub2 | Int (WP) |
| Ansoleaga, E., Garrido, P., Dominguez, C., Castillo, S., Lucero, C., Tomicic, A., & Martinez, C. (2015). [Return to work enablers for workers with work-related mental illness]. Facilitadores del reintegro laboral en trabajadores con patologia mental de origen laboral: una revision sistematica., 143(1), 85-95. doi:https://dx.doi.org/10.4067/S0034-98872015000100011 | Lang |
| Austvoll-Dahlgren, A., Forsetlund, L., Munthe-Kaas, H. M., & Kirkehei, I. (2018). Effects of Support and Follow-Up Interventions for People with Severe Mental Illness. | Pop |
| Bethge, M. (2017). [Work-Related Medical Rehabilitation]. Medizinisch-beruflich orientierte Rehabilitation., 56(1), 14-21. doi:https://dx.doi.org/10.1055/s-0042-118579 | Lang |
| Bethge, M. (2017). "Medizinisch-beruflich orientierte Rehabilitation." Rehabilitation 56(1): 14-21. | Lang |
| Bisung, E., Elliott, S. J., & Clarke, A. E. (2018). Non-pharmacological interventions for enhancing the working life of patients with lupus: a systematic review. Lupus, 27(10), 1755-1756. doi:https://dx.doi.org/10.1177/0961203318777119 | Not SR |
| Bjork, M., Gerdle, B., Liedberg, G., Svanholm, F., Solmi, M., Thompson, T., . . . Dragioti, E. (2020). Interventions to facilitate return to work in adults with chronic non-malignant pain: a protocol for a systematic review and network meta-analysis. BMJ open, 10(11), e040962. doi:https://dx.doi.org/10.1136/bmjopen-2020-040962 | Protocol |
| Bloom, J., Dorsett, P., & McLennan, V. (2019). Occupational bonding after spinal cord injury: A review and narrative synthesis. Journal of Vocational Rehabilitation, 50(1), 109-120. doi:10.3233/JVR-180992 | Study |
| Bloom, J., Dorsett, P., & McLennan, V. (2020). Vocational rehabilitation to empower consumers following newly acquired spinal cord injury. Journal of Vocational Rehabilitation, 53(1), 131-144. doi:10.3233/JVR-201091 | Study |
| Boeltzig-Brown, Heike; Fleming, Allison R.; Heyman, Miriam; Gauthier, Martha; Cully, Julisa; Foley, Susan M. (2017) A Systematic Review of State Vocational Rehabilitation Agency-Based Literature *Rehabilitation Research, Policy, and Education*, v31 n4 p352-371 | Retrieval |
| BOMEL. (2005). Occupational health and safety support systems for small and medium sized enterprises. | Study |
| Brakenridge, C. L., Gane, E. M., Smits, E. J., Andrews, N. E., & Johnston, V. (2019). Impact of interventions on work-related outcomes for individuals with musculoskeletal injuries after road traffic crash: a systematic review protocol. Systematic reviews, 8(1), 247. doi:https://dx.doi.org/10.1186/s13643-019-1178-2 | Protocol |
| Brasure, M., Lamberty, G. J., Sayer, N. A., Nelson, N. W., Macdonald, R., Ouellette, J., & Wilt, T. J. (2013). Participation after multidisciplinary rehabilitation for moderate to severe traumatic brain injury in adults: a systematic review. Archives of physical medicine and rehabilitation, 94(7), 1398-1420. doi:https://dx.doi.org/10.1016/j.apmr.2012.12.019 | Int (WP) |
| Brouns, R., Espinoza, A. V., Goudman, L., Moens, M., & Verlooy, J. (2019). Interventions to promote work participation after ischaemic stroke: A systematic review. Clinical Neurology and Neurosurgery, 185. doi:10.1016/j.clineuro.2019.105458 | Int (WP) |
| Bumble, J. L., & Carter, E. W. (2020). Application of the World Café to disability issues: A systematic review. Journal of Disability Policy Studies. Doi:10.1177/1044207320949962 | Int |
| Burton, A. K., Kendall, N. A., Pearce, B. G., Birrell, L. N., & Bainbridge, L. C. (2008). Management of upper limb disorders and the biopsychosocial model. | Study |
| Bιάρνπ, A., & Ρόθα, O. (2018). Αποηελεζμαηικέρ ζηπαηηγικέρ επαγγελμαηικήρ (επαν) ένηαξηρ ηυν αηόμυν με αναπηπία/σπόνιερ παθήζειρ και πποβλήμαηα τςσικήρ ςγείαρ ζηην Εςπώπη: μια ζςζηημαηική ζύνθεζη επεςνηηικών ποπιζμάηυν. Interscientific Health Care, 10(1). | Lang |
| Canhete Pereira, R. M., & Monteiro, I. (2019). Vocational rehabilitation and return to work: integrative review. Revista brasileira de medicina do trabalho : publicacao oficial da Associacao Nacional de Medicina do Trabalho-ANAMT, 17(3), 441-455. doi:10.5327/Z1679443520190350 | Not SR |
| Capozzoli, M. C. (2018). Predictors of Return to Work after Multidisciplinary Rehabilitation Evaluation for Prolonged Post-concussion Symptoms (Doctoral dissertation, The University of Nebraska-Lincoln). | Study |
| Carlson, P. M., Boudreau, M. L., Davis, J., Johnston, J., Lemsky, C., McColl, M. A., . . . Smith, C. (2006). 'Participate to learn': A promising practice for community ABI rehabilitation. Brain Injury, 20(11), 1111-1117. doi:10.1080/02699050600955337 | Int |
| Carolyn, G., Michael, W., Jessica, B., & Katherine, J. I. (2016). Employment interventions for return-to-work in working-age adults following traumatic brain injury. Campbell Collaboration, 12. | Pop |
| Chou R, Deyo R, Friedly J, Skelly A, Hashimoto R, Weimer M, Fu R, Dana T, Kraegel P, Griffin J, Grusing S, Brodt E. Noninvasive Treatments for Low Back Pain. Comparative Effectiveness Review No. 169. (Prepared by the Pacific Northwest Evidence-based Practice Center under Contract No. 290-2012-00014-I.) AHRQ Publication No. 16-EHC004-EF. Rockville, MD: Agency for Healthcare Research and Quality; February 2016. www.effectivehealthcare.ahrq.gov/reports/final.cfm. | PV |
| Christie, L., Inman, J., Davys, D., & Cook, P. A. (2021). A systematic review into the effectiveness of occupational therapy for improving function and participation in activities of everyday life in adults with a diagnosis of depression. Journal of Affective Disorders, 282, 962-973. | Int (MD) |
| Clayton, S. (2012). Effectiveness of return-to-work interventions for disabled people: a systematic review of government initiatives focused on changing the behaviour of employers. European Journal of Public Health, 22(3). doi:http://dx.doi.org/10.1093/eurpub/ckr101 | OC |
| Clayton, S., Bambra, C., & Gosling, R. (2011). Assembling the evidence jigsaw: insights from a systematic review of UK studies of individual-focused return to work initiatives for disabled and long-term ill people. BMC Public Health, 11(170). | Pop |
| Clayton, S., Gosling, R., Povall, S., Misso, K., Bambra, C., & Whitehead, M. (2010). PATHWAYS TO WORK? INSIGHTS FROM A SYSTEMATIC REVIEW OF THE UK'S RETURN TO WORK INITIATIVES FOR DISABLED AND CHRONICALLY ILL PEOPLE. Journal of Epidemiology and Community Health, 64, A6-A6. doi:10.1136/jech.2010.120956.15 | Study |
| Collie Workplace-Based Interventions for Improving Return to Work after Musculoskeletal and Pain Related Conditions: A Systematic Review (draft) 2014 Missingr | Retrieval |
| Costi, S. (2019). Return to work of cancer survivors in Europe: systematic review of the literature. Proceedings of the International Scientific Conference. | OC |
| Cox, A., O’Regan, S., Denvir, A., Broughton, A., Pearmain, D., Tyers, C., & Hillage, J. (2008). What works in delivering improved health and safety outcomes: A review of the existing evidence. | Study |
| Crawford, J. O., Graveling, R. A., Cowie, H. A., & Dixon, K. (2010). The health safety and health promotion needs of older workers. Occupational medicine (Oxford, England), 60(3), 184-192. doi:https://dx.doi.org/10.1093/occmed/kqq028 | Int |
| Crowther, R. E. (2003). Vocational rehabilitation for people with severe mental illness: A systematic review; a survey of practice and a naturalistic follow up study. Vocational Rehabilitation for People With Severe Mental Illness: A Systematic Review; a Survey of Practice & a Naturalistic Follow Up Study, 1-1. | Pop |
| Crowther, R., Marshall, M., Bond, G., & Huxley, P. (2001). Vocational rehabilitation for people with severe mental illness. The Cochrane database of systematic reviews(2), CD003080. | Pop |
| Cruz, É. J. E., Souza, N. V. D., & Mauricio, V. C. (2011). Return of the person with intestinal stoma to the work force: a review. Revista Estima, 9(2), 31-38. | Lang |
| Cullen K, Franche RL, Clarke J, Irvin E.Working Paper #296. The role of organizational factors in workplacebased return-to-work interventions: A systematic review. Toronto: Institute for Work & Health, 2005. | Retrieval |
| Cullen, K. L., Irvin, A., & Collie, F. (2018). Effectiveness of Workplace Interventions in Return-to-Work for Musculoskeletal, Pain-Related and Mental Health Conditions An Update of the Evidence and Messages for Practitioners. Orthopaedic Physical Therapy Practice, 30(3), 179-179. | Abs |
| de Boer, A. G., Taskila, T., Tamminga, S. J., Frings-Dresen, M. H., Feuerstein, M., & Verbeek, J. H. (2011). Interventions to enhance return-to-work for cancer patients. The Cochrane database of systematic reviews(2), CD007569. doi:https://dx.doi.org/10.1002/14651858.CD007569.pub2 | Prev |
| de Boer, A., Taskila, T. K., Tamminga, S. J., Feuerstein, M., Frings-Dresen, M. H. W., & Verbeek, J. H. (2015). Interventions to enhance return-to-work for cancer patients. Cochrane Database of Systematic Reviews(9). doi:10.1002/14651858.CD007569.pub3 | Int (WP) |
| de Buck, P. D., Schoones, J. W., Allaire, S. H., & Vliet Vlieland, T. P. (2002). Vocational rehabilitation in patients with chronic rheumatic diseases: a systematic literature review. Seminars in arthritis and rheumatism, 32(3), 196-203. doi:10.1053/sarh.2002.34609 | Study |
| Demou, E., Vargas-Prada, S., Lalloo, D., & Avila-Palencia, I. (2016). OP63 Very early workplace sickness absence interventions: A systematic review and meta-analysis of their effectiveness: jech.bmj.com. | Abs |
| Désiron, H. A., De Rijk, A., Van Hoof, E., & Donceel, P. (2011). Occupational therapy and return to work: a systematic literature review. BMC Public Health, 11(1), 1-14. | Study |
| Desmeules, F., Boudreault, J., Dionne, C. E., Fremont, P., Lowry, V., MacDermid, J. C., & Roy, J.-S. (2016). Efficacy of exercise therapy in workers with rotator cuff tendinopathy: a systematic review. Journal of occupational health, 58(5), 389-403. | Int (MD) |
| Dewa, C. S., Loong, D., Trojanowski, L., & Bonato, S. (2018). The effectiveness of augmented versus standard individual placement and support programs in terms of employment: a systematic literature review. Journal of mental health (Abingdon, England), 27(2), 174-183. doi:https://dx.doi.org/10.1080/09638237.2017.1322180 | Pop |
| Dibben, P., Wood, G., & O’Hara, R. (2018). Do return to work interventions for workers with disabilities and health conditions achieve employment outcomes and are they cost effective? A systematic narrative review. Employee Relations, 40(6), 999-1014. doi:10.1108/ER-01-2017-0023 | Study |
| Doki, S., Harano, S., Shinada, K., Ohyama, A., & Kojimahara, N. (2018). [Return-to-work support programs for workers on sick leave: a systematic review and meta-analysis]. Sangyo eiseigaku zasshi = Journal of occupational health, 60(6), 169-179. doi:https://dx.doi.org/10.1539/sangyoeisei.2018-008-A | Lang |
| Donker-Cools, B., Daams, J., Wind, H., Frings-Dresen, M. (2016). Effective return-to-work interventions after acquired brain injury: A systematic review. Brain injury, 30(2), 113-131. | OC |
| Driessen, M. T., Proper, K. I., van Tulder, M. W., Anema, J. R., Bongers, P. M., & van der Beek, A. J. (2010). The effectiveness of physical and organisational ergonomic interventions on low back pain and neck pain: a systematic review. Occupational and environmental medicine, 67(4), 277-285. | Pop |
| du Plessis, C., Whitaker, L., & Hurley, J. (2020). Peer support workers in substance abuse treatment services: A systematic review of the literature. Journal of Substance Use, 25(3), 225-230. doi:10.1080/14659891.2019.1677794 | Study |
| Durand, M. J., Corbiere, M., Coutu, M. F., Reinharz, D., & Albert, V. (2014). A review of best work-absence management and return-to-work practices for workers with musculoskeletal or common mental disorders. Work-a Journal of Prevention Assessment & Rehabilitation, 48(4), 579-589. doi:10.3233/wor-141914 | Study |
| Ebrahim, S. (2014). Psychotherapy for depression in claimants receiving wage replacement benefits: review of the evidence. Journal of insurance medicine (New York, N.Y.), 44(1), 53-57. | Study |
| Egan, M., Bambra, C., Petticrew, M., & Whitehead, M. (2009). Reviewing evidence on complex social interventions: appraising implementation in systematic reviews of the health effects of organisational-level workplace interventions. Journal of Epidemiology & Community Health, 63(1), 4-11. | Study |
| Elders, L. A. M., Van der Beek, A. J., & Burdorf, A. (2000). Return to work after sickness absence due to back disorders–a systematic review on intervention strategies. International archives of occupational and environmental health, 73(5), 339-348. | Date |
| Fadyl, J. K., & McPherson, K. M. (2009). Approaches to vocational rehabilitation after traumatic brain injury: a review of the evidence. The Journal of head trauma rehabilitation, 24(3), 195-212. doi:https://dx.doi.org/10.1097/HTR.0b013e3181a0d458 | Pop |
| Fassier, J. B., Sarnin, P., Rouat, S., Peron, J., Kok, G., Letrilliart, L., & Lamort-Bouche, M. (2019). Interventions Developed with the Intervention Mapping Protocol in Work Disability Prevention: A Systematic Review of the Literature. Journal of occupational rehabilitation, 29(1), 11-24. doi:https://dx.doi.org/10.1007/s10926-018-9776-8 | Study |
| Fong, C. J., Murphy, K. M., Westbrook, J. D., & Markle, M. M. (2018). Psychological Interventions to Facilitate Employment Outcomes for Cancer Survivors: A Systematic Review and Meta-Analysis. Research on Social Work Practice, 28(1), 84-98. doi:10.1177/1049731515604741 | Int (WP) |
| Fong CJ, Murphy KM, Westbrook JD, Markle MM. Behavioral, psychological, educational, and vocational interventions to facilitate employment outcomes for cancer survivors: a systematic review. *Campbell Systematic Reviews* 2015;**11**:1-81. | Duplicate |
| Garrido Larrea, P., Ansoleaga Moreno, E., Tomicic Suñer, A., Domínguez Valverde, C., Castillo Vergara, S., Lucero Chenevard, C., & Martínez Guzmán, C. (2013). Mental Health Illness and the Return to Work Process: A systematic review. Cienc. Trab, 15(48), 105-113. Retrieved from http://www.epistemonikos.org/documents/8d85850f117fb91a2dffa40bd337d12f101a677c | Retrieval |
| Geurtsen, G. J., & Heugten, C. M. v. (2010). Comprehensive rehabilitation programmes in the chronic phase after severe brain injury: a systematic review: ingentaconnect.com. | OC |
| Graham, C. W., West, M. D., Bourdon, J. L., Inge, K. J., & Seward, H. E. (2016). Employment interventions for return to work in working aged adults following traumatic brain injury (TBI): A systematic review. Campbell Systematic Reviews, 12(1), i-133. | OC |
| Grimani, A., Bergström, G., Casallas, M. I. R., Aboagye, E., Jensen, I., & Lohela-Karlsson, M. (2018). Economic evaluation of occupational safety and health interventions from the employer perspective: A systematic review. Journal of occupational and environmental medicine, 60(2), 147. | Outcome |
| Gussenhoven, A. H., Jansma, E. P., Goverts, S. T., Festen, J. M., Anema, J. R., & Kramer, S. E. (2013). Vocational rehabilitation services for people with hearing difficulties: A systematic review of the literature. Work, 46(2), 151-164. | Int (MD) |
| Guzmán J, Esmail R, Malmivaara A, Karjalainen K, Irvin E, Bombardier C. (2006). Multidisciplinary biopsychosocial rehabilitation for chronic low back pain. Cochrane Database of Systematic Reviews 2006, Issue 2. [DOI: 10.1002/14651858.CD000963.pub2 | Prev |
| Guzman, J., Esmail, R., Karjalainen, K. A., Malmivaara, A., Irvin, E., & Bombardier, C. (2002). Multidisciplinary bio‐psycho‐social rehabilitation for chronic low‐back pain. Cochrane database of systematic reviews, (1). | Prev |
| Guzmán, J., Esmail, R., Karjalainen, K., Malmivaara, A., Irvin, E., & Bombardier, C. (2001). Multidisciplinary rehabilitation for chronic low back pain: systematic review. Bmj, 322(7301), 1511-1516. | Prev |
| Halonen, J. I., Atkins, S., & Hakulinen, H. (2017). Collaboration between employers and occupational health service providers: a systematic review of key characteristics: bmcpublichealth.biomedcentral.com. | Study |
| Hanif, S., Peters, H., & McDougall, C. (2017). A systematic review of vocational interventions for youth with physical disabilities. Factors in Studying. Doi:10.1108/S1479-354720170000010008 | Pop |
| Hanson, M. A., Burton, A. K., Kendall, N. A., Lancaster, R. J., & Pilkington, A. (2006). The costs and benefits of active case management and rehabilitation for musculoskeletal disorders. | Study |
| Harrison, J., Krieger, M. J., & Johnson, H. A. (2020). Review of Individual Placement and Support Employment Intervention for Persons with Substance Use Disorder. Substance Use & Misuse, 55(4), 636-643. doi:10.1080/10826084.2019.1692035 | Pop |
| Hesselstrand, M., & Samuelsson, K. (2015). Occupational therapy interventions in chronic pain–a systematic review. Occupational therapy. doi/abs/10.1002/oti.1396 | Int |
| Heymans, M. W., van Tulder, M. W., Esmail, R., Bombardier, C., & Koes, B. W. (2004). Back schools for non-specific low-back pain. The Cochrane database of systematic reviews(4), CD000261. | Int (MD) |
| Heymans, M. W., van Tulder, M. W., Esmail, R., Bombardier, C., & Koes, B. W. (2005). Back schools for nonspecific low back pain: a systematic review within the framework of the Cochrane Collaboration Back Review Group. Spine, 30(19), 2153-2163. | Int (MD) |
| Higgins, A., O'Halloran, P., & Porter, S. (2012). Management of long term sickness absence: a systematic realist review. Journal of occupational rehabilitation, 22(3), 322-332. doi:https://dx.doi.org/10.1007/s10926-012-9362-4 | Study |
| Higgins. (2006). Medical Advice on Return to Work with regard to Musculoskeletal Disorders | Study |
| Hillage, J., Rick, J., Pilgrim, H., Jagger, N., Carroll, C., & Booth, A. (2012). Evidence review 1: review of the effectiveness and cost effectiveness of interventions, strategies, programmes and policies to reduce the number of employees who move from short-term to long-term sickness absence and to help employees on long-term sickness absence return to work (May 2008). | Retrieval |
| Hoosain, M., de Klerk, S., & Burger, M. (2019). Workplace-based rehabilitation of upper limb conditions: a systematic review. Journal of occupational rehabilitation, 29(1), 175-193. | Int (MD) |
| Hou, W., Chi, C., Lo, H. D., Kuo, K. N., Chuang, H. (2013). Vocational rehabilitation for enhancing return-to-work in workers with traumatic upper limb injuries. | Prev |
| Hoving, J. L., Broekhuizen, M. L., & Frings-Dresen, M. H. (2009). Return to work of breast cancer survivors: a systematic review of intervention studies. BMC Cancer, 9, 117-117. doi:10.1186/1471-2407-9-117 | Study |
| Jetha, A., Shaw, R., Sinden, A. R., Mahood, Q., Gignac, M. A., McColl, M. A., & Martin Ginis, K. A. (2019). Work-focused interventions that promote the labour market transition of young adults with chronic disabling health conditions: a systematic review. Occupational and environmental medicine, 76(3), 189-198. doi:https://dx.doi.org/10.1136/oemed-2018-105454 | Pop |
| Jodi, E., Arunima, K., Richard, H. S. (2017). Return to Work After Young Stroke: A Systematic Review. | Abs |
| Joyce, S., Modini, M., Christensen, H., Mykletun, A., Bryant, R., Mitchell, P. B., & Harvey, S. B. (2016). Workplace interventions for common mental disorders: a systematic meta-review. Psychological medicine, 46(4), 683-697. | Int (MD) |
| Juszczyk, D., Doki, S., & Grime, P. (2017). … -based interventions to increase chances of sooner and sustained return to work in workers with common mental health disorders: a systematic review of the literature. Occupational and…. | Abs |
| Kamper SJ, Apeldoorn AT, Chiarotto A, Smeets RJ, Ostelo RW, Guzman J, van Tulder MW. Multidisciplinary biopsychosocial rehabilitation for chronic low back pain. Cochrane Database Syst Rev. 2014 Sep 2;(9):CD000963. doi: 10.1002/14651858.CD000963.pub3 | Prev |
| Kamper, S. J., Apeldoorn, A. T., Chiarotto, A., Smeets, R., Ostelo, R., Guzman, J., & van Tulder, M. W. (2015). Multidisciplinary biopsychosocial rehabilitation for chronic low back pain: Cochrane systematic review and meta-analysis. Bmj-British Medical Journal, 350. doi:10.1136/bmj.h444 | Int (WP) |
| Karjalainen K, Malmivaara A, van Tulder M, Roine R, Jauhiainen M, Hurri H, Koes B. (2001) Multidisciplinary biopsychosocial rehabilitation for subacute low back pain in working-age adults: a systematic review within the framework of the Cochrane Collaboration Back Review Group. Spine (Phila Pa 1976) | Prev |
| Karjalainen, K. A., Malmivaara, A., van Tulder, M. W., Roine, R., Jauhiainen, M., Hurri, H., & Koes, B. W. (2003). Multidisciplinary biopsychosocial rehabilitation for subacute low‐back pain among working age adults. Cochrane Database of Systematic Reviews, (2). | Prev |
| Kaspin, L. C., Gorman, K. M., & Miller, R. M. (2013). Systematic Review of Employer-Sponsored Wellness Strategies and their Economic and Health-Related Outcomes. Population health management, 16(1), 14-21. doi:10.1089/pop.2012.0006 | Study |
| Kausto, J., Kaila-Kangas, L., Pensola, T., Virta, L. J., & Shiri, R. (2017). Length of sickness absence and sustained return to work: systematic review of the research literature. European Journal of Public Health, 27, 250-250. WOS:000414389802165 | Abs |
| Kendall, E., Muenchberger, H., & Gee, T. (2006). Vocational rehabilitation following traumatic brain injury: a quantitative synthesis of outcome studies. Journal of Vocational Rehabilitation, 25(3), 149-160. Retrieved from http://www.epistemonikos.org/documents/a0ad127b0feb46a3a92589814e3e60e7b380b432 | Study |
| Khan F, Amatya B. Rehabilitation in Multiple Sclerosis: A Systematic Review of Systematic Reviews. Arch Phys Med Rehabil. 2017 Feb;98(2):353-367. doi: 10.1016/j.apmr.2016.04.016. | Int |
| Kojimahara, N., Fukumoto, M., Yoshikawa, E., Shinada, K., & Tsuiki, H. (2018). [Development process of Evidence-based "Return-to-work Guidance in Occupational Health 2017"]. Sangyo eiseigaku zasshi = Journal of occupational health, 60(5), 103-111. doi:https://dx.doi.org/10.1539/sangyoeisei.2017-030-B | Lang |
| Kornhaber, R., Wiechula, R., & McLean, L. (2015). The effectiveness of collaborative models of care that facilitate rehabilitation from a traumatic injury: a systematic review. JBI Evidence Synthesis, 13(8), 190-210. | E |
| Kowlakowsky-Hayner, S. A., & Tyerman, A. (2012). Vocational rehabilitation after traumatic brain injury: Models and services. NeuroRehabilitation, 31(1), 51-62. Retrieved from http://search.ebscohost.com/login.aspx?direct=true&db=ccm&AN=104477258&site=ehost-live | Study |
| Kupper, A., Mackenzie, S., & Heasman, T. (2004). The Challenge of Managing Upper Limb Disorders: How Can Health Professionals Become More Effective. Health and Safety Executive (HSE). | Study |
| Kyrou, K., Bobos, P., & Sheeran, L. (2019). A protocol for Exercise-based interventions in multidisciplinary rehabilitation for reducing fear avoidance in non-specific chronic low back pain: a systematic review and meta-analysis. | Protocol |
| Laires, P. A., Gouveia, M., & Canhão, H. (2017). Interventions aiming to reduce early retirement due to rheumatic diseases. Acta reumatologica portuguesa, 42(3), 240-248. | Study |
| Lancman, S., & Barroso, B. I. d. L. (2021). Mental health: Professional rehabilitation and the return to work - A systematic review. Work (Reading, Mass.). doi:https://dx.doi.org/10.3233/WOR-213489 | Study |
| Lee, J., & Kielhofner, G. (2010). Vocational intervention based on the Model of Human Occupation: a review of evidence. Scandinavian Journal of Occupational Therapy, 17(3), 177-190. doi:10.3109/11038120903082260 | Study |
| Leppin, A. L., Bora, P. R., Tilburt, J. C., Gionfriddo, M. R., Zeballos-Palacios, C., Dulohery, M. M., ... & Montori, V. M. (2014). The efficacy of resiliency training programs: a systematic review and meta-analysis of randomized trials. PloS one, 9(10), e111420. | OC |
| Lockwood et al 2015. Back in the Saddle: A Systematic Review of Occupational Therapy Interventions that Facilitate Return-to-Work Was missing | Study |
| Lounds Taylor, J., McPheeters, M. K., Sathe, N. A., Dove, D., Veenstra-VanderWeele, J., & Warren, Z. (2012). A Systematic Review of Vocational Interventions for Young Adults With Autism Spectrum Disorders. Pediatrics, 130(3), 531-538. doi:10.1542/peds.2012-0682 | Pop |
| Lunt, J., Lee, R., & Carter, L. (2007). Systematic Review of Preventative Behavioural Interventions for Dermal and Respiratory Occupational Health Hazards HSL/2007/36. | Int |
| MacEachen, E., Breslin, C., Kyle, N., Irvin, E., Kosny, A., Bigelow, P., ... & Scott-Dixon, K. (2008). Effectiveness and implementation of health and safety programs in small enterprises: a systematic review of quantitative and qualitative literature. Institute for Work and Health. | OC |
| Macedo, L. G., Smeets, R. J., Maher, C. G., Latimer, J., & McAuley, J. H. (2010). Graded activity and graded exposure for persistent nonspecific low back pain: a systematic review. Physical therapy, 90(6), 860-879. | Int (MD) |
| Magura, S., & Marshall, T. (2020). The Effectiveness of Interventions Intended to Improve Employment Outcomes for Persons with Substance Use Disorder: An Updated Systematic Review. Substance use & misuse, 55(13), 2230-2236. doi:https://dx.doi.org/10.1080/10826084.2020.1797810 | Study |
| McArthur 2013 Occupational Therapy Interventions for Individuals with Posttraumatic Stress Disorder Returning to Work: A Systematic Review Missing | Retrieval |
| McCluskey, A., Lovarini, M., Bennett, S., McKenna, K., Tooth, L., & Hoffmann, T. (2005). What evidence exists for work-related injury prevention and management? Analysis of an occupational therapy evidence database (OTseeker). British Journal of Occupational Therapy, 68(10), 447-456. doi:10.1177/030802260506801003 | Study |
| McKeown, L. P., & Cheshire, D. (2012). Vocational rehabilitation to help adults return to work: a systematic literature review. British Journal of Occupational Therapy, 75, 22-22. WOS:000209478500050 | Abs |
| McLeod, J. (2010). The effectiveness of workplace counselling: A systematic review. Counselling & Psychotherapy Research, 10(4), 238-248. doi:10.1080/14733145.2010.485688 | Int (MD) |
| Mewes, J. C., Steuten, L. M. G., & Ijzerman, M. J. (2012). Effectiveness of multidimensional cancer survivor rehabilitation and cost-effectiveness of cancer rehabilitation in general: a systematic review: ncbi.nlm.nih.gov. | OC |
| Michie, S., Williams, S., Michie, S., & Williams, S. (2003). Reducing work related psychological ill health and sickness absence: a systematic literature review. Occupational & Environmental Medicine, 60(1), 3-9. doi:10.1136/oem.60.1.3 | Study |
| Montano, D., Hoven, H., & Siegrist, J. (2014). Effects of organisational-level interventions at work on employees’ health: a systematic review. BMC public health, 14(1), 1-9. | OC |
| Myrhaug, H. T., Strom, V., Hafstad, E., Kirkehei, I., & Reinar, L. M. (2015). Retrieved from http://ovidsp.ovid.com/ovidweb.cgi?T=JS&PAGE=reference&D=medp&NEWS=N&AN=28510384 | Int (MD) |
| Nastasia, I., Coutu, M.-F., & Tcaciuc, R. (2014). Topics and trends in research on non-clinical interventions aimed at preventing prolonged work disability in workers compensated for work-related musculoskeletal disorders (WRMSDs): a systematic, comprehensive literature review. Disability and rehabilitation, 36(22), 1841-1856. doi:https://dx.doi.org/10.3109/09638288.2014.882418 | Study |
| Nevala, N., Pehkonen, I., Koskela, I., Ruusuvuori, J., & Anttila, H. (2015). Workplace Accommodation Among Persons with Disabilities: A Systematic Review of Its Effectiveness and Barriers or Facilitators. Journal of occupational rehabilitation, 25(2), 432-448. doi:10.1007/s10926-014-9548-z | Int |
| Nieuwenhuijsen, K., Bültmann, U., Neumeyer‐Gromen, A., Verhoeven, A. C., Verbeek, J. H., & Feltz‐Cornelis, C. M. (2008). Interventions to improve occupational health in depressed people. Cochrane Database of Systematic Reviews, (2). | Prev |
| Nieuwenhuijsen, K., Bültmann, U., Neumeyer-Gromen, A., Verhoeven, A. C., Verbeek, J. H., Feltz-Cornelis, C. M. (2014). Interventions to improve return to work in depressed people. | Prev |
| Noordik, E., van der Klink, J. J., Klingen, E. F., Nieuwenhuijsen, K., & van Dijk, F. J. (2010). Exposure-in-vivo containing interventions to improve work functioning of workers with anxiety disorder: a systematic review. BMC Public Health, 10(1), 598-598. doi:10.1186/1471-2458-10-598 | MD |
| Noyes, S., Sokolow, H., & Arbesman, M. (2018). Evidence for Occupational Therapy Intervention With Employment and Education for Adults With Serious Mental Illness: A Systematic Review. The American journal of occupational therapy : official publication of the American Occupational Therapy Association, 72(5), 7205190010p7205190011-7205190010p7205190010. doi:https://dx.doi.org/10.5014/ajot.2018.033068 | Pop |
| Oral, A., & Sindel, D. (2019). Are person- and work-directed interventions effective for enhancing return-to-work in patients with coronary heart disease? A Cochrane Review summary with commentary. Turkish journal of physical medicine and rehabilitation, 65(4), 402-405. doi:https://dx.doi.org/10.5606/tftrd.2019.00965 | Study |
| Pérez, B. D. D., Radford, K., Evangelou, N., & Nair, R. d. A Systematic Review of Vocational Rehabilitation for People with Multiple Sclerosis. | Study |
| Phillips 2021 Systematic Review of Intervention Research in Rehabilitation Counseling and Related SettingsFrom 2007 to 2018 Missing | Retrieval |
| Pike, A., Hearn, L., & de C Williams, A. C. (2016). Effectiveness of psychological interventions for chronic pain on health care use and work absence: systematic review and meta-analysis. Pain, 157(4), 777-785. | Int (MD) |
| Poiraudeau, S., Rannou, F., & Revel, M. (2007). Functional restoration programs for low back pain: a systematic review. In Annales de réadaptation et de médecine physique (Vol. 50, No. 6, pp. 425-429). Elsevier Masson. | Study |
| Poquet, N., Lin, C.-W. C., Heymans, M. W., van Tulder, M. W., Esmail, R., Koes, B. W., & Maher, C. G. (2016). Back schools for acute and subacute non-specific low-back pain. The Cochrane database of systematic reviews, 4, CD008325. doi:https://dx.doi.org/10.1002/14651858.CD008325.pub2 | Int (MD) |
| Preston, A., & Prior, Y. (2013). A systematic review of work interventions for people with rheumatoid arthritis. British Journal of Occupational Therapy, 76, 125-126. Retrieved from <Go to ISI>://WOS:000328855900288 | Abs |
| Prior, Y., & Hammond, A. (2014). Work rehabilitation for those with rheumatoid arthritis in the UK: A systematic review: usir.salford.ac.uk. | Study |
| Richardson, K. M., & Rothstein, H. R. (2008). Effects of occupational stress management intervention programs: a meta-analysis. Journal of occupational health psychology, 13(1), 69. | OC |
| Robinson, R., Okpo, E., & Mngoma, N. (2015). Interventions for improving employment outcomes for workers with HIV. The Cochrane database of systematic reviews(5), CD010090. doi:https://dx.doi.org/10.1002/14651858.CD010090.pub2 | Protocol |
| Saltychev, M., Eskola, M., Tenovuo, O., & Laimi, K. (2013). Return to work after traumatic brain injury: systematic review. Brain injury, 27(13-14), 1516-1527. | Int (MD) |
| Schaafsma, F., Schonstein, E., Ojajärvi, A., & Verbeek, J. (2011). Physical conditioning programs for improving work outcomes among workers with back pain. Scandinavian journal of work, environment & health, 1-5. | Prev |
| Schaafsma, F., Schonstein, E., Whelan, K. M., Ulvestad, E., Kenny, D. T., & Verbeek, J. H. (2010). Physical conditioning programs for improving work outcomes in workers with back pain. Cochrane Database of Systematic Reviews, (1). | Prev |
| Schandelmaier, S., Burkhardt, A., & Ebrahim, S. (2011). Insurance-based case management to reintegrate patients on sick leave: systematic review and meta-analysis. Occupational and…. | Abs |
| Shafi, R., & Colantonio, A. (2021). Assessing the effectiveness of workplace accommodations in facilitating return to work after traumatic brain injury: a systematic review protocol. BMJ open, 11(5), e041581. | Protocol |
| Silge, J., & Konrad, M. (2015). Berufliche Rehabilitation von Menschen mit substanzbezogenen Abhängigkeitserkrankungen - ein systematischer Review. Ergoscience, 10(2), 55-67. doi:10.2443/skv-s-2015-54020150202 | Lang |
| Silver, J. K., Baima, J., Newman, R., Galantino, M. L., & Shockney, L. D. (2013). Cancer rehabilitation may improve function in survivors and decrease the economic burden of cancer to individuals and society. Work-a Journal of Prevention Assessment & Rehabilitation, 46(4), 455-472. doi:10.3233/wor-131755 | Study |
| Staal, J. B., Hlobil, H., van Tulder, M. W., Koke, A. J. A., Smid, T., & van Mechelen, W. (2002). Return-to-work interventions for low back pain: a descriptive review of contents and concepts of working mechanisms. Sports medicine (Auckland, N.Z.), 32(4), 251-267.40-00004.pdf | Study |
| Stergiopoulos, E., Cimo, A., Cheng, C., Bonato, S., & Dewa, C. S. (2011). Interventions to improve work outcomes in work-related PTSD: a systematic review. BMC public health, 11, 838. doi:https://dx.doi.org/10.1186/1471-2458-11-838 | Int (MD) |
| Stergiou-Kita, M., Dawson, D., & Rappolt, S. (2012). Inter-professional clinical practice guideline for vocational evaluation following traumatic brain injury: a systematic and evidence-based approach. Journal of occupational rehabilitation, 22(2), 166-181. doi:https://dx.doi.org/10.1007/s10926-011-9332-2 | Study |
| Stergiou-Kita, M., Grigorovich, A., & Gomez, M. (2014). Development of an inter-professional clinical practice guideline for vocational evaluation following severe burn. Burns : journal of the International Society for Burn Injuries, 40(6), 1149-1163. doi:https://dx.doi.org/10.1016/j.burns.2014.01.001 | Study |
| Sundstrup, E., Seeberg, K. G. V., Bengtsen, E., & Andersen, L. L. (2020). A systematic review of workplace interventions to rehabilitate musculoskeletal disorders among employees with physical demanding work. Journal of occupational rehabilitation, 30(4), 588-612. | OC |
| Sutton, D. A., Cote, P., Wong, J. J., Varatharajan, S., Randhawa, K. A., Yu, H., . . . Stupar, M. (2016). Is multimodal care effective for the management of patients with whiplash-associated disorders or neck pain and associated disorders? A systematic review by the Ontario Protocol for Traffic Injury Management (OPTIMa) Collaboration. The spine journal : official journal of the North American Spine Society, 16(12), 1541-1565. doi:https://dx.doi.org/10.1016/j.spinee.2014.06.019 | Int (WP) |
| Sweetland, J., Howse, E., & Playford, E. D. (2012). A systematic review of research undertaken in vocational rehabilitation for people with multiple sclerosis. Disability and rehabilitation, 34(24), 2031-2038. doi:https://dx.doi.org/10.3109/09638288.2012.669019 | Study |
| Thisted, C. N., Labriola, M., Nielsen, C. V., Kristiansen, S. T., Strom, J., & Bjerrum, M. B. (2020). Managing employees' depression from the employees', co-workers' and employers' perspectives. An integrative review. Disability and Rehabilitation, 42(4), 445-459. doi:10.1080/09638288.2018.1499823 | RQ/ OC |
| Thomson, L., Neathey, F., & Rick, J. (2003). Best practice in rehabilitating employees following absence due to work-related stress. HSE Books. | Study |
| Tilbury, C., Schaasberg, W., Plevier, J. W., Fiocco, M., Vliet-Vlieland, T. P., & Nelissen, R. G. (2013). RETURN TO WORK AFTER TOTAL KNEE AND HIP ARTHROPLASTY: A SYSTEMATIC REVIEW. Annals of the Rheumatic Diseases, 72, 692-692. Retrieved from <Go to ISI>://WOS:000331587904139 | Abs |
| Trenaman, L. M., Miller, W. C., & Escorpizo, R. (2014). Interventions for improving employment outcomes among individuals with spinal cord injury: A systematic review. Spinal Cord, 52(11), 788-794. doi:10.1038/sc.2014.149 | Study |
| Trivedi, D. (2018). Cochrane Review Summary: Interventions to improve return to work in depressed people. Primary health care research & development, 19(2), 107-109. doi:https://dx.doi.org/10.1017/S1463423617000482 | Study |
| Tune, K., & Butler, J. (2012). Effectiveness of vocational rehabilitation after acquired brain injury: a systematic review. British Journal of Occupational Therapy, 75, 116-117. Retrieved from <Go to ISI>://WOS:000209478500260 | Study |
| Van De Cauter, J., Verbrugghe, M., Van De Velde, D., & Braeckman, L. (2019, September). Return-to-Work of Transgender Patients: What do We Know so Far? A Systematic Review. International Journal of Sexual Health. (Vol. 31, pp. A381-A382). | Protocol |
| van der Giessen, R. N. (2012). The effectiveness of graded activity in patients with non-specific low-back pain : a systematic review. Disability and Rehabilitation, 34(13). doi:http://dx.doi.org/10.3109/09638288.2011.631682 | Int (MD) |
| van Egmond, M. P., Duijts, S. F. A., van Muijen, P., van der Beek, A. J., & Anema, J. R. (2017). Therapeutic Work as a Facilitator for Return to Paid Work in Cancer Survivors. Journal of Occupational Rehabilitation, 27(1), 148-155. doi:10.1007/s10926-016-9641-6 | Study |
| van Oostrom, S. H., Driessen, M. T., de Vet, H. C., Franche, R. L., Schonstein, E., Loisel, P., ... & Anema, J. R. (2009). Workplace interventions for preventing work disability. Cochrane database of systematic reviews, (2). | Prev |
| van Tulder MW, Ostelo R, Vlaeyen JWS, et al. Behavioral treatment for chronic low back pain: a systematic review within the framework of the Cochrane Back Review Group. Spine 2000;25:2688–99. | Date |
| Varekamp, I., Verbeek, J. H., & van Dijk, F. J. (2006). How can we help employees with chronic diseases to stay at work? A review of interventions aimed at job retention and based on an empowerment perspective. International archives of occupational and environmental health, 80(2), 87-97. doi:10.1007/s00420-006-0112-9 | Study |
| Vargas-Prada, S., Demou, E., Lalloo, D., Avila-Palencia, I., Sanati, K. A., Sampere, M., . . . Macdonald, E. B. (2016). Effectiveness of very early workplace interventions to reduce sickness absence: a systematic review of the literature and meta-analysis. Scandinavian journal of work, environment & health, 42(4), 261-272. doi:https://dx.doi.org/10.5271/sjweh.3576 | Int (MD) |
| Verbeek, J. H., Martimo, K., Karppinen, J., Kuijer, P. P. F., Viikari-Juntura, E., & Takala, E. (2011). Manual material handling advice and assistive devices for preventing and treating back pain in workers. Cochrane Database of Systematic Reviews, N.PAG-N.PAG. | Int |
| Verhagen 2007 Exercise proves effective in a systematic review of work-related complaints of the arm, neck, or shoulder Missing | OC |
| Volter-Mahlknecht, S., & Rieger, M. A. (2014). Patient care at the interface between rehabilitation and occupational health physicians - a systematic literature review focusing health care organization. Deutsche Medizinische Wochenschrift, 139(31-32), 1609-1614. doi:10.1055/s-0034-1370189 | Lang |
| Waddell, G., & Burton, A. K. (2001). Occupational health guidelines for the management of low back pain at work: evidence review. Occupational medicine (Oxford, England), 51(2), 124-135. | Study |
| Waddell, G., Burton, A. K., & Kendall, N. A. S. (2016). Vocational rehabilitation–what works, for whom, and when? Report for the Vocational Rehabilitation Task Group. 2008. | Study |
| Wagner, S. L., Koehn, C., White, M. I., & Harder, H. G. (2016). Mental health interventions in the workplace and work outcomes: a best-evidence synthesis of systematic reviews: ncbi.nlm.nih.gov. | Study |
| Wagner, S. L., White, M. I., Schultz, I. Z., Williams-Whitt, K., Koehn, C., Dionne, C. E., ... & Wright, M. D. (2015). Social support and supervisory quality interventions in the workplace: a stakeholder-centered best-evidence synthesis of systematic reviews on work outcomes. The international journal of occupational and environmental medicine, 6(4), 189. | Study |
| Wei, X. J., Liu, X. F., & Fong, K. N. K. (2016). Outcomes of return-to-work after stroke rehabilitation: A systematic review. British Journal of Occupational Therapy, 79(5), 299-308. doi:10.1177/0308022615624710 | Int |
| Weir, R., & Nielson, W. R. (2001). Interventions for disability management. The Clinical journal of pain, 17(4 Suppl), S128-132. | Study |
| Wennman-Larsen, A., Petersson, L. M., & Alexanderson, K. (2010). Return to work after breast cancer-an exploratory systematic literature review. European Journal of Public Health, 20, 59-60. Retrieved from <Go to ISI>://WOS:000283675900155 | Study |
| Wiese, M., Kramer, J., Becker, C., Nentwig, V., Theodoridis, T., & Teske, W. (2009). [Back school - an update]. Ruckenschule heute., 147(2), 194-198. doi:https://dx.doi.org/10.1055/s-2008-1039234 | Lang |
| Williams A.C., Eccleston C., Morley S. (2012). Psychological therapies for the management of chronic pain (excluding headache) in adults. Cochrane Database Syst Rev 11:CD007407. https ://doi.org/10.1002/14651858.CD007407.pub3 | OC |
| Williams-Whitt, K., White, M. I., & Wagner, S. L. (2015). Job demand and control interventions: a stakeholder-centered best-evidence synthesis of systematic reviews on workplace disability: ncbi.nlm.nih.gov. | Study |
| Wong, J., Kallish, N., Crown, D., Capraro, P., Trierweiler, R., Wafford, Q. E., . . . Heinemann, A. W. Job Accommodations, Return to Work and Job Retention of People with Physical Disabilities: A Systematic Review. Journal of Occupational Rehabilitation. doi:10.1007/s10926-020-09954-3 | Int (MD) |
| Wright, M., Marsden, S., & Antonelli, A. (2004). Building an evidence base for the Health and Safety Commission Strategy to 2010 and beyond: a literature review of interventions to improve health and safety compliance. HSE Books. | Study |
| Wynne-Jones, G., Cowen, J., Jordan, J. L., Uthman, O., Main, C. J., Glozier, N., & van der Windt, D. (2014). Absence from work and return to work in people with back pain: a systematic review and meta-analysis. Occupational and environmental medicine, 71(6), 448-456. | Int (MD) |
| Yu, C. H., & Mathiowetz, V. (2014). Systematic review of occupational therapy–related interventions for people with multiple sclerosis: Part 1. Activity and participation. American Journal of Occupational Therapy. Retrieved from https://ajot.aota.org/article.aspx?articleid=1863111 | Study |
| Yuen, A., Sugeng, Y., Weiland, T. J., & Jelinek, G. A. (2010). Lifestyle and medication interventions for the prevention or delay of type 2 diabetes mellitus in prediabetes: a systematic review of randomised controlled trials. Australian and New Zealand journal of public health, 34(2), 172-178. | Int (MD) |
| Zampolini, M., Bernardinello, M., & Tesio, L. (2007). RTW in back conditions. Disability and rehabilitation, 29(17), 1377-1385. | Study |
| Zhang, X., & Zhou, L. (2013). Cochrane review summary for cancer nursing: interventions to enhance return to work for cancer patients. Cancer nursing, 36(1), 4-5. doi:https://dx.doi.org/10.1097/NCC.0b013e318277b564 | Study |

Abs=Abstract, E=Empty review, IMD=Intervention not multidisciplinary, IWP=Intervention not workplace-based, Lang=Language, OC=Outcome, Pop=Population, Prot=Protocol, PV=Previous Version of Included Review

Excluded at full text: update searches

| **Paper** | **Reason** |
| --- | --- |
| Afzal, K., Khattak, H., Sajjad, A., Hussain, S., Sarfraz, Z., Sarfraz, A., & Cherrez-Ojeda, I. (2022). Impact of Active Physiotherapy Rehabilitation on Pain and Global and Functional Improvement 1-2 Months after Lumbar Disk Surgery: A Systematic Review and Meta-Analysis. *Healthcare*, *10*(10), Article 1-IWP. https://doi.org/10.3390/healthcare10101943 | Int (IWP) |
| Aguey-Zinsou, M., Scanlan, J. N., & Cusick, A. A Scoping and Systematic Review of Employment Processes and Outcomes for Young Adults Experiencing Psychosis. *Community Mental Health Journal*, Article 1 Outcome. https://doi.org/10.1007/s10597-022-01056-z | Outcome |
| Atsidakou, N., Matsi, A. E., & Christakou, A. (2021). The effectiveness of exercise program after lumbar discectomy surgery. *Journal of clinical orthopaedics and trauma*, *16*, 99-105, Article 1 IWP. https://doi.org/https://dx.doi.org/10.1016/j.jcot.2020.12.030 | Int (IWP) |
| Breidenbach, C., Ernstmann, N., Heidkamp, P., Hiltrop, K., & Kowalski, C. (2022). Return to work in cancer patients: A systematic literature review on predictors in Germany. *Gesundheitswesen*, *84*(08/09), 808-809, Article 1 Abs. https://doi.org/10.1055/s-0042-1753819 | Abstract |
| Butink, M. H. P., Webers, C., Verstappen, S. M. M., Falzon, L., Betteridge, N., Wiek, D., Woolf, A. D., Stamm, T. A., Burmester, G. R., Bijlsma, J. W. J., Christensen, R., & Boonen, A. (2023). Non-pharmacological interventions to promote work participation in people with rheumatic and musculoskeletal diseases: a systematic review and meta-analysis from the EULAR taskforce on healthy and sustainable work participation. *Rmd Open*, *9*(1), Article 1 STU (not all quality appraised). https://doi.org/10.1136/rmdopen-2022-002903 | Study |
| Cnockaert, E., Bernaers, L., Braeckman, L., Mairiaux, P., Poot, O., & Willems, T. (2022). Does multidisciplinary therapy improve pain, functionality and return to work in employed (sub)acute low back pain sufferers? A systematic review...Pain Science in Motion IV Congress 2022, May 19-20th, Maastricht, Netherlands. *Pain Practice*, *22*, 31-32, Article 1 abs. https://doi.org/10.1111/papr.13128 | Abstract |
| Coopmans, L., Aliaga, J. A., Metsemakers, W. J., Sermon, A., Misselyn, D., Nijs, S., & Hoekstra, H. (2022). Accelerated Rehabilitation in Non-operative Management of Acute Achilles Tendon Ruptures: A Systematic Review and Meta-analysis. *Journal of Foot & Ankle Surgery*, *61*(1), 157-162, Article 1 IMD. https://doi.org/10.1053/j.jfas.2021.07.007 | Int (IMD) |
| David, S., Aaroke, A., Solomon, H., Roy, N., Lundborg, C. S., & Wanberg, M. G. (2021). Return to work and quality of life after trauma: A systematic review. *European Journal of Public Health*, *31*, Article 1 Abs. <Go to ISI>://WOS:000713802501020 | Abstract |
| Dunn, J. A., Hackney, J. J., Martin, R. A., Tietjens, D., Young, T., Bourke, J. A., Snell, D. L., Nunnerley, J. L., Hall, A., & Derrett, S. (2021). Development of a Programme Theory for Early Intervention Vocational Rehabilitation: A Realist Literature Review. *Journal of occupational rehabilitation*, Article 1 Study (QA v.limited/non existent). https://doi.org/10.1007/s10926-021-10000-z | Study |
| Edger-Lacoursiere, Z., Deziel, E., & Nedelec, B. (2022). Rehabilitation interventions after hand burn injury in adults: A systematic review. *Burns : journal of the International Society for Burn Injuries*, Article 1 IMD. https://doi.org/https://dx.doi.org/10.1016/j.burns.2022.05.005 | Int (IMD) |
| Edney, L. C., Roseleur, J., Gray, J., Koczwara, B., & Karnon, J. (2022). Mapping a decade of interventions to address the supportive care needs of individuals living with or beyond cancer: a scoping review of reviews. *Supportive care in cancer : official journal of the Multinational Association of Supportive Care in Cancer*, *30*(5), 3793-3804, Article 1 study. https://doi.org/https://dx.doi.org/10.1007/s00520-021-06713-9 | Study |
| Fowler Davis, S., Farndon, L., Harrop, D., Nield, L., Manson, J., Lawrence, J., Tang, S., Pownall, S., Elliott, J., Charlesworth, L., & Hindle, L. (2021). A rapid review and expert identification of the Allied Health Professions' interventions as a contribution to public health outcomes. *Public health in practice (Oxford, England)*, *2*, 100067, Article 1 STU. https://doi.org/https://dx.doi.org/10.1016/j.puhip.2020.100067 | Study |
| Gadager, B. B., Tang, L. H., Ravn, M. B., Doherty, P., Harrison, A., Christensen, J., Taylor, R. S., Zwisler, A.-D., & Maribo, T. (2022). Benefits of cardiac rehabilitation following acute coronary syndrome for patients with and without diabetes: a systematic review and meta-analysis. *BMC cardiovascular disorders*, *22*(1), 295, Article 1 outcome. https://doi.org/https://dx.doi.org/10.1186/s12872-022-02723-5 | Outcome |
| Guo, Y.-J., Tang, J., Li, J.-M., Zhu, L.-L., & Xu, J.-S. (2021). Exploration of interventions to enhance return-to-work for cancer patients: A scoping review. *Clinical rehabilitation*, *35*(12), 1674-1693, Article 1 study. https://doi.org/https://dx.doi.org/10.1177/02692155211021706 | Study |
| Hellstrom, L., Pedersen, P., Christensen, T. N., Wallstroem, I. G., Bojesen, A. B., Stenager, E., Bejerholm, U., van Busschbach, J., Michon, H., Mueser, K. T., Reme, S. E., White, S., & Eplov, L. F. (2021). Vocational Outcomes of the Individual Placement and Support Model in Subgroups of Diagnoses, Substance Abuse, and Forensic Conditions: A Systematic Review and Analysis of Pooled Original Data. *Journal of occupational rehabilitation*, *31*(4), 699-710, Article 1 POP. https://doi.org/https://dx.doi.org/10.1007/s10926-021-09960-z | Population |
| Holmes, J., McQueen, J., Perez, B. D., Radford, K., Blake, H., & Smith, B. (2022). The effectiveness of occupational therapy supporting return to work for people who sustain serious injuries or develop long-term (physical or mental) health conditions: a systematic review. *British Journal of Occupational Therapy*, *85*(8_SUPPL), 12-12, Article 1 ABS. <Go to ISI>://WOS:000828709200019 | Abstract |
| Kobayashi, M., Sezai, I., Ishikawa, T., & Masujima, M. (2022). Psychological and educational support for cancer patients who return to work: A scoping review. *Work (Reading, Mass.)*, *73*(1), 291-300, Article 1 STU. https://doi.org/https://dx.doi.org/10.3233/WOR-205326 | Study |
| Kuhnow, A., Kuhnow, J., Ham, D., & Rosedale, R. (2021). The McKenzie Method and its association with psychosocial outcomes in low back pain: a systematic review. *Physiotherapy theory and practice*, *37*(12), 1283-1297, Article 1 IMD. https://doi.org/https://dx.doi.org/10.1080/09593985.2019.1710881 | Int (IMD) |
| Llinos Haf, S., Annie, H., Abraham, M., Bethany, F. A., Jacob, D., Kalpa, P., Dyfrig, H., Deb, F., Clare, W., Rhiannon Tudor, E., Ruth, L., Alison, C., & Adrian, G. E. (2023). What interventions or best practice are there to support people with Long COVID, or similar post-viral conditions or conditions characterised by fatigue, to return to normal activities: a rapid review. *medRxiv*, Article 1 IMD/IWP. https://doi.org/10.1101/2023.01.24.23284947 | Int (IMD) |
| Mallick, S., & Islam, M. S. (2022). The impact of co-location employment partnerships within the Australian mental health service and policy context: A systematic review. *International journal of mental health nursing*, *31*(5), 1125-1140, Article 1 Outcome. https://doi.org/https://dx.doi.org/10.1111/inm.13007 | Outcome |
| Mangone, M., Agostini, F., de Sire, A., Cacchio, A., Chiaramonte, A., Butterini, G., Martano, A., Paoloni, M., Bernetti, A., & Paolucci, T. (2022). Effect of virtual reality rehabilitation on functional outcomes for return-to-work patients with Parkinson's disease: An umbrella review of systematic reviews. *NeuroRehabilitation*, *51*(2), 201-211, Article 1 IMD. https://doi.org/https://dx.doi.org/10.3233/NRE-220029 | Int (IMD) |
| Massen, F. K., Shoap, S., Vosseller, J. T., Fan, W., Usseglio, J., Boecker, W., Baumbach, S. F., & Polzer, H. (2022). Rehabilitation following operative treatment of acute Achilles tendon ruptures: a systematic review and meta-analysis. *EFORT open reviews*, *7*(10), 680-691, Article 1 IWP. https://doi.org/https://dx.doi.org/10.1530/EOR-22-0072 | Int (IWP) |
| Momsen, A. H., Ørtenblad, L., & Maribo, T. (2021). Effective rehabilitation interventions and participation among people with multiple sclerosis: an overview of reviews. *Annals of physical and rehabilitation medicine*, 101529, Article 1 duplicate. https://doi.org/10.1016/j.rehab.2021.101529 | Outcome |
| Momsen, A.-M. H., Ortenblad, L., & Maribo, T. (2022). Effective rehabilitation interventions and participation among people with multiple sclerosis: An overview of reviews. *Annals of physical and rehabilitation medicine*, *65*(1), 101529, Article 1 Outcome (only Sweetland potentially relevant = already scr). https://doi.org/https://dx.doi.org/10.1016/j.rehab.2021.101529 | Duplicate |
| Norlund, A., Ropponen, A., & Alexanderson, K. (2009). Multidisciplinary interventions: review of studies of return to work after rehabilitation for low back pain. Journal of rehabilitation medicine, 41(3), 115-121.W | Duplicate |
| Nowrouzi-Kia, B., Nadesar, N., Sun, Y., Ott, M., Sithamparanathan, G., & Thakkar, P. (2022). Prevalence and predictors of return to work following a spinal cord injury using a work disability prevention approach: A systematic review and meta-analysis. *Trauma*, *24*(1), 14-23, Article 1 Outcome. https://doi.org/10.1177/14604086211033083 | Outcome |
| Nutarelli, S., Delahunt, E., Cuzzolin, M., Delcogliano, M., Candrian, C., & Filardo, G. (2021). Home-Based vs Supervised Inpatient and/or Outpatient Rehabilitation Following Knee Meniscectomy: A Systematic Review and Meta-analysis. *JAMA network open*, *4*(5), e2111582, Article 1 IWP. https://doi.org/https://dx.doi.org/10.1001/jamanetworkopen.2021.11582 | Int (IWP) |
| Paz, L. E. S., Bezerra, B., Pereira, T. M. M., & da Silva, W. E. (2021). COVID-19: the importance of physical therapy in the recovery of workers' health. *Revista brasileira de medicina do trabalho : publicacao oficial da Associacao Nacional de Medicina do Trabalho-ANAMT*, *19*(1), 94-106, Article 1 study (no QA). https://doi.org/10.47626/1679-4435-2021-709 | Study |
| Proffitt, R., Boone, A., Hunter, E. G., Schaffer, O., Strickland, M., Wood, L., & Wolf, T. J. (2022). Interventions to Improve Social Participation, Work, and Leisure Among Adults Poststroke: A Systematic Review. *The American journal of occupational therapy : official publication of the American Occupational Therapy Association*, *76*(5), Article 1 STUDY. https://doi.org/https://dx.doi.org/10.5014/ajot.2022.049305 | Study |
| Sadeghi, M., Rahiminam, H., Amerizadeh, A., Masoumi, G., Heidari, R., Shahabi, J., Mansouri, M., & Roohafza, H. (2021). Prevalence of Return to Work in Cardiovascular Patients After Cardiac Rehabilitation: A Systematic Review and Meta-analysis. *Current problems in cardiology*, 100876, Article 1 duplicate. https://doi.org/10.1016/j.cpcardiol.2021.100876 | Study |
| Sadeghi, M., Rahiminam, H., Amerizadeh, A., Masoumi, G., Heidari, R., Shahabi, J., Mansouri, M., & Roohafza, H. (2022). Prevalence of Return to Work in Cardiovascular Patients After Cardiac Rehabilitation: A Systematic Review and Meta-analysis. *Current problems in cardiology*, *47*(7), 100876, Article 1 Study (SS). https://doi.org/https://dx.doi.org/10.1016/j.cpcardiol.2021.100876 | Duplicate |
| Sleight, A. G., Gerber, L. H., Marshall, T. F., Livinski, A., Alfano, C. M., Harrington, S., Flores, A. M., Virani, A., Hu, X., Mitchell, S. A., Varedi, M., Eden, M., Hayek, S., Reigle, B., Kerkman, A., Neves, R., Jablonoski, K., Hacker, E., Sun, V., . . . Stout, N. L. (2022). A Systematic Review of Functional Outcomes in Cancer Rehabilitation Research. *Archives of physical medicine and rehabilitation*, Article 1 duplicate. https://doi.org/10.1016/j.apmr.2022.01.142 | Duplicate |
| Speyer, R., Chen, Y. W., Kim, J. H., Wilkes-Gillan, S., Nordahl-Hansen, A. J., Wu, H. C., & Cordier, R. (2022). Non-pharmacological Interventions for Adults with Autism: a Systematic Review of Randomised Controlled Trials. *Review Journal of Autism and Developmental Disorders*, *9*(2), 249-279, Article 1 outcome. https://doi.org/10.1007/s40489-021-00250-1 | Outcome |
| Stehle L., Hoosain M., van Niekerk L. (2022) A systematic review of work-related interventions for breast cancer survivors: Potential contribution of occupational therapists. *Work*, 72(1):59-73. doi: 10.3233/WOR-210053. PMID: 35431214. | Study |
| Stratton, E., Lampit, A., Choi, I., Malmberg Gavelin, H., Aji, M., Taylor, J., Calvo, R. A., Harvey, S. B., & Glozier, N. (2022). Trends in Effectiveness of Organizational eHealth Interventions in Addressing Employee Mental Health: Systematic Review and Meta-analysis. *Journal of medical Internet research*, *24*(9), e37776, Article 1 IWP. https://doi.org/https://dx.doi.org/10.2196/37776 | Int (IWP) |
| Su, H., Dreesmann, N., Bridges, E., Hough, C. T., & Thompson, H. (2021). Return to work after critical illness: Systematic review, meta-analysis, and meta-regression. *Crit. Care Med.*, *49*(1 SUPPL 1), 220-None, Article 1 ABS. https://doi.org/10.1097/01.ccm.0000727724.94428.57 | Abstract |
| Thomas, F., & Morgan, R. L. (2021). Evidence-based job retention interventions for people with disabilities: A narrative literature review. *Journal of Vocational Rehabilitation*, *54*(2), 89-101, Article 1 STU. https://doi.org/10.3233/JVR-201122 | Study |
| Thompson, J. L., Holloway, K., Karyczak, S., Serody, M. R., Lane, I. A., Ellison, M. L., Gill, K. J., Davis, M., & Mullen, M. G. (2022). Evaluating Educational and Employment Services for Young People With Psychiatric Conditions: A Systematic Review. *Psychiatric services (Washington, D.C.)*, *73*(7), 787-800, Article 1 Outcome. https://doi.org/https://dx.doi.org/10.1176/appi.ps.202000033 | Outcome |
| Visser, E., de Klerk, S., Jacobs-Nzuzi Khuabi, L.-A., & Joubert, M. (2021). Occupation-based intervention in therapy for upper limb musculoskeletal conditions: A systematic review. *Hand Therapy*, *26*(4), 146-158, Article 1 outcome. https://doi.org/10.1177/17589983211054643 | Outcome |
| Vitturi, B. K., Rahmani, A., Dini, G., Montecucco, A., Debarbieri, N., Sbragia, E., Bandiera, P., Ponzio, M., Battaglia, M. A., Manacorda, T., Persechino, B., Buresti, G., Inglese, M., & Durando, P. (2022). Occupational outcomes of people with multiple sclerosis: a scoping review. *BMJ open*, *12*(7), e058948, Article 1 study. https://doi.org/https://dx.doi.org/10.1136/bmjopen-2021-058948 | Study |
| Young, V. M., Hill, J. R., Patrini, M., Negrini, S., & Arienti, C. (2022). Overview of Cochrane Systematic Reviews of Rehabilitation Interventions for Persons with Traumatic Brain Injury: A Mapping Synthesis. *Journal of clinical medicine*, *11*(10), Article 1 IMD. https://doi.org/https://dx.doi.org/10.3390/jcm11102691 | Int (IMD) |
| Zheng, K., Stern, B. Z., Wafford, Q. E., & Kohli-Lynch, C. N. (2022). Trial-Based Economic Evaluations of Supported Employment for Adults with Severe Mental Illness: A Systematic Review. *Administration and policy in mental health*, *49*(3), 440-452, Article 1 Pop. https://doi.org/https://dx.doi.org/10.1007/s10488-021-01174-y | Population |

# Appendices

## 1 Search strategies

### Bibliographic database searches

Database: Cochrane Database of Systematic Reviews
Host: Cochrane Library
Issue: Issue 6 of 12, June 2021
Date Searched: 28/6/2021
Searcher: SB
Hits: 112
Strategy:

#1 (return* near/3 work*):ti,ab,kw

#2 ("back to work"):ti,ab,kw

#3 ((return* near/3 (occupation* or employ*))):ti,ab,kw

#4 MeSH descriptor: [Return to Work] this term only

#5 ((reentry or re entry or reenter* or "re enter*") near/3 work*):ti,ab,kw

#6 ((reentry or re entry or reenter* or "re enter*") near/3 (occupation* or employ*)):ti,ab,kw

#7 ((barrier* or facilitator*) near/2 (employ* or occupation* or work*)):ti,ab,kw

#8 ("vocational rehabilitation"):ti,ab,kw

#9 ("work rehabilitation"):ti,ab,kw

#10 ("occupational rehabilitation"):ti,ab,kw (Word variations have been searched)

#11 MeSH descriptor: [Rehabilitation, Vocational] this term only

#12 “disability management”:ti,ab,kw

#13 (Office for National Statistics, -#12)

#14 (sick* near/2 (leave or absence)):ti,ab,kw

#15 MeSH descriptor: [Sick Leave] this term only

#16 “case management”:ti,ab,kw

#16 #14 or #15 or #16

#17 (occupational near/2 (health or medicine or therap*)):ti,ab,kw

#18 MeSH descriptor: [Occupational Health] this term only

#19 MeSH descriptor: [Occupational Therapy] this term only

#20 (Kniffin et al., -#19)

#21 #16 AND #20

#22 #13 OR #21

Notes: date limited 2001 to date of search

Database: Business Source Complete
Host: EBSCO
Issue: n/a
Date Searched: 28/6/2021
Searcher: SB
Hits: 37
Strategy:

1. TI ((return* OR back) N2 work*) OR AB ((return* OR back) N2 work*)
2. TI ( return* N2 (occupation* or employ*) ) OR AB ( return* N2 (occupation* or employ*) )
3. DE "RETURN to work programs"
4. TI ( (reentry or re entry or reenter* or "re enter*") N2 work* ) OR AB ( (reentry or re entry or reenter* or "re enter*") N2 work* )
5. TI ( (reentry or re entry or reenter* or "re enter*") N2 (occupation* or employ*) ) OR AB ( (reentry or re entry or reenter* or "re enter*") N2 (occupation* or employ*) )
6. TI ( (barrier* or facilitator*) N1 (employ* or occupation* or work*) ) OR AB ( (barrier* or facilitator*) N1 (employ* or occupation* or work*) )
7. TI "vocational rehabilitation" OR AB "vocational rehabilitation"
8. TI ((work OR occupational) N0 rehabilitation) OR AB ((work OR occupational) N0 rehabilitation)
9. TI "disability management" OR AB "disability management"
10. DE "VOCATIONAL rehabilitation" OR DE "EMPLOYMENT of blind people" OR DE "EMPLOYMENT of deaf people" OR DE "SHELTERED workshops" OR DE "SUPPORTED employment"
11. S1 OR S2 OR S3 OR S4 OR S5 OR S6 OR S7 OR S8 OR S9 OR S10
12. TI (( sick* N1 (leave or absence) ) OR “case management”) OR AB (( sick* N1 (leave or absence) ) OR “case management”)
13. DE "SICK leave"
14. S12 OR S13
15. TI ( occupational N1 (health or medicine or therap*) ) OR AB ( occupational N1 (health or medicine or therap*) )
16. DE "OCCUPATIONAL health services" OR DE "EMPLOYEE health promotion" OR DE "OCCUPATIONAL medicine"
17. S15 OR S16
18. S14 AND S17
19. S11 OR S18
20. TI ( (cochrane or cost or effectiveness or implementation or rapid or systematic or "state of the art" or umbrella) N1 (overview* or review* or synthes*) ) OR AB ( (cochrane or cost or effectiveness or implementation or rapid or systematic or "state of the art" or umbrella) N1 (overview* or review* or synthes*) )
21. TI ( "meta analy*" or metaanaly* or metasynthe* or "meta synthe*" ) OR AB ( "meta analy*" or metaanaly* or metasynthe* or "meta synthe*" )
22. TI "review* of reviews" OR AB "review* of reviews"
23. S20 OR S21 OR S22
24. S19 AND S23

Notes: Date limited 2001 to date of search

Database: CINAHL
Host: EBSCO
Issue: n/a
Date Searched: 28/6/2021
Searcher: SB
Hits: 671
Strategy:

1. TI ((return* OR back) N2 work*) OR AB ((return* OR back) N2 work*)
2. TI ( return* N2 (occupation* or employ*) ) OR AB ( return* N2 (occupation* or employ*) )
3. (MH "Job Re-Entry")
4. TI ( (reentry or re entry or reenter* or "re enter*") N2 work* ) OR AB ( (reentry or re entry or reenter* or "re enter*") N2 work* )
5. TI ( (reentry or re entry or reenter* or "re enter*") N2 (occupation* or employ*) ) OR AB ( (reentry or re entry or reenter* or "re enter*") N2 (occupation* or employ*) )
6. TI ( (barrier* or facilitator*) N1 (employ* or occupation* or work*) ) OR AB ( (barrier* or facilitator*) N1 (employ* or occupation* or work*) )
7. TI "vocational rehabilitation" OR AB "vocational rehabilitation"
8. TI ((work OR occupational) N0 rehabilitation) OR AB ((work OR occupational) N0 rehabilitation)
9. TI “disability management” OR AB “disability management”
10. (MH "Rehabilitation, Vocational+")
11. S1 OR S2 OR S3 OR S4 OR S5 OR S6 OR S7 OR S8 OR S9 OR S10
12. TI (( sick* N1 (leave or absence) ) OR case management) OR AB (( sick* N1 (leave or absence) ) OR “case management”)
13. (MH "Sick Leave")
14. S12 OR S13
15. TI ( occupational N1 (health or medicine or therap*) ) OR AB ( occupational N1 (health or medicine or therap*) )
16. (MH "Occupational Health+")
17. S15 OR S16
18. S14 AND S17
19. S11 OR S18
20. TI ( (cochrane or cost or effectiveness or implementation or rapid or systematic or "state of the art" or umbrella) N1 (overview* or review* or synthes*) ) OR AB ( (cochrane or cost or effectiveness or implementation or rapid or systematic or "state of the art" or umbrella) N1 (overview* or review* or synthes*) )
21. TI ( "meta analy*" or metaanaly* or metasynthe* or "meta synthe*" ) OR AB ( "meta analy*" or metaanaly* or metasynthe* or "meta synthe*" )
22. TI "review* of reviews" OR AB "review* of reviews"
23. S20 OR S21 OR S22
24. S19 AND S23

Notes: date limited 2001 to date of search

Database: EconLit
Host: EBSCO
Issue: n/a
Date Searched: 28/6/2021
Searcher: SB
Hits: 1
Strategy:

1. TI ((return* OR back) N2 work*) OR AB ((return* OR back) N2 work*)
2. TI ( return* N2 (occupation* or employ*) ) OR AB ( return* N2 (occupation* or employ*) )
3. TI ( (reentry or re entry or reenter* or "re enter*") N2 work* ) OR AB ( (reentry or re entry or reenter* or "re enter*") N2 work* )
4. TI ( (reentry or re entry or reenter* or "re enter*") N2 (occupation* or employ*) ) OR AB ( (reentry or re entry or reenter* or "re enter*") N2 (occupation* or employ*) )
5. TI ( (barrier* or facilitator*) N1 (employ* or occupation* or work*) ) OR AB ( (barrier* or facilitator*) N1 (employ* or occupation* or work*) )
6. TI "vocational rehabilitation" OR AB "vocational rehabilitation"
7. TI ((work OR occupational) N0 rehabilitation) OR AB ((work OR occupational) N0 rehabilitation)
8. TI "disability management" OR AB "disability management"
9. S1 OR S2 OR S3 OR S4 OR S5 OR S6 OR S7 OR S8
10. TI (( sick* N1 (leave or absence) ) OR “case management”) OR AB (( sick* N1 (leave or absence) ) OR “case management”)
11. TI ( occupational N1 (health or medicine or therap*) ) OR AB ( occupational N1 (health or medicine or therap*) )
12. S10 AND S11
13. S9 OR S12
14. TI ( (cochrane or cost or effectiveness or implementation or rapid or systematic or "state of the art" or umbrella) N1 (overview* or review* or synthes*) ) OR AB ( (cochrane or cost or effectiveness or implementation or rapid or systematic or "state of the art" or umbrella) N1 (overview* or review* or synthes*) )
15. TI ( "meta analy*" or metaanaly* or metasynthe* or "meta synthe*" ) OR AB ( "meta analy*" or metaanaly* or metasynthe* or "meta synthe*" )
16. TI "review* of reviews" OR AB "review* of reviews"
17. S14 OR S15 OR S16
18. S13 AND S17

Database: Epistemonikos
Host: [www.epistemonikos.org/en/](http://www.epistemonikos.org/en/)
Issue: n/a
Date Searched: 28/6/2021
Searcher: SB
Hits: 291
Strategy:

1. "return to work"
2. return AND (occupation OR employ*)
3. (title:((rentry OR "re entry" OR "re enter" AND (work OR employ* OR occupation*))) OR abstract:((rentry OR "re entry" OR "re enter" AND (work OR employ* OR occupation*))))
4. (title:("vocational rehabilitation") OR abstract:("vocational rehabilitation"))
5. #1 OR #2 OR #3 OR #4

Notes: Date limited 2001 to 2021 and Systematic Reviews

Database: Health Management Information Consortium (HMIC)
Host: Ovid
Issue: 1979 to May 2021
Date Searched: 28/6/2021
Searcher: SB
Hits: 19
Strategy:

1. (return* adj3 work*).tw.
2. "back to work".tw.
3. (return* adj3 (occupation* or employ*)).tw.
4. ((reentry or re entry or reenter* or "re enter*") adj3 work*).tw.
5. ((reentry or re entry or reenter* or "re enter*") adj3 (occupation* or employ*)).tw.
6. ((barrier* or facilitator*) adj2 (employ* or occupation* or work*)).tw.
7. "vocational rehabilitation".tw.
8. "work rehabilitation".tw.
9. "occupational rehabilitation".tw.
10. “disability management”.tw
11. or/1-10
12. (sick* adj2 (leave or absence)).tw.
13. “case management”.tw
14. 12 or 13
15. (occupational adj2 (health or medicine or therap*)).tw.
16. 14 and 15
17. 11 or 16
18. ((cochrane or cost or effectiveness or implementation or rapid or systematic or "state of the art" or umbrella) adj2 (overview* or review* or synthes*)).tw.
19. ("meta analy*" or metaanaly* or metasynthe* or "meta synthe*").tw.
20. "review* of reviews".tw.
21. or/18-20
22. 17 and 21

Database: MEDLINE
Host: Ovid
Issue: 1946 to June 25, 2021
Date Searched: 28/6/2021
Searcher: SB
Hits: 1125
Strategy:

1. (return* adj3 work*).tw.
2. "back to work".tw.
3. (return* adj3 (occupation* or employ*)).tw.
4. Return to Work/
5. ((reentry or re entry or reenter* or "re enter*") adj3 work*).tw.
6. ((reentry or re entry or reenter* or "re enter*") adj3 (occupation* or employ*)).tw.
7. ((barrier* or facilitator*) adj2 (employ* or occupation* or work*)).tw.
8. "vocational rehabilitation".tw.
9. "work rehabilitation".tw.
10. "occupational rehabilitation".tw.
11. Rehabilitation, Vocational/
12. “disability management”.tw
13. or/1-12
14. (sick* adj2 (leave or absence)).tw.
15. “case management”.tw
16. Sick Leave/
17. or/14-16
18. (occupational adj2 (health or medicine or therap*)).tw.
19. Occupational Health/
20. Occupational Medicine/
21. Occupational Therapy/
22. or/18-21
23. 17 and 22
24. 13 or 23
25. ((cochrane or cost or effectiveness or implementation or rapid or systematic or "state of the art" or umbrella) adj2 (overview* or review* or synthes*)).tw.
26. ("meta analy*" or metaanaly* or metasynthe* or "meta synthe*").tw.
27. "review* of reviews".tw.
28. systematic review.pt.
29. meta-analysis.pt.
30. or/25-29
31. 24 and 30

Notes: date limited 2001 to date of search

Database: Science Citation Index; Social Citation Index; Conference Proceedings
Host: Web of Science
Issue: n/a
Date Searched:
Searcher: SB
Hits: 1326
Strategy:

1. TOPIC: ((return* or back) near/2 work*)
2. TOPIC: (return* near/2 (occupation* or employ*) )
3. TOPIC: ((reentry or "re entry" or reenter* or "re enter*") near/2 work*)
4. TOPIC: ((reentry or "re entry" or reenter* or "re enter*") near/2 (occupation* or employ*) )
5. TOPIC: ((barrier* or facilitator*) near/1 (employ* or occupation* or work*) )
6. TOPIC: ("vocational rehabilitation")
7. TOPIC: ("work rehabilitation")
8. TOPIC: ("occupational rehabilitation")
9. TOPIC:(“disability management”)
10. #9 OR #8 OR #7 OR #6 OR #5 OR #4 OR #3 OR #2 OR #1
11. TOPIC: (sick* near/1 (leave or absence) )
12. TOPIC:(“case management”)
13. TOPIC: (occupational near/1 (health or medicine or therap*) )
14. (#11 OR #12) AND #13
15. TS=((cochrane or cost or effectiveness or implementation or rapid or systematic or "state of the art" or umbrella) near/1 (overview* or review* or synthes*) )
16. TOPIC: ("meta analy*" or metaanaly* or metasynthe* or "meta synthe*")
17. TOPIC: ("review* of reviews")
18. #17 OR #16 OR #15
19. #14 OR #10
20. #18 AND #19

Notes: Date limited 2001 to date of search

*Table 5: Number of unique and de-duplicated records retrieved*

| **Database** | **Results** |
| --- | --- |
| Cochrane Database of Systematic Reviews | 112 |
| Business Source Complete | 37 |
| CINAHL | 671 |
| EconLit | 1 |
| Epistemonikos | 291 |
| HMIC | 19 |
| MEDLINE | 1125 |
| SCI; SSCI; CP | 1326 |
| **Total records retrieved** | **3582** |
| **Duplicate records** | **1603** |
| **Unique records retrieved** | **1979** |

### Web searches

Resource: Google Scholar
URL: <https://scholar.google.co.uk/>
Date Searched: 6/7/2021
Searcher: SB
Hits: 1000
Strategy:

Keyword field: (“return to work” OR “vocational rehabilitation”)

Title field: (“systematic review” OR “evidence synthesis”)

Notes: date limited 2001-2021; searched via Harzing’s Publish or Perish; de-duplicated against bibliographic database results (total unique results = 518)

Resources: Google Search
URL: [www.google.co.uk](http://www.google.co.uk)
Date Searched: 13/7/2021
Searcher: SB
Strategy:

"return to work" ("multi disciplinary" OR multidisciplinary) (report OR review) 315 hits

"vocational rehabilitation" ("multi disciplinary" OR multidisciplinary) (report OR review) 312 hits

Notes: we used the settings menu to change the number of results per page to 100 and screened to the last page of results.

### Websites

Website: Campbell Collaboration
URL: <https://www.campbellcollaboration.org/better-evidence.html>
Date Searched:15/7/2021
Searcher: SB
Strategy:

Search 1: return to work 7 hits

Search 2: occupational health 2 hits

Search 3: vocational rehabilitation 2 hits

Notes: Search carried out in full text keyword search box. All results exported to Endnote. 2 duplicates deleted.

Resource: Health and Safety Executive
URL: <https://www.hse.gov.uk/>
Date Searched: 7/7/2021
Searcher: SB
Strategies:

*Website searches:*

“return to work” 16 hits (publications tab)

100 hits (research tab)

“vocational rehabilitation” 0 hits (publications tab)

26 hits (research tab)

*Website searches via Google Search:*

"return to work" (report OR review) site:hse.gov.uk/ 276 hits (screened first 100 which repeated the results retrieved by the website searches)

"vocational rehabilitation" (report OR review) site:hse.gov.uk/ 68 hits

Notes: Google searches were set up to retrieve 100 results per page.

Resource: HSE Solutions
URL: <https://www.hsl.gov.uk/>
Date Searched: 7/7/2021
Searcher: SB
Strategies:

*Website searches:*

“return to work” 15 hits (search limited to Exact phrase)

“vocational rehabilitation” 1 hit (search limited Exact phrase)

*Website searches via Google Search:*

"return to work" (report OR review) site:hsl.gov.uk/ 22 hits

"vocational rehabilitation" (report OR review) site:hsl.gov.uk/ 2 hits

Resource: NHS Health at Work Network
URL: <https://www.nhshealthatwork.co.uk/>
Date Searched: 7/7/2021
Searcher: SB
Strategy:

*Website searches*

“return to work” 0 hits

“vocational rehabilitation” 0 hits

*Website searches via Google Search:*

"return to work" (report OR review) site:nhshealthatwork.co.uk 117 hits

"vocational rehabilitation" (report OR review) site:nhshealthatwork.co.uk 11 hits

Resource: Society of Occupational Medicine
URL: <https://www.som.org.uk/>
Date Searched: 7/7/2021
Searcher: SB
Strategy:

*Website searches:*

“return to work” 58 hits

“vocational rehabilitation” 10 hits

*Website searches via Google Search:*

"return to work" (report OR review) site:som.org.uk 106 hits

"vocational rehabilitation" (report OR review) site:som.org.uk 14 hits

Resource: Faculty of Occupational Health Nursing
URL: <https://www.fohn.org.uk/>
Date Searched: 7/7/2021
Searcher: SB
Strategy:

*Website searches:*

“return to work” 5 hits

“vocational rehabilitation” 0 hits

*Website searches via Google Search:*

"return to work" (report OR review) site: fohn.org.uk/ 79 hits

"vocational rehabilitation" (report OR review) site: fohn.org.uk/ 78 hits

Resource: Council for Work and Health
URL: <https://www.councilforworkandhealth.org.uk/>
Date Searched:
Searcher:
Strategy:

*Website searches:*

Browsed Projects and Resources tabs

*Website searches via Google Search:*

"return to work" (report OR review) site: councilforworkandhealth.org.uk/ 81 hits

"vocational rehabilitation" (report OR review) site: councilforworkandhealth.org.uk/ 64 hits

## 2 Summary data extracted from each included review

|  | **Description** |
| --- | --- |
| **Author, date** | E.g Axen 2020 |
| **Review title** |  |
| **Review aim** | As reported in the abstract or end of introduction |
| **Type of review** | Most common review types included systematic and scoping reviews |
| **Type of primary studies included in review** | As described in the review inclusion criteria or results section |
| **Description of intervention and how it may work** | This included any theory, rationale or model supporting the intervention provided within the background and/or methods section of the review |
| **Outcome of interest/How RTW measured** | Brief description of outcome of interest (RTW or cost) and how this was measured |
| **Synthesis method** | Method used to synthesise data within the review, including meta-analysis, narrative or ‘best-evidence’ synthesis or descriptive analysis |
| **Queries regarding relevance of review PICO to our umbrella review** | Any queries regarding how the population, intervention, outcome or setting of the review aligned with the inclusion criteria of our umbrella review were identified here. These queries often arose through a lack of/unclear reporting of required detail within the included review |
| **Review inclusion/ exclusion criteria** | From the methods section of each included review |
| **Review quality: Is approach to searching clearly defined, systematic and transparent?** | One criterion from the CEESAT. This item required that all search terms, Boolean operators (‘AND’, ‘OR’ etc.) and wildcards were clearly stated so that the exact search is repeatable by a third party  AND  There was information about the sources searched, together with dates of search [but no limitations justified (e.g. language, or publication date, no grey literature searches)] |
| **Review quality: Is search comprehensive?** | The original item from the CEESAT requires that sources of articles searched capture both conventionally published scientific literature and grey literature using a combination of databases, search engines and specialist websites (may also be informed by stakeholders) or limitations are fully justified.  However, for the purpose of this review we modified these criteria to require a minimum of 3 databases AND at least one other. Specific searches for grey literature were NOT necessary |
| **Review quality: Does the review critically appraise each study?** | This CEESAT item states that an effort should be made to identify relevant sources of bias (threats to internal and external validity)  AND  Each type of bias or threat to internal and external validity was assessed individually for all included studies and reported on a critical appraisal sheet |
| **Review quality: During critical appraisal is an effort made to minimise subjectivity?** | The original item from the CEESAT requires that an effort was made to minimise subjectivity by predefining critical appraisal process in a protocol  AND  At least two people critically appraised each study but not independently (e.g. second person aware of first person’s decision) OR a subset of studies was appraised by at least two people independently and disagreements and process of resolution reported.  We modified this item: the review did not need to check protocol; did NOT need mention of process for resolving disagreements AS LONG AS it is clearly stated that two reviewers performed appraisal independently |
| **Overall quality rating** | High quality = all four quality criteria listed above were met;  Moderate = 2-3 of the four quality criteria listed above were met;  Low = a maximum of one of the four quality criteria listed above were met |
| **Relevance of aim of review to umbrella review** | This encompasses how the aim of the included review relates to the aim and PICO of our umbrella review.  High = Aim of systematic review directly relevant to our umbrella review, with potentially just one query around population (i.e. were they employed) or intervention (i.e. was it delivered by a multidisciplinary team and in conjunction with the workplace?);  Medium = Two queries, or aim of study not completely compatible with the aims of our review;  Low = Two to three queries regarding review inclusion criteria and/or limited quantity of relevant included primary studies |
| **Number of relevant/total number of included studies** | The number of primary studies included within the review which, based on information provided in the review, appeared to meet the inclusion criteria of our umbrella review. This information was extracted for reviews which were of high or medium relevance to our umbrella review.  The total number of included primary studies was also extracted for these reviews. |

## 3 AMSTAR-2 Quality Appraisal items

|  | **Question** | **Option** |
| --- | --- | --- |
| 1. | Did the research questions and inclusion criteria for the review include the components of PICO? | Yes/no |
| 2.* | Did the report of the review contain an explicit statement that the review methods were established prior to the conduct of the review and did the report justify any significant deviations from the protocol? | Yes; partial yes; no |
| 3. | Did the review authors explain their selection of the study designs for inclusion in the review? | Yes/no |
| 4.* | Did the review authors use a comprehensive literature search strategy? | Yes; partial yes; no |
| 5. | Did the review authors perform study selection in duplicate? | Yes/no |
| 6. | Did the review authors perform data extraction in duplicate? | Yes/no |
| 7. | Did the review authors provide a list of excluded studies and justify the exclusions? | Yes; partial yes; no |
| 8. | Did the review authors describe the included studies in adequate detail? | Yes; partial yes; no |
| 9.* | Did the review authors use a satisfactory technique for assessing the risk of bias (RoB) in individual studies that were included in the review? | Yes; partial yes; no  Includes only NRSI/ RCTs |
| 10. | Did the review authors report on the sources of funding for the studies included in the review? | Yes/no |
| 11.* | If meta-analysis was performed did the review authors use appropriate methods for statistical combination of results? | Yes/no  No meta-analysis conducted |
| 12. | If meta-analysis was performed, did the review authors assess the potential impact of RoB in individual studies on the results of the meta-analysis or other evidence synthesis? | Yes/no  No meta-analysis conducted |
| 13.* | Did the review authors account for RoB in individual studies when interpreting/ discussing the results of the review? | Yes/no |
| 14. | Did the review authors provide a satisfactory explanation for, and discussion of, any heterogeneity observed in the results of the review? | Yes/no |
| 15. | If they performed quantitative synthesis did the review authors carry out an adequate investigation of publication bias (small study bias) and discuss its likely impact on the results of the review? | Yes/no  No meta-analysis conducted |
| 16. | Did the review authors report any potential sources of conflict of interest, including any funding they received for conducting the review? | Yes/no |

*indicates critical domains used within this review to determine the overall study rating

**Rating overall confidence in the results of the review**

**High** No or one non-critical weakness: the SR provides an accurate and comprehensive summary of the results of the available studies that address the question of interest

**Moderate** More than one non-critical weakness**: the SR has more than one weakness but no critical flaws. It may provide an accurate summary of the results of the available studies that were included in the review

**Low** One critical flaw with or without non-critical weaknesses: the review has a critical flaw and may not provide an accurate and comprehensive summary of the available studies that address the question of interest

**Critically low** More than one critical flaw with or without non-critical weaknesses: the review has more than one critical flaw and should not be relied on to provide an accurate and comprehensive summary of the available studies

**Multiple non-critical weaknesses may diminish confidence in the review and may mean it is appropriate to move the overall appraisal from moderate to low confidence.

## 4 List of studies included in EGM

Abbott, R., Bethel, A., Rogers, M., Whear, R., Orr, N., Shaw, L., Stein, K., & Thompson Coon, J. (2022). Characteristics, quality and volume of the first 5 months of the COVID-19 evidence synthesis infodemic: a meta-research study. *BMJ Evid Based Med*, *27*(3), 169-177. <https://doi.org/10.1136/bmjebm-2021-111710>

Axen, I., Bjork Bramberg, E., Vaez, M., Lundin, A., & Bergstrom, G. (2020). Interventions for common mental disorders in the occupational health service: a systematic review with a narrative synthesis. *International archives of occupational and environmental health*, *93*(7), 823-838, Article <https://doi.org/https://dx.doi.org/10.1007/s00420-020-01535-4>

Bernaers, L., Cnockaert, E., Braeckman, L., Mairiaux, P., & Willems, T. M. (2022). Disability and return to work after a multidisciplinary intervention for (sub)acute low back pain: A systematic review. *Clinical rehabilitation*, 2692155221146447, Article 0. <https://doi.org/https://dx.doi.org/10.1177/02692155221146447>

Brewer, S., King, E., Amick, B., Delclos, G., Spear, J., Irvin, E., Mahood, Q., Lee, L., Lewis, C., & Tetrick, L. (2007). A systematic review of injury/illness prevention and loss control programs (IPC). *Toronto: Institute for Work & Health*.

Burdorf, A., Porru, F., & Rugulies, R. (2020). The COVID-19 (Coronavirus) pandemic: consequences for occupational health. *Scandinavian Journal of Work, Environment & Health*, *46*(3), 229-230.

Carroll, C., Rick, J., Pilgrim, H., Cameron, J., & Hillage, J. (2010). Workplace involvement improves return to work rates among employees with back pain on long-term sick leave: a systematic review of the effectiveness and cost-effectiveness of interventions. *Disability and rehabilitation*, *32*(8), 607-621.

Chambers, D., Wade, R., & Wilson, P. (2012). *Training manual for selecting reviews and writing abstracts for the Database of Abstracts of Reviews of Effects (DARE)*.

Cochrane, A., Higgins, N. M., FitzGerald, O., Gallagher, P., Ashton, J., Corcoran, O., & Desmond, D. (2017). Early interventions to promote work participation in people with regional musculoskeletal pain: a systematic review and meta-analysis. *Clinical rehabilitation*, *31*(11), 1466-1481, Article <https://doi.org/https://dx.doi.org/10.1177/0269215517699976>

Cullen, K. L., Irvin, E., Collie, A., Clay, F., Gensby, U., Jennings, P. A., Hogg-Johnson, S., Kristman, V., Laberge, M., McKenzie, D., Newnam, S., Palagyi, A., Ruseckaite, R., Sheppard, D. M., Shourie, S., Steenstra, I., Van Eerd, D., & Amick, B. C. (2018). Effectiveness of Workplace Interventions in Return-to-Work for Musculoskeletal, Pain-Related and Mental Health Conditions: An Update of the Evidence and Messages for Practitioners. *Journal of Occupational Rehabilitation*, *28*(1), 1-15. <https://doi.org/10.1007/s10926-016-9690-x>

Department for Business, Innovation and Skills. (2011). *Default Retirement Age to end this year*. Retrieved 26 May from <https://www.gov.uk/government/news/default-retirement-age-to-end-this-year>

Emerson, E., Fortune, N., Aitken, Z., Hatton, C., Stancliffe, R., & Llewellyn, G. (2020). The wellbeing of working-age adults with and without disability in the UK: Associations with age, gender, ethnicity, partnership status, educational attainment and employment status. *Disabil Health J*, *13*(3), 100889. <https://doi.org/10.1016/j.dhjo.2020.100889>

Evidence, C. f. E. (2018). *Guidelines and Standards for Evidence synthesis in Environmental Management.* . Retrieved 12.08.22 from

Evidence, C. f. E. (2020). *The Collaboration for Environmental Evidence Synthesis Appraisal Tool (CEESAT). Version 2.1.* <https://environmentalevidence.org/ceeder/about-ceesat/>

Franche, R., Cullen, K., Clarke, J., Irvin, E., Sinclair, S., & Frank, J. (2005). Workplace-based return-to-work interventions: a systematic review of the quantitative literature. *Journal of Occupational Rehabilitation*, *15*(4), 607-631, Article <https://doi.org/10.1007/s10926-005-8038-8>

Gaillard, A., Sultan-Taieb, H., Sylvain, C., & Durand, M. J. (2020). Economic evaluations of mental health interventions: A systematic review of interventions with work-focused components. *Safety Science*, *132*, Article <https://doi.org/10.1016/j.ssci.2020.104982>

Gehanno, J. F., Rollin, L., Le Jean, T., Louvel, A., Darmoni, S., & Shaw, W. (2009). Precision and recall of search strategies for identifying studies on return-to-work in Medline. *J Occup Rehabil*, *19*(3), 223-230. <https://doi.org/10.1007/s10926-009-9177-0>

Gensby, U., Labriola, M., Irvin, E., Amick, B. C., 3rd, & Lund, T. (2014). A classification of components of workplace disability management programs: results from a systematic review. *J Occup Rehabil*, *24*(2), 220-241. <https://doi.org/10.1007/s10926-013-9437-x>

Giorgi, G., Lecca, L. I., Alessio, F., Finstad, G. L., Bondanini, G., Lulli, L. G., Arcangeli, G., & Mucci, N. (2020). COVID-19-related mental health effects in the workplace: a narrative review. *International journal of environmental research and public health*, *17*(21), 7857.

Godeau, D., Petit, A., Richard, I., Roquelaure, Y., & Descatha, A. (2021). Return-to-work, disabilities and occupational health in the age of COVID-19. *Scandinavian Journal of Work, Environment & Health*, *47*(5), 408.

Hassard, J., Jain, A., & Leka, S. (2021). *International Comparison of Occupational Health Systems and Provisions: A comparative case study review*.

Health and Safety Executive. (n.d.). *Working days lost in Great Britain*. Retrieved 17 June from <https://www.hse.gov.uk/statistics/dayslost.htm>

Heathcote, K., Wullschleger, M., & Sun, J. (2019). The effectiveness of multi-dimensional resilience rehabilitation programs after traumatic physical injuries: a systematic review and meta-analysis. *Disability and rehabilitation*, *41*(24), 2865-2880, Article <https://doi.org/https://dx.doi.org/10.1080/09638288.2018.1479780>

Hoefsmit, N., Houkes, I., & Nijhuis, F. J. (2012). Intervention characteristics that facilitate return to work after sickness absence: a systematic literature review. *J Occup Rehabil*, *22*(4), 462-477. <https://doi.org/10.1007/s10926-012-9359-z>

Ishimaru, T., Chimed-Ochir, O., Arphorn, S., & Fujino, Y. (2021). Effectiveness of fitness for work interventions for workers with low back pain: A systematic review. *Journal of occupational health*, *63*(1), e12261, Article 0. <https://doi.org/https://dx.doi.org/10.1002/1348-9585.12261>

Kniffin, K. M., Narayanan, J., Anseel, F., Antonakis, J., Ashford, S. P., Bakker, A. B., Bamberger, P., Bapuji, H., Bhave, D. P., Choi, V. K., Creary, S. J., Demerouti, E., Flynn, F. J., Gelfand, M. J., Greer, L. L., Johns, G., Kesebir, S., Klein, P. G., Lee, S. Y., Ozcelik, H., Petriglieri, J. L., Rothbard, N. P., Rudolph, C. W., Shaw, J. D., Sirola, N., Wanberg, C. R., Whillans, A., Wilmot, M. P., & Vugt, M. V. (2021). COVID-19 and the workplace: Implications, issues, and insights for future research and action. *Am Psychol*, *76*(1), 63-77. <https://doi.org/10.1037/amp0000716>

Kojimahara, N., Muto, G., Teruya, K., Nogawa, K., & Doki, S. (2020). Return-to-work in Japanese Occupational Health Settings: A Systematic Review and Recommendations. *Tokyo Women's Medical University Journal*, *4*, 9-16.

Lefever, M., Decuman, S., Perl, F., Braeckman, L., & Van de Velde, D. (2018). The efficacy and efficiency of Disability Management in job-retention and job-reintegration. A systematic review. *Work (Reading, Mass.)*, *59*(4), 501-534, Article <https://doi.org/https://dx.doi.org/10.3233/WOR-182709>

Liberati, A., Altman, D. G., Tetzlaff, J., Mulrow, C., Gotzsche, P. C., Ioannidis, J. P., Clarke, M., Devereaux, P. J., Kleijnen, J., & Moher, D. (2009). The PRISMA statement for reporting systematic reviews and meta-analyses of studies that evaluate health care interventions: explanation and elaboration. *PLoS Med*, *6*(7), e1000100. <https://doi.org/10.1371/journal.pmed.1000100>

Mandal, S., Barnett, J., Brill, S. E., Brown, J. S., Denneny, E. K., Hare, S. S., Heightman, M., Hillman, T. E., Jacob, J., Jarvis, H. C., Lipman, M. C. I., Naidu, S. B., Nair, A., Porter, J. C., Tomlinson, G. S., Hurst, J. R., & Group, A. R. C. S. (2020). 'Long-COVID': a cross-sectional study of persisting symptoms, biomarker and imaging abnormalities following hospitalisation for COVID-19. *Thorax*, *76*(4), 396-398. <https://doi.org/10.1136/thoraxjnl-2020-215818>

Mikkelsen, M. B., & Rosholm, M. (2018). Systematic review and meta-analysis of interventions aimed at enhancing return to work for sick-listed workers with common mental disorders, stress-related disorders, somatoform disorders and personality disorders. *Occupational and environmental medicine*, *75*(9), 675-686, Article <https://doi.org/https://dx.doi.org/10.1136/oemed-2018-105073>

Neverdal, C. (2015). *Effectiveness of workplace nterventions targeting return to work in patients with low back and neck pain: a systematic review*

NHS Confederation. (2017). *NHS statistics, facts and figures*. Retrieved 20th September from <http://www.nhsconfed.org/resources/key-statistics-on-the-nhs>

NICE. (2019). Workplace Health: Long-Term Sickness Absence and Capability to Work. NICE Guideline [NG146]. In: The National Institute for Health and Care Excellence.

Nieuwenhuijsen, K., Verbeek, J. H., Neumeyer-Gromen, A., Verhoeven, A. C., Bultmann, U., & Faber, B. (2020). Interventions to improve return to work in depressed people. *The Cochrane database of systematic reviews*, *10*, CD006237, Article <https://doi.org/https://dx.doi.org/10.1002/14651858.CD006237.pub4> (Update of: Cochrane Database Syst Rev. 2014 Dec 03;(12):CD006237; PMID: 25470301 [<https://www.ncbi.nlm.nih.gov/pubmed/25470301>])

Oakman, J., Keegel, T., Kinsman, N., & Briggs, A. M. (2016). Persistent musculoskeletal pain and productive employment; a systematic review of interventions. *Occupational and environmental medicine*, *73*(3), 206-214, Article <https://doi.org/https://dx.doi.org/10.1136/oemed-2015-103208>

Odeen, M., Magnussen, L. H., Maeland, S., Larun, L., Eriksen, H. R., & Tveito, T. H. (2013). Systematic review of active workplace interventions to reduce sickness absence. *Occupational medicine (Oxford, England)*, *63*(1), 7-16, Article <https://doi.org/https://dx.doi.org/10.1093/occmed/kqs198>

Office for National Statistics. (2021a, 18 May 2021). *Dataset: A08: Labour market status of disabled people*. Retrieved 17 June from <https://www.ons.gov.uk/employmentandlabourmarket/peopleinwork/employmentandemployeetypes/datasets/labourmarketstatusofdisabledpeoplea08>

Office for National Statistics. (2021b). *Dataset: Sickness absence in the UK labour market*. Retrieved 17 June from <https://www.ons.gov.uk/employmentandlabourmarket/peopleinwork/employmentandemployeetypes/datasets/sicknessabsenceinthelabourmarket>

Palmer, K. T., Harris, E. C., Linaker, C., Barker, M., Lawrence, W., Cooper, C., & Coggon, D. (2012). Effectiveness of community- and workplace-based interventions to manage musculoskeletal-related sickness absence and job loss: a systematic review. *Rheumatology (Oxford, England)*, *51*(2), 230-242, Article <https://doi.org/https://dx.doi.org/10.1093/rheumatology/ker086>

Petticrew, M., Song, F., Wilson, P., & Wright, K. (1999). QUALITY-ASSESSED REVIEWS OF HEALTH CARE INTERVENTIONS AND THE DATABASE OF ABSTRACTS OF REVIEWS OF EFFECTIVENESS (DARE). *International Journal of Technology Assessment in Health Care*, *15*(4), 671-678. <https://doi.org/10.1017/S0266462399015469>

Schaafsma, F., Schonstein, E., Whelan, K. M., Ulvestad, E., Kenny, D. T., & Verbeek, J. H. (2013). Physical conditioning as part of a return to work strategy to reduce sickness absence for workers with back pain. *Cochrane Database of Systematic Reviews*(8), N.PAG-N.PAG, Article <https://doi.org/10.1002/14651858.CD001822>

Schandelmaier, S., Ebrahim, S., Burkhardt, S. C., de Boer, W. E., Zumbrunn, T., Guyatt, G. H., Busse, J. W., & Kunz, R. (2012). Return to work coordination programmes for work disability: a meta-analysis of randomised controlled trials. *PloS one*, *7*(11), e49760, Article <https://doi.org/10.1371/journal.pone.0049760>

Shaw, E., Nunns, M., Briscoe, S., Melendez-Torres, G. J., Garside, R., & Thompson-Coon, J. (2021). *What multi-disciplinary delivery models for Occupational Health services are effective for whom? An umbrella review*. <https://osf.io/qa7n2/>

Shaw, E., Nunns, M., Briscoe, S., Turner, M., Garside, R., Melendez-Torres, G. J., Liabo, K., & Thompson Coon, J. (2021). *Optimising prescribing of drugs to prevent CVD and drugs that cause dependency: an evidence-gap map. Final report*. <https://ore.exeter.ac.uk/repository/handle/10871/128337>

Shaw, E., Nunns, M., Spicer, S., Lawal, H., Briscoe, S., Melendez-Torres, G. J., Garside, R., Liabo, K., & Thompson-Coon, J. (2022). *What multi-disciplinary delivery models for Occupational Health services are effective for whom? An umbrella review*. <https://ore.exeter.ac.uk/repository/handle/10871/131835>

Shea, B. J., Reeves, B. C., Wells, G., Thuku, M., Hamel, C., Moran, J., Moher, D., Tugwell, P., Welch, V., Kristjansson, E., & Henry, D. A. (2017). AMSTAR 2: a critical appraisal tool for systematic reviews that include randomised or non-randomised studies of healthcare interventions, or both. *BMJ*, *358*, j4008. <https://doi.org/10.1136/bmj.j4008>

Sinclair, R. R., Allen, T., Barber, L., Bergman, M., Britt, T., Butler, A., Ford, M., Hammer, L., Kath, L., & Probst, T. (2020). Occupational health science in the time of COVID-19: Now more than ever. In (Vol. 4, pp. 1-22): Springer.

Snodgrass, J. (2011). Effective occupational therapy interventions in the rehabilitation of individuals with work-related low back injuries and illnesses: a systematic review. *Am J Occup Ther*, *65*(1), 37-43. <https://doi.org/10.5014/ajot.2011.09187>

The Council for Work and Health. (2016). *Planning for the future: Implications for occupational health; delivery and training*. <https://www.councilforworkandhealth.org.uk/wp-content/uploads/2018/07/Final-Report-Planning-the-Future-Implications-for-OH-Proof-2.pdf>

The Council for Work and Health, & Syngentis. (2014). *Planning the future: Delivering a vision of good work and health in the UK for the next 5-20 years and the professional resources to deliver it*. <https://www.som.org.uk/fileadmin/user_upload/Office/docs/Planning_the_Future-OH_and_its_Workforce-April_2014.pdf>.

Thomas, J., Brunton, J., & Graziosi, S. (2010). EPPI-Reviewer 4.0: software for research synthesis. *EPPI-Centre Software. London: Social Science Research Unit, Institute of Education*.

Tindle, A., Adams, L., Kearney, I., Hazel, Z., & Stroud, S. (2020). *Understanding the provision of occupational health and work-related musculoskeletal services*. Department for Work & Pensions.

Tingulstad, A., Meneses-Echavez, J., Evensen, L. H., Bjerk, M., & Berg, R. C. (2022). Effectiveness of work-related interventions for return to work in people on sick leave: a systematic review and meta-analysis of randomized controlled trials. *Systematic reviews*, *11*(1), 192, Article 0. <https://doi.org/https://dx.doi.org/10.1186/s13643-022-02055-7>

Tompa, E., de Oliveira, C., Dolinschi, R., & Irvin, E. (2008). A systematic review of disability management interventions with economic evaluations. *Journal of Occupational Rehabilitation*, *18*(1), 16-26, Article <https://doi.org/https://dx.doi.org/10.1007/s10926-007-9116-x>

van Geen, J.-W., Edelaar, M. J. A., Janssen, M., & van Eijk, J. T. M. (2007). The long-term effect of multidisciplinary back training: a systematic review. *Spine*, *32*(2), 249-255, Article <http://ovidsp.ovid.com/ovidweb.cgi?T=JS&PAGE=reference&D=med6&NEWS=N&AN=17224822>

van Vilsteren, M., van Oostrom, S. H., de Vet, H. C. W., Franche, R. L., Boot, C. R. L., & Anema, J. R. (2015). Workplace interventions to prevent work disability in workers on sick leave. *Cochrane Database of Systematic Reviews*(10), Article <https://doi.org/10.1002/14651858.CD006955.pub3>

Vandenbroeck, S., Verjans, M., Lambreghts, C., & Godderis, L. (2016). Research review on rehabilitation and return to work. *Research review on rehabilitation and return to work*.

Venning, A., Oswald, T. K., Stevenson, J., Tepper, N., Azadi, L., Lawn, S., & Redpath, P. (2021). Determining what constitutes an effective psychosocial 'return to work' intervention: a systematic review and narrative synthesis. *BMC Public Health*, *21*(1), 2164, Article 0 potential duplicate. <https://doi.org/https://dx.doi.org/10.1186/s12889-021-11898-z>

Verhoef, J. A. C., Bal, M. I., Roelofs, P., Borghouts, J. A. J., Roebroeck, M. E., & Miedema, H. S. (2020). Effectiveness and characteristics of interventions to improve work participation in adults with chronic physical conditions: a systematic review. *Disability and rehabilitation*, 1-16, Article <https://doi.org/10.1080/09638288.2020.1788180>

Vindegaard, N., & Benros, M. E. (2020). COVID-19 pandemic and mental health consequences: Systematic review of the current evidence. *Brain Behav Immun*, *89*, 531-542. <https://doi.org/10.1016/j.bbi.2020.05.048>

Vogel, N., Schandelmaier, S., Zumbrunn, T., Ebrahim, S., de Boer, W. E., Busse, J. W., & Kunz, R. (2017). Return-to-work coordination programmes for improving return to work in workers on sick leave. *The Cochrane database of systematic reviews*, *3*, CD011618, Article <https://doi.org/https://dx.doi.org/10.1002/14651858.CD011618.pub2>

Vooijs, M., Leensen, M. C., Hoving, J. L., Wind, H., & Frings-Dresen, M. H. (2015). Interventions to enhance work participation of workers with a chronic disease: a systematic review of reviews. *Occupational and environmental medicine*, *72*(11), 820-826.

Vornholt, K., Villotti, P., Muschalla, B., Bauer, J., Colella, A., Zijlstra, F., Van Ruitenbeek, G., Uitdewilligen, S., & Corbiere, M. (2018). Disability and employment–overview and highlights. *European journal of work and organizational psychology*, *27*(1), 40-55.

Waddell, G., Burton, A. K., & Kendall, N. A. (2008). *Vocational rehabilitation–what works, for whom, and when?(Report for the Vocational Rehabilitation Task Group)* (011703861X).

Welch, V. A., Petkovic, J., Jull, J., Hartling, L., Klassen, T., Kristjansson, E., Pardo, J. P., Petticrew, M., Stott, D. J., & Thomson, D. (2019). Equity and specific populations. *Cochrane Handbook for Systematic Reviews of Interventions*, 433-449.

White, H., Albers, B., Gaarder, M., Kornør, H., Littell, J., Marshall, Z., Mathew, C., Pigott, T., Snilstveit, B., Waddington, H., & Welch, V. (2020). Guidance for producing a Campbell evidence and gap map. *Campbell Syst Rev*, *16*(4), e1125. <https://doi.org/10.1002/cl2.1125>

White, M. I., Dionne, C. E., Wärje, O., Koehoorn, M., Wagner, S., Schultz, I. Z., Koehn, C., Williams-Whitt, K., Harder, H., & Pasca, R. (2016). Physical activity and exercise interventions in the workplace impacting work outcomes: A stakeholder-centered best evidence synthesis of systematic reviews. *The international journal of occupational and environmental medicine*, *7*(2), 61.

Yogarajah, N. (2019). *The Future of the Occupational Health Workforce*. Retrieved 27 May from <https://www.som.org.uk/sites/som.org.uk/files/The_future_of_the_OH_workforce.pdf>

## 5 Link to online interactive EGM

The map is intended as an interactive resource and we suggest that readers navigate the evidence and gap map, accessed here ([https://eppi.ioe.ac.uk/cms/Portals/35/Maps/MN_Exeter_Feb22.html](https://eur03.safelinks.protection.outlook.com/?url=https%3A%2F%2Feppi.ioe.ac.uk%2Fcms%2FPortals%2F35%2FMaps%2FMN_Exeter_Feb22.html&data=04%7C01%7CM.P.Nunns%40exeter.ac.uk%7C5633c91e641b490bef4d08d9f7aa523c%7C912a5d77fb984eeeaf321334d8f04a53%7C0%7C0%7C637813133152131956%7CUnknown%7CTWFpbGZsb3d8eyJWIjoiMC4wLjAwMDAiLCJQIjoiV2luMzIiLCJBTiI6Ik1haWwiLCJXVCI6Mn0%3D%7C3000&sdata=7S1iNgIpDn0LuMhhTOmC%2BRRhpfSfustNY5tIEP%2BeLzU%3D&reserved=0)), and browse publications of interest.
