# Supplementary material for: What is the volume, quality and characteristics of evidence relating to the effectiveness and cost‐effectiveness of multi‐disciplinary occupational health interventions aiming to improve work‐related outcomes for employed adults? An evidence and gap map of systematic reviews
Source: Campbell Syst Rev. 2024 May 14;20(2):e1412. doi: 10.1002/cl2.1412 (PMC11094349; doi:10.1002/cl2.1412)
Supplement: Supplementary file 2 — Supporting information. [file CL2-20-e1412-s002.docx]

ABBREVIATIONS AND ACRONYMS

**AMSTAR 2** Assessing the Methodological quality of Systematic Reviews

**CEESAT** The Collaboration for Environmental Evidence Synthesis Appraisal Tool

**DHSC** Department of Health and Social Care

**DWP** Department of Work and Pensions

**EGM** Evidence and Gap Map

**MDT** Multi-disciplinary Team

**NHS** National Health Service

**NHSE-I** National Health Service England and NHS Improvement

**OH** Occupational Health

**OP** Occupational Physician

**OT** Occupational Therapist

**PT** Physio or Physical Therapist

**PHE** Public Health England

**PRISMA** Preferred Reporting Items for Systematic Reviews and Meta-Analyses

**PRP** Policy Research Programme

**RTW** Return To Work

**SW** Social Worker

**UK** United Kingdom
